# Supplementary material for: Non-invasive, vagus nerve stimulation to reduce ileus after colorectal surgery: protocol for a feasibility trial with nested mechanistic studies
Source: BMJ Open. 2021 Jul 21;11(7):e046313. doi: 10.1136/bmjopen-2020-046313 (PMC8296772; doi:10.1136/bmjopen-2020-046313)
Supplement: Supplementary data [file bmjopen-2020-046313supp001.pdf]

Supplementary File

Study Status

The study first began recruitment on 2nd January 2020 and is expected to complete by 31st August 2021. The current protocol version is v1.2 (13th February 2020).

Auditing

The conduct of the trial will be audited monthly by the principal investigator. In line with Good Clinical Practice, a study site folder will be held at each participating recruitment site. This will contain the current study protocol, delegation log outlining responsibilities of all research team members, and all study correspondence. Data relating to recruitment will be uploaded to the NIHR Central Portfolio Management System on a monthly basis.

Adverse Events

The reporting of adverse events will be done in line with Good Clinical Practice. All serious adverse events will be reported to the sponsor within 24 hours of research staff becoming aware of the event. Serious adverse events that are considered to be unexpected and related to the study will be escalated to the research ethics committee.

Study Amendments

If amendments to the study protocol or documents are required, this will be done and communicated in line with Health Research Authority guidelines. All amendments will be categorised and submitted for review by the Research Ethics Committee, as appropriate.

Dissemination Plan

The findings of the study and sub-studies will be disseminated at academic conferences and in peer-reviewed manuscripts. The results will also be summarised in plain English visual abstracts and disseminated to research participants and the public.

Study Sponsor

The sponsor for this study is University of Leeds

Recruitment & Protocol Materials

Study recruitment and protocol materials are available upon request from the corresponding author.
