# Supplementary material for: Non-invasive, vagus nerve stimulation to reduce ileus after colorectal surgery: protocol for a feasibility trial with nested mechanistic studies
Source: BMJ Open. 2021 Jul 21;11(7):e046313. doi: 10.1136/bmjopen-2020-046313 (PMC8296772; doi:10.1136/bmjopen-2020-046313)
Supplement: Supplementary data [file bmjopen-2020-046313supp003.pdf]

UNIVERSITY OF LEEDS

Vagus nerve stimulation to improve bowel function after surgery

Participant Study ID	Patient Initials	Patient Date of Birth

Agreement to the following are required to participate in the study

Please initial the boxes to indicate your agreement:

A. I confirm that I have read the patient information sheet dated [date, version] and have had the opportunity to ask questions. ☐

B. I understand that my participation in all aspects of this study is voluntary and that I may freely withdraw at any time, without giving a reason and without my medical care or rights being affected. I understand that data already collected before withdrawal will be used, unless I request otherwise. ☐

C. I understand that sections of my medical records may be looked at by responsible individuals from the lead study team, the University of Leeds, and from regulatory authorities where it is relevant to my taking part in this research study. ☐

D. I agree to allow the information collected about me and a copy of this consent form to be passed to the University of Leeds upon it being handled securely ☐

E. I agree to allow information or results arising from this study to be used for future medical research on the understanding that my identity will remain anonymous. ☐

F. I agree for my GP to be informed about my participation in this study ☐

G. I agree to be contacted by telephone and/or in writing with information that may be relevant to my taking part in the research using information held at the hospital ☐

H. I agree to take part in the above study. ☐

Chapman SJ, *et al. BMJ Open* 2021; 11:e046313. doi: 10.1136/bmjopen-2020-046313

UNIVERSITY OF LEEDS

Participant Study ID	Patient Initials	Patient Date of Birth

The following criteria are optional and relevant to patients having their operation at St. James's University Hospital (SJUH) only

Do you wish to participate in the optional part of this study (involving blood and fluid tests)? (please tick as appropriate):

☐ Yes (see box) ☐ No ☐ Surgery not at SJUH

*If you ticked **Yes** above, please initial the boxes to indicate your agreement:*

I. I understand that all samples will be stored, analysed, and destroyed at the University of Leeds according to regulations set by the Health Tissue Authority. ☐

J. I know that I may freely withdraw from this optional study without affecting my participation in the main study, my medical care, or my legal rights. ☐

K. I understand that samples will be stored confidentially and that my name and personal details will not be stored with my samples. I understand that samples collected prior to withdrawal will be retained unless I request otherwise. ☐

L. I agree to allow data and samples from this study to be used for future medical research on the understanding that my identity will remain anonymous. ☐

Name of treating hospital/research site
(BLOCK CAPITALS)

Name of participant (BLOCK CAPITALS)	Date (dd/mm/yyyy)	Signature
Name of researcher taking consent (BLOCK CAPITALS)	Date (dd/mm/yyyy)	Signature

One copy of this consent form should be retained by the recruiting team and another copy given to the participant

Chapman SJ, *et al. BMJ Open* 2021; 11:e046313. doi: 10.1136/bmjopen-2020-046313
