# Supplementary material for: Non-invasive, vagus nerve stimulation to reduce ileus after colorectal surgery: protocol for a feasibility trial with nested mechanistic studies
Source: BMJ Open. 2021 Jul 21;11(7):e046313. doi: 10.1136/bmjopen-2020-046313 (PMC8296772; doi:10.1136/bmjopen-2020-046313)
Supplement: Author's manuscript [file bmjopen-2020-046313.draft_revisions.pdf]

BMJ Open is committed to open peer review. As part of this commitment we make the peer review history of every article we publish publicly available.

When an article is published we post the peer reviewers' comments and the authors' responses online. We also post the versions of the paper that were used during peer review. These are the versions that the peer review comments apply to.

The versions of the paper that follow are the versions that were submitted during the peer review process. They are not the versions of record or the final published versions. They should not be cited or distributed as the published version of this manuscript.

BMJ Open is an open access journal and the full, final, typeset and author-corrected version of record of the manuscript is available on our site with no access controls, subscription charges or pay-per-view fees (http://bmjopen.bmj.com).

If you have any questions on BMJ Open's open peer review process please email info.bmjopen@bmj.com

BMJ Open

Non-invasive, vagus nerve stimulation to reduce ileus after colorectal surgery: Protocol for a feasibility trial with nested mechanistic studies

Journal:	*BMJ Open*
Manuscript ID	bmjopen-2020-046313
Article Type:	Protocol
Date Submitted by the Author:	27-Oct-2020

Complete List of Authors:	Chapman, Stephen; University of Leeds, Leeds Institute of Medical Research<br>Naylor, Maureen; West Riding of Yorkshire Ileostomy Association<br>Czoski Murray, Carolyn; University of Leeds, Leeds Institute of Health Sciences<br>Tolan, Damian; Leeds Teaching Hospitals NHS Trust<br>Stocken, Deborah; University of Leeds, Leeds Institute of Clinical Trials Research<br>Jayne, David; University of Leeds, Leeds Institute of Medical Research

Keywords:	SURGERY, GASTROENTEROLOGY, NEUROPHYSIOLOGY

SCHOLARONE™
Manuscripts

I, the Submitting Author has the right to grant and does grant on behalf of all authors of the Work (as defined in the below author licence), an exclusive licence and/or a non-exclusive licence for contributions from authors who are: i) UK Crown employees; ii) where BMJ has agreed a CC-BY licence shall apply, and/or iii) in accordance with the terms applicable for US Federal Government officers or employees acting as part of their official duties; on a worldwide, perpetual, irrevocable, royalty-free basis to BMJ Publishing Group Ltd ("BMJ") its licensees and where the relevant Journal is co-owned by BMJ to the co-owners of the Journal, to publish the Work in this journal and any other BMJ products and to exploit all rights, as set out in our licence.

The Submitting Author accepts and understands that any supply made under these terms is made by BMJ to the Submitting Author unless you are acting as an employee on behalf of your employer or a postgraduate student of an affiliated institution which is paying any applicable article publishing charge ("APC") for Open Access articles. Where the Submitting Author wishes to make the Work available on an Open Access basis (and intends to pay the relevant APC), the terms of reuse of such Open Access shall be governed by a Creative Commons licence – details of these licences and which Creative Commons licence will apply to this Work are set out in our licence referred to above.

Other than as permitted in any relevant BMJ Author's Self Archiving Policies, I confirm this Work has not been accepted for publication elsewhere, is not being considered for publication elsewhere and does not duplicate material already published. I confirm all authors consent to publication of this Work and authorise the granting of this licence.

For peer review only

Non-invasive, vagus nerve stimulation to reduce ileus after colorectal surgery: Protocol for a feasibility trial with nested mechanistic studies

Chapman SJ[1]. Naylor M[2]. Czoski Murray CJ[3]. Tolan D[4]. Stocken DD[5]. Jayne DG[6].

1. Leeds Institute of Medical Research, University of Leeds, UK; S.Chapman@leeds.ac.uk

2. West Riding of Yorkshire Ileostomy Association; m.naylor70@btinternet.com

3. Leeds Institute of Health Sciences, University of Leeds, UK; C.J.CzoskiMurray@leeds.ac.uk

4. Leeds Teaching Hospitals NHS Trust, Leeds, UK; Damian.Tolan@nhs.net

5. Leeds Institute for Clinical Trials Research, University of Leeds, UK: D.D.Stocken@leeds.ac.uk

6. Leeds Institute of Medical Research, University of Leeds, UK; D.G.Jayne@leeds.ac.uk

Corresponding Author

Stephen J Chapman, Room 7.16 Clinical Sciences Building, St. James's University Hospital, University of Leeds, LS2 9JT, United Kingdom

Email: S.Chapman@leeds.ac.uk; Twitter: @SJ_Chapman; ORCID: 0000-0003-2413-5690

Running title: Non-invasive vagus nerve stimulation to reduce ileus

Category: Protocol

Main body word count: 3,058

Keywords: ileus, colorectal surgery, vagus nerve stimulation, Feasibility RCT

For peer review only

Ethics approval: Research ethics approval was confirmed by the North East - Tyne & Wear South NHS Research Ethics Committee (19/NE/0217) on 2nd July 2019. Written, informed consent to participate will be obtained from all participants.

Availability of data and materials: A model consent form is available on request.

Competing interests: No competing interests are declared. The views expressed in this publication are those of the authors and not necessarily those of the NHS, the National Institute for Health Research, Health Education England, or the Department of Health.

Funding: This study is funded by a National Institute for Health Research Doctoral Research Fellowship (S Chapman; DRF-2018-11-049). Study devices are provided through the manufacturer's (electroCore™, Inc) Investigator Initiated Trial Programme.

Contributions: All authors conceptualised the study and contributed to its design. SJC prepared the protocol draft, which was reviewed and approved by all authors. The Clinical Trials Research Unit at University of Leeds oversees randomisation and University of Leeds is the study sponsor. DDS is the statistical guarantor and DGJ is the overall study guarantor.

Copyright: The Corresponding Author has the right to grant on behalf of all authors and does grant on behalf of all authors, a worldwide licence to the Publishers and its licensees in perpetuity, in all forms, formats and media (whether known now or created in the future), to i) publish, reproduce, distribute, display and store the Contribution, ii) translate the Contribution into other languages, create adaptations, reprints, include within collections and create summaries, extracts and/or, abstracts of the Contribution, iii) create any other derivative work(s) based on the Contribution, iv) to exploit all subsidiary rights in the Contribution, v) the inclusion of electronic links from the Contribution to third party material where-ever it may be located; and, vi) licence any third party to do any or all of the above.

Acknowledgements: None

For peer review only

Abstract

Introduction: Ileus is a common and distressing condition characterised by gut dysfunction after surgery. Whilst a number of interventions have aimed to curtail its impact on patients and healthcare systems, ileus is still an unmet challenge. Electrical stimulation of the vagus nerve is a promising new treatment due to its role in modulating the neuro-immune axis through a novel anti-inflammatory reflex. The protocol for a feasibility study of non-invasive vagus nerve stimulation (nVNS), and a programme of mechanistic and qualitative studies, is described.

Methods and Analysis: This is a participant-blinded, parallel-group, randomised, sham-controlled feasibility trial (IDEAL Stage 2b) of self-administered nVNS. One hundred and forty patients planned for elective, minimally-invasive, colorectal surgery, will be randomised to four schedules of nVNS before and after surgery. Feasibility outcomes include assessments of recruitment and attrition, adequacy of blinding, and compliance to the intervention. Clinical outcomes include bowel function and length of hospital stay. A series of mechanistic sub-studies exploring the impact of nVNS on inflammation and bowel motility will inform the design of the final stimulation schedule. Semi-structured interviews with participants will explore experiences and perceptions of the intervention, whilst interviews with patients who decline participation will explore barriers to recruitment.

Ethics and Dissemination: The protocol has been approved by the Tyne & Wear South NHS Research Ethics Committee (19/NE/0217) on 2nd July 2019. Feasibility, mechanistic, and qualitative findings will be disseminated to national and international partners through peer-reviewed publications, academic conferences, social media channels, and stakeholder engagement activities. The findings will build a case for or against progression to a definitive randomised assessment as well as informing key elements of study design.

Trial Registration: ISRCTN; ISRCTN62033341; Prospectively registered on 11th October 2019; http://www.isrctn.com/ISRCTN62033341.

For peer review only

Strengths and limitations of this study

- Building on previous evidence demonstrating the efficacy of vagus nerve stimulation to reduce ileus, this study will explore the feasibility of a non-invasive, self-administered device used by patients before and after colorectal surgery.
- Feasibility outcomes will support the development of a definitive, randomised controlled trial, including considerations of recruitment, device compliance, and sham-blinding.
- Qualitative outcomes will explore possible barriers to participation and compliance with the self-administered device before and after colorectal surgery
- The study will not explore the most appropriate outcome to be used in a definitive randomised controlled trial, but other related work done in parallel will fill this need.

For peer review only

Introduction

Ileus is a distressing condition which occurs in 10-20% of patients undergoing major abdominal surgery (1). It is characterised by painful abdominal distension, persistent vomiting, and delayed bowel function. This usually resolves 2-4 days after surgery, but in some cases, it may persist in excess of 10 days (2). The impact of ileus is extensive. For patients, it increases the length of hospital stay and increases the risk of serious postoperative complications such as pneumonia, surgical site infection, and venous thrombo-embolic events (3). For hospitals, it is economically burdensome and is associated with an increase of 71% in healthcare costs (4). Once considered to be a normal consequence of surgical recovery, the Association of Coloproctology in Great Britain & Ireland now considers ileus to be a research priority (5).

In the last 20 years, a number of interventions to prevent ileus and its clinical sequelae have been explored (6). Strategies to encourage early or sham-feeding (chewing gum) have been studied extensively but evidence for their effectiveness remains contentious (7, 8). New approaches to perioperative management (such as intravenous lidocaine) have been explored but uncertainty relating to efficacy, dose, and timing persist (9). Novel drugs such as mu-opioid receptor antagonists have shown promising results but economic and regulatory barriers have limited their uptake beyond North America. The consensus of existing literature suggests that minimally-invasive surgery and protocol-driven recovery offer the best chances of preventing ileus (10). Uncertainty exists over the pathophysiology of ileus, however, which is problematic for designing clinical interventions that are justified by strong scientific evidence.

Stimulation of the vagus nerve may represent a novel and evidence-based approach to reduce ileus after surgery. In pre-clinical models, electrical stimulation of the vagus nerve accelerates the recovery of gastrointestinal transit. This is facilitated through vagal afferents targeting the hypothalamic-pituitary-adrenal axis and vagal efferents targeting the cholinergic anti-inflammatory pathway, which attenuate inflammatory-mediated gut dysfunction (11-13). Early clinical studies have suggested that this is directly translatable to humans undergoing surgery (14, 15). Challenges exist, however, as to how vagus nerve stimulation can be done safely,

For peer review only

how its efficacy can be optimised, and how patient-acceptability can be ensured. A protocol for a randomised feasibility trial of non-invasive vagus nerve stimulation (nVNS) to reduce ileus, as well as a series of mechanistic and qualitative studies, is described.

For peer review only

Methods

Ethics & Governance

Ethical approval for the study was obtained from North East - Tyne & Wear South Research Ethics Committee (19/NE/0217) on 2nd July 2019. The study was prospectively registered on the ISRCTN registry (ISRCTN62033341; http://www.isrctn.com/ISRCTN62033341) on 11th October 2019 prior to the start of recruitment (Supplementary File 1). The study devices will be provided through the manufacturer's (electroCore™, Inc) Investigator Initiated Trial Programme. The present manuscript is reported according to the Standard Protocol Items Recommendation For Interventional Trials (SPIRIT) 2013 Checklist (16).

Aims & Objectives

The aim of this study is to determine the feasibility of performing a multicentre, phase-III, randomised-controlled trial (RCT) of nVNS to reduce ileus after colorectal surgery. The following objectives will explore the case for progression to a phase-III trial:

- To estimate the proportion of patients screened who are eligible for approach
- To estimate the number of approached patients who consent to randomisation
- To assess the adequacy of participant blinding to the allocated intervention
- To assess participant compliance to the intervention schedule
- To explore the safety of the intervention schedule
- To estimate the rate of missing outcome data, rate of withdrawal, and loss to follow up

Study Design

This study is a participant-blinded, parallel-group, randomised, sham-controlled feasibility trial (IDEAL Stage 2b) of nVNS to reduce ileus after colorectal surgery (Figure 1). One hundred and forty participants will be recruited across two study sites. Participants will be randomised to four arms (1:1:1:1) according to the following stimulation schedules:

For peer review only

- Pre-operative stimulation and post-operative stimulation
- Pre-operative sham and post-operative stimulation
- Pre-operative stimulation and post-operative sham
- Pre-operative sham and post-operative sham

The estimated trial duration is 24 months and all participants will be followed up for 30 days after the date of surgery.

Study Setting

The intervention will take place within the community (pre-operative stimulation) and in hospital (post-operative stimulation). All participants will undergo surgery at one of two large teaching hospitals (St. James's University Hospital, Leeds; and Bradford Royal Infirmary, Bradford). Both provide elective colorectal cancer services including laparoscopic surgery within a programme of enhanced recovery.

Eligibility Criteria

To be considered eligible for the trial, patients must satisfy all of the following inclusion criteria:

- Aged ≥ 18 years
- Able to provide written informed consent
- Planned to undergo elective, minimally-invasive (laparoscopic or robotic) surgery with a planned anastomosis, including one of the procedures listed in Table 1.

Presence of any of the following exclusion criteria will preclude participation in the trial:

- Severe cardiac disease (myocardial infarction in the last 12 months; heart failure with New York Heart Association Scale >2; 2nd or 3rd degree atrioventricular block, permanent atrial fibrillation/flutter, or previous ventricular tachycardia/fibrillation).
- Seizures or recurrent episodes of syncope (>1) in the last five years
- Previous transient ischaemic attack or cerebral vascular accident

For peer review only

- Previous vagotomy at any anatomical location
- Confirmed diagnosis of chronic gastrointestinal inflammatory condition
- Confirmed diagnosis of neuro-endocrine tumour
- Existing gastrointestinal stoma
- Implanted electrical stimulator device (such as cardiac pacemaker or defibrillator
- Structural abnormality of the neck anatomy which may impact on use of the device
- Belonging to a vulnerable population (such as homeless, prisoner)
- Pregnant, nursing, or thinking of becoming pregnant during the study period

Participants will not be eligible for entry into other interventional studies which aim to optimise surgical recovery or for which the intervention may impact on bowel function.

Intervention

Non-invasive vagus nerve stimulation will be self-administered by participants using the GammaCore® device (electroCore™, LLC, New Jersey, USA). This provides a transcutaneous electrical stimulation comprising of 5 x 5KHz sine-wave complexes delivered over 1ms and repeated at 25Hz (maximum voltage: 24V; maximum output current: 60mA). The surface landmark of the carotid pulse is used to guide positioning of the device over the cervical vagus nerve. Participants will self-administer the device twice daily for five days before and after surgery, with each administration lasting for 2 minutes on either side of the neck. A sham device identical in appearance will be used as a control. This produces a sub-therapeutic stimulation (0.1Hz biphasic direct current) whilst providing a perceptible stimulation sensation.

Study Outcome Measures

The following outcomes will assess the feasibility of a phase-III randomised controlled trial of nVNS to reduce ileus:

- Proportion of eligible patients identified from screening logs
- Number of eligible patients randomised (per month) and reasons for approach failure

For peer review only

- Adequacy of participant blinding according to a blinding poll (Bang Blinding Index) (17)
- Compliance to the pre- and post-operative stimulation schedules according to participant-reported diaries (maximum of 10 stimulations before and after surgery)
- Incidence of complications and serious complications within 30 days
- Proportion of missing clinical outcome data, withdrawals, and loss to follow-up

Progression will be determined using the parameters outlined in Table 2. “Progress” outcomes will build a case for progression to a phase-III trial. “Adjust” outcomes will build a case for progression after appropriate amendments have been made. “Stop” outcomes will contraindicate a case for progression, unless modifiable barriers are identified and further assessments of feasibility are undertaken. The predominance of such outcomes will be considered by the research team in the decision-making process.

Clinical outcomes will be collected to explore variability in candidate measures including: time to first flatus, time to first stool, time to first oral intake, total length of hospital stay, and GI-2 (composite outcome of passage of stool and oral tolerance) (18).

Participant timeline

The timing of study procedures, clinical assessments, and collection points are summarised in Table 3. Participants will attend a pre-intervention study visit, where a trained investigator will provide an interactive tutorial on how to self-administer the device. This will provide an opportunity to practice self-administration under direct supervision. Allocation of study devices will take place at two timepoints. The first will take place at the pre-intervention study visit, where devices will be provided for use in the community. Upon admission to hospital, these will be collected and switched with the post-operative device allocation. Throughout the stimulation schedule, participants will record their compliance to self-administering the device using stimulation diaries. On the last stimulation day, participants will complete a poll to explore the performance of blinding before and after surgery.

For peer review only

Sample Size

As a feasibility study, no formal power calculation has been performed. In this four-arm parallel-group design, 35 participants per arm will allow robust estimates of variability in continuous clinical outcomes and 70 patients randomised to pre- and post- surgery groups will allow robust estimates of recruitment parameters (19). The total sample size of 140 allows robust exploration of both clinical and recruitment-related objectives.

Recruitment

Participants will be approached with written information in the outpatient departments of St. James's University Hospital and Bradford Royal Infirmary. Research assistants will be available to discuss the study over the phone, as well as opportunities to meet in person during patients' routine schedule of pre-assessment and anaesthetic review appointments.

Randomisation & Allocation

Following confirmation of written informed consent, participants will be randomised to one of four intervention arms. The randomisation list will be prepared by an independent statistician using block randomisation with a variable block size. This will be stratified for type of colorectal resection (right- and left-sided) and recruitment centre. Randomisation will be facilitated centrally using an online 24-hour service and will be performed by study investigators.

Blinding

Participants will be blinded to the study intervention before and after surgery. This is made possible through the use of a sham stimulator device which is identical in appearance, weight, and user-interface, as well as being designed to provide a perceptible sub-therapeutic stimulation. All devices will be switched irrespective of whether the type of device (active or sham) remains constant throughout the pre- and post-operative schedules. Investigators and clinical outcome assessors will be unblinded to the intervention. Given the objective clinical outcomes, the risk of assessor bias is considered low.

For peer review only

Data Collection & Management

All trial data will be entered on to electronic case report forms by research staff. Physical data will be stored in a restricted-access research unit, accessible only to the research team. All electronic data will be stored in pseudo-anonymised format at University of Leeds according to sponsor requirements. As a feasibility study, a data monitoring committee is not required.

Statistical Analysis Plan

All analyses will follow a predetermined statistical analysis plan (20) and will be overseen by a qualified medical statistician. Recruitment, baseline characteristics, compliance and outcome data will be presented descriptively as rates (categorical) and means (continuous) with 95 per cent confidence intervals. No formal statistical hypothesis testing across randomised groups will be conducted. Groups may be collapsed to pre- and post- surgery stimulus vs sham and reported descriptively. Blinding will be analysed according to the Bang Blinding Index, with an index of >0.2 representing unblinding, -0.2< x < 0.2 representing random guesses, and < -0.2 representing opposite guessing (also considered a source of unblinding) (17). All analyses will be performed for intention-to-treat (treatment as randomised) and per-protocol populations. Possible reasons for exclusion from the per-protocol population include non-compliance to the device schedule, conversion from laparoscopic to open surgery, return to theatre prior to the return of bowel function, and randomisation errors. The trial will be reported according to CONSORT (21).

Patient and public involvement

As a self-administered device, patient and public involvement is essential at each stage of the study. The design of this feasibility trial was informed through a patient focus group in collaboration with a study-specific patient representative. A patient advisory group comprising of 4-6 patients with experience of the intervention will be convened on six occasions across three years. The programme of discussion will be determined by the group, but will include issues of patient approach, intervention burden, device training, and design of study materials.

For peer review only

Dissemination

Feasibility, mechanistic, and qualitative findings will be disseminated to professional and patient groups via peer-reviewed publications, academic conferences, social-media channels, and stakeholder engagement activities.

Trial Sub-studies

Participants will be invited to take part in the following optional sub-studies:

– *Inflammatory response sub-study*

The inflammatory response sub-study will explore the effect of nVNS on measures of intestinal and systemic inflammation. This will explore differences in efficacy between randomised arms, which will assist with the design of the final intervention. Recruitment to this sub-study will take place at the lead site only (St. James's University Hospital). Additional eligibility criteria will apply, as outlined in Table 4. Bloods draws will be performed at baseline, 2 hours, 24 hours, and 48 hours after the start of surgery. In addition, peritoneal fluid samples will be collected at the start and end of surgery. Serum (systematic) and peritoneal fluid (intestinal) levels of tumour necrosis factor (TNF) alpha, interleukin (IL) 1-beta, and IL-6 will be analysed using enzyme-linked immunosorbent assays (ELISA) (22, 23).

– *Bowel motility sub-study*

The motility sub-study will explore the effect of nVNS on global small bowel motility. Differences in efficacy between randomised groups will inform the design of the final intervention. Recruitment will take place at the lead site only. Additional eligibility criteria (Table 4) will apply with a recruitment target of 10 participants per group (total sample: 40 participants). Magnetic resonance (MR) enterography will be performed at baseline and on the third post-operative day in the supine position under breath hold conditions whilst a series of motility acquisitions are performed. These will be

For peer review only

registered with dedicated software and a quantitative motility score generated to depict bowel wall motion (24).

– *Qualitative sub-study*

A qualitative sub-study will explore participants' acceptability of the trial recruitment process, intervention, their experiences of self-administration, and any challenges with device blinding. Recruitment will take place at the lead site with a target sample of 5 participants per group (total sample: 20 participants). In addition, 5 patients who declined participation in the study will be recruited to explore barriers to study enrolment. A maximum variation approach to sampling will be used to ensure that a wide variety of perspectives are captured, account for age, sex, and indication for surgery). Semi-structured interviews will be conducted after hospital discharge in hospital or in the community. Questions will be informed by a topic guide and will be recorded in preparation for transcription. A thematic framework approach will be used for analysis, comprising five key stages: I) familiarisation with data; II) identifying the thematic framework; III) indexing; IV) charting; and V) mapping and interpreting (25). The thematic analysis will be modified in light of new data and a process of constant comparison will be used to examine across themes and cases. Qualitative data will be managed in NVivo® 11 (Melbourne, Australia).

For peer review only

Discussion

Although Ileus is a common problem after surgery, limited progress has been made to curtail its impact on patients and healthcare systems in the last 20 years (6). Perhaps the most successful measures have been the widespread introduction of minimally invasive surgery and the development of evidence-based enhanced recovery protocols. These gains are likely facilitated through reduced inflammatory- and opioid-induced intestinal smooth muscle dysfunction, leading to faster recovery of bowel function (26, 27). The use of vagus nerve stimulation to reduce ileus is a new concept which draws on similar principles. Its efficacy has been demonstrated consistently in pre-clinical studies, where stimulation of the vagus nerve has been shown to reduce intestinal inflammation and restore transit through activation of a cholinergic anti-inflammatory pathway (11). In keeping with this, intra-operative stimulation of the vagus nerve has been shown to attenuate systemic markers of inflammation in patients undergoing major abdominal surgery (14). Taken together, this suggests that the anti-inflammatory mechanism of vagus nerve stimulation is directly translatable to humans and may represent a novel approach to reduce ileus after surgery.

Most previous studies of vagus nerve stimulation have involved surgical access to the neck and abdomen. This is challenging for clinical translation because the procedure is invasive and technically demanding. nVNS aims to mitigate these challenges by facilitating non-invasive stimulation over the landmark of the cervical vagus nerve. Stimulation can be performed before and after surgery, which is attractive since some evidence suggests that activation of the cholinergic anti-inflammatory pathway is most effective when done in the pre-operative period (28). In addition, stimulation can be self-administered by patients which encourages active participation in recovery. Previous evidence has shown that active participation is a key motivator of recovery after surgery (29). On the other hand, there are several challenges associated with nVNS in this setting. Firstly, self-administration requires adequate skill, understanding, and compliance, which may be difficult in the post-operative setting. A dedicated programme of device training prior to starting the stimulation schedule

For peer review only

aims to mitigate this. Secondly, the non-invasive approach to stimulation is less precise than conventional stimulation since contact with the vagus nerve cannot be confirmed objectively. This is mitigated through the use of a standard technique, which has been shown to increase heart rate variability, reduce markers of systemic inflammation, and generate vagal somatosensory evoked potentials in healthy volunteers (i.e. indicators of vagal stimulation) previously (30-33). Providing that these challenges are addressed, nVNS may provide a practical approach to stimulating the vagus nerve in the pre- and post-operative setting.

The present study will inform the feasibility of a phase-III randomised controlled trial of nVNS by addressing key challenges in trial design. This includes compliance to the stimulation schedule, which is important in the setting of major abdominal surgery where unique barriers may exist. It also includes the adequacy of participant blinding. This is important in studies of electrical nerve stimulation, where sham-controls are often associated with statistical improvements in outcomes (34). Some issues of feasibility are beyond the scope of this study. The selection of outcomes for ileus are poorly standardised, with over 50 different outcomes identified from previous literature in the last 20 years (35). Such wide variation is challenging for evaluating new treatments and translating these into clinical practice. To address this, an international core outcome set for gastrointestinal recovery after major surgery is under development and will provide a standardised approach to outcome reporting in the future (36). In addition, the feasibility of implementing nVNS into enhanced recovery protocols across different settings is unclear. The present study explores the feasibility of nVNS in two large teaching hospitals, but further consideration of barriers to implementation will be required.

For peer review only

References

1. Scarborough JE. Schumacher J. Kent KC. Heise CP. Greenberg CC. Associations of Specific Postoperative Complications With Outcomes After Elective Colon Resection: A Procedure-Targeted Approach Toward Surgical Quality Improvement. JAMA Surg 2017;152:e164681.
2. Yorkshire Surgical Research Collaborative. Multicentre observational study of gastrointestinal recovery after elective colorectal surgery. Colorectal Dis 2018;20:536-544.
3. Tevis SE. Carchman EH. Foley EF. Harms BA. Heise CP. Kennedy GD. Postoperative Ileus-More than Just Prolonged Length of Stay? J Gastrointest Surg. 2015;19:1684-90.
4. Mao H. Milne TGE. O'Grady G. Vather R. Edlin R. Bissett I. Prolonged Postoperative Ileus Significantly Increases the Cost of Inpatient Stay for Patients Undergoing Elective Colorectal Surgery: Results of a Multivariate Analysis of Prospective Data at a Single Institution. Dis Colon Rectum. 2019;62:631-637.
5. Tiernan J, Cook A, Geh I. et al. Use of a modified Delphi approach to develop research priorities for the association of coloproctology of Great Britain and Ireland. Colorectal Dis 2014;16:965-975.
6. Chapman SJ. Pericleous A. Downey C. Jayne DG. Postoperative ileus following major colorectal surgery. Br J Surg 2018;105:797-810.
7. Herbert G. Perry R. Andersen HK. Atkinson C. Penfold C. Lewis SJ, et al. Early enteral nutrition within 24 hours of lower gastrointestinal surgery versus later commencement for length of hospital stay and postoperative complications. Cochrane Database Syst Rev 2019: CD004080.
8. Short V. Herbert G. Perry R. Atkinson C. Ness AR. Penfold C et al. Chewing gum for postoperative recovery of gastrointestinal function. Cochrane Database Syst Rev 2015: CD006506.
9. Kranke P. Jokinen J. Pace NL. Schnabel A. Hollmann MW. Hahnenkamp K. et al. Continuous intravenous perioperative lidocaine infusion for postoperative pain and recovery. Cochrane Database Syst Rev 2015 :CD009642
10. Chapman SJ. Wells CI. Challenges in ileus research. Colorectal Dis 2018;20:639.
11. Matteoli G. Gomez-Pinilla PJ. Nemethova. A. Di Giovangiulio M. Cailotto C. van Bree SH. et al. A distinct vagal anti-inflammatory pathway modulates intestinal muscularis resident macrophages independent of the spleen. Gut 2014;63:938–948.
12. de Jonge WJ. van der Zanden EP. The FO. Bijlsma MF. van Westerloo DJ. et al. Stimulation of the vagus nerve attenuates macrophage activation by activating the Jak2-STAT3 signalling pathway. Nat Immunol 2005;6:844-851.
13. Bonaz B. Sinniger V. Pellissier S. The Vagus Nerve in the Neuro-Immune Axis: Implications in the Pathology of the Gastrointestinal Tract. Front Immunol 2017;8:1452.

For peer review only

14. Stakenborg N. Wolthuis AM. Gomez-Pinilla PJ. Farro G. Di Giovangiulio M. Bosmans G. et al. Abdominal vagus nerve stimulation as a new therapeutic approach to prevent postoperative ileus. Neurogastro & Motility. 2017;29:e13075.
15. Hong G. Pintea B. Lingohr P. Coch C. Randau T. Schaefer N. et al. Effect of transcutaneous vagus nerve stimulation on muscle activity in the gastrointestinal tract (transVaGa): a prospective clinical trial. International J of Colorectal Dis 2019;34:417–422.
16. Chan AW. Tetzlaff JM. Altman DG, et al. SPIRIT 2013 statement: defining standard protocol items for clinical trials. Ann Intern Med. 2013;158:200-7.
17. Bang H. Ni L. Davis CE. Assessment of blinding in clinical trials. Control Clin Trials 2004;25:143-156.
18. van Bree SH. Bemelman WA. Hollmann MW. Zwinderman AH. Matteoli G. El Temna S. et al. Identification of clinical outcome measures for recovery of gastrointestinal motility in postoperative ileus. Ann Surg 2014;259;708-714.
19. Teare MD. Dimairo M. Shephard N. Hayman A. Whitehead A. Walters SJ. Sample size requirements to estimate key design parameters from external pilot randomised controlled trials: a simulation study. Trials 2014;15:264.
20. Gamble C. Krishan A. Stocken D. Guidelines for the Content of Statistical Analysis Plans in Clinical Trials. JAMA 2017;318:2337-2343.
21. Moher D. Hopewell S. Schulz KF. CONSORT 2010 explanation and elaboration: updated guidelines for reporting parallel group randomised trials. BMJ 2010;340:c869
22. Lange T. Dimitrov S. Born J. Effect of sleep and circadian rhythm on the human immune system. Ann N Y Acad Sci 210;1193:48-59.
23. Baigrie J. Lamont PM. Kwiatkowski D. Dallman MJ. Morris PJ. Systemic cytokine response after major surgery. Br J Surgery 1992;79:757-60.
24. Menys A. Taylor SA. Emmanuel A. Ahmed A. Plumb AA. Odille F. Alam A. Halligan S. Atkinson D. Global small bowel motility: assessment with dynamic MR imaging. Radiology. 2013;269:443-50.
25. Ritchie J. Lewis J. McNaughton Nicholls C. Ormston R. Qualitative Research Practice: A Guide for Social Science Students and Researchers 2nd Ed. Sage. London. 2014
26. Schwenk W. Jacobi C. Mansmann U. Böhm B. Müller JM. Inflammatory response after laparoscopic and conventional colorectal resections - results of a prospective randomized trial. Langenbecks Arch Surg 2000;385:2-9.
27. Venara A. Duchalais E. Dariel A. Aubert P. Durand T. Meurette G. Rolli-Derkinderen M. Hamy A. Neunlist M. Anti-inflammatory Effects of Enhanced Recovery Programs on Early-Stage Colorectal Cancer Surgery. World J Surg 2018;42:953-964.

For peer review only

28. Stakenborg N. Labeeuw E. Gomez-Pinilla PJ. De Schepper S. Aerts R. et al. Preoperative administration of the 5-HT4 receptor agonist prucalopride reduces intestinal inflammation and shortens postoperative ileus via cholinergic enteric neurons. Gut 2019;68:1406-1416.
29. Chapman SJ. Czoski Murray CJ. Lonsdale MDS. Boyes S. Tiernan JP. et al. Information needs for recovery after colorectal surgery: Patient focus group study. *Colorectal Dis* 2020 (In Press).
30. Brock C. Brock B. Aziz Q. Møller HJ. Pfeiffer Jensen M. Drewes AM. Farmer AD. Transcutaneous cervical vagal nerve stimulation modulates cardiac vagal tone and tumor necrosis factor-alpha. Neurogastroenterol Motil 2017;29. doi: 10.1111/nmo.12999.
31. Lerman I. Hauger R. Sorkin L. Proudfoot J. Davis B. et al. Noninvasive Transcutaneous Vagus Nerve Stimulation Decreases Whole Blood Culture-Derived Cytokines and Chemokines: A Randomized, Blinded, Healthy Control Pilot Trial. Neuromodulation 2016;19:283-90.
32. Vecchio E. Bassez I. Ricci K. Tassorelli C. Liebler E. et al. Effect of Non-invasive Vagus Nerve Stimulation on Resting-State Electroencephalography and Laser-Evoked Potentials in Migraine Patients: Mechanistic Insights. Front Hum Neurosci 2018;13;12:366.
33. Nonis R. D'Ostilio K. Schoenen J. Magis D. Evidence of activation of vagal afferents by non-invasive vagus nerve stimulation: An electrophysiological study in healthy volunteers. *Cephalgia* 2017;37:1285-1293.
34. Tan K. Wells CI. Dinning P. Bissett IP. O'Grady G. Placebo Response Rates in Electrical Nerve Stimulation Trials for Fecal Incontinence and Constipation: A Systematic Review and Meta-Analysis. Neuromodulation 2019. doi: 10.1111/ner.13092. [Epub ahead of print]
35. Chapman SJ. Thorpe G. Vallance AE. Harji DP. Lee MJ. et al. Systematic review of definitions and outcome measures for return of bowel function after gastrointestinal surgery. BJS Open 2018;3:1-10.
36. Chapman SJ. Lee MJ. Blackwell S. Arnott R. Ten Broek RPG. et al. Establishing core outcome sets for gastrointestinal recovery in studies of postoperative ileus and small bowel obstruction: protocol for a nested methodological study. Colorectal Dis. 2019. doi: 10.1111/codi.14899. [Epub ahead of print]

For peer review only

Table 1 – Eligible Procedures

**Right sided resection**	**Left sided resection**
Ileocolic/caecal resection	Extended left hemicolectomy
Right hemicolectomy	Left hemicolectomy
Extended right hemicolectomy	Sigmoid colectomy
Transverse colectomy	Rectosigmoid colectomy
**Miscellaneous (side grouped accordingly)**	Anterior resection
Other segmental colonic resection	

For peer review only

Table 2 – Progression criteria

Criteria	Stop	Adjust	Progress
Proportion of eligible patients identified from screening logs	<10%	10-20%	>20%
Number of eligible patients randomised over 24 months (site: SJUH)	≤2 per month	3-4 per month	≥5 per month
Number of eligible patients randomised over 18 months (site: BRI)	<1 per month	1-2 per month	≥3 per month
Adequacy of participant blinding (according to Bang Blinding Index)	Index < -0.5<br>Or Index > 0.5	Index -0.2 to -0.5<br>Or Index 0.2 to 0.5	Index 0 to -0.19<br>Or Index 0 to 0.19
Average compliance to the study treatment schedule *	<10/20 stimulations across 10 days	10-15/20 stimulations across 10 days	≥16/20 stimulations across 10 days
Proportion of missing clinical outcome data	>40%	15-30%	<15%
Proportion of randomised patients lost to follow up	>40%	15-30%	<15%
Incidence of complications or serious complications	>20% increase in complications	5-20% increase in complications	<5% increase in complications

BRI: Bradford Royal Infirmary, Bradford; SJUH: St. James's University Hospital, Leeds

For peer review only

Table 3 – Schedule of Events

	Events	Baseline	Preop study visit	5 days before surgery	Day of surgery (pre-op)	Day of surgery (Intraop)	POD 1	POD 2	POD 3	POD 4	POD 5	POD 6-10	After discharge	30-day phone F/U
**Study Procedures**	Trial consent	✓												
	Device training		✓											
	Device distribution		✓		✓									
	Self-administered stimulation			✓			✓	✓	✓	✓	✓			
	Stimulation diary			✓			✓	✓	✓	✓	✓			
	Blinding poll										✓			
**Data collection**	Eligibility CRF	✓												
	Baseline CRF	✓												
	Operative CRF					✓								
	Gastrointestinal Function CRF						✓	✓	✓	✓	✓	✓		
	Follow-up CRF													✓
**Sub-studies***	*Optional sub-study consents*	✓												
	Inflammatory response study<br>Blood only: ✓; Blood and fluid ✓✓				✓	✓✓	✓		✓					
	Small bowel motility study (MRI)		✓						✓					
	Qualitative sub-study (participant or non-participant interview)												✓	

**Sub-studies are optional and undertaken at the lead site only*
CRF: Case Report Form; F/U: follow up; MRI: magnetic resonance imaging; POD: postoperative day

For peer review only

Table 4 – Sub-study additional exclusion criteria

**Inflammatory response sub-study**
• Regular use of non-steroidal anti-inflammatory drugs in the preceding 7 days<br>• Medical condition requiring oral or injectable steroids<br>• Neoadjuvant chemoradiotherapy within 12 months prior to surgery
**Bowel motility sub-study**
• Indwelling pacemaker or cardiac defibrillator device<br>• Non-MR compatible metallic implants, prostheses, neurosurgical clips, indwelling stimulator or pump devices, which would preclude safe MRI<br>• Claustrophobia<br>• Metallic foreign bodies in the eyes

MRI: Magnetic Resonance Imaging

For peer review only

Figure 1 – Study flow chart

For peer review only

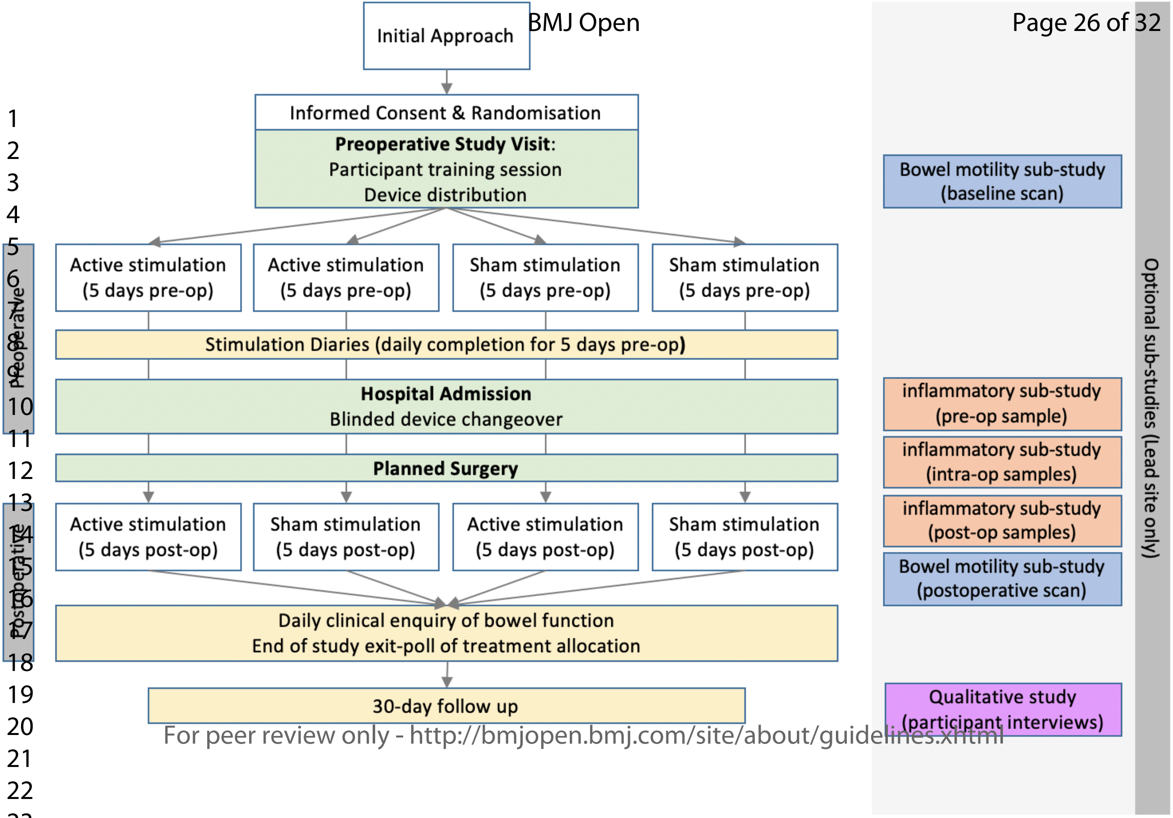

For peer review only - http://bmjopen.bmj.com/site/about/guidelines.xhtml

Supplementary File

Study Status

The study first began recruitment on 2nd January 2020 and is expected to complete by 31st August 2021. The current protocol version is v1.2 (13th February 2020).

Auditing

The conduct of the trial will be audited monthly by the principal investigator. In line with Good Clinical Practice, a study site folder will be held at each participating recruitment site. This will contain the current study protocol, delegation log outlining responsibilities of all research team members, and all study correspondence. Data relating to recruitment will be uploaded to the NIHR Central Portfolio Management System on a monthly basis.

Adverse Events

The reporting of adverse events will be done in line with Good Clinical Practice. All serious adverse events will be reported to the sponsor within 24 hours of research staff becoming aware of the event. Serious adverse events that are considered to be unexpected and related to the study will be escalated to the research ethics committee.

Study Amendments

If amendments to the study protocol or documents are required, this will be done and communicated in line with Health Research Authority guidelines. All amendments will be categorised and submitted for review by the Research Ethics Committee, as appropriate.

Dissemination Plan

The findings of the study and sub-studies will be disseminated at academic conferences and in peer-reviewed manuscripts. The results will also be summarised in plain English visual abstracts and disseminated to research participants and the public.

For peer review only

Study Sponsor

The sponsor for this study is University of Leeds

Recruitment & Protocol Materials

Study recruitment and protocol materials are available upon request from the corresponding author.

For peer review only

SPIRIT

STANDARD PROTOCOL ITEMS: RECOMMENDATIONS FOR INTERVENTIONAL TRIALS

SPIRIT 2013 Checklist: Recommended items to address in a clinical trial protocol and related documents*

Section/item	Item No	Description
**Administrative information**		
Title	1	Descriptive title identifying the study design, population, interventions, and, if applicable, trial acronym **(Page 1)**
Trial registration	2a	Trial identifier and registry name. If not yet registered, name of intended registry **(Page 5)**
	2b	All items from the World Health Organization Trial Registration Data Set **(Page 5)**
Protocol version	3	Date and version identifier **(Page 5)**
Funding	4	Sources and types of financial, material, and other support **(Page 1)**
Roles and responsibilities	5a	Names, affiliations, and roles of protocol contributors **(Page 1)**
	5b	Name and contact information for the trial sponsor **(Suppl. File 1)**
	5c	Role of study sponsor and funders, if any, in study design; collection, management, analysis, and interpretation of data; writing of the report; and the decision to submit the report for publication, including whether they will have ultimate authority over any of these activities **(Page 1)**
	5d	Composition, roles, and responsibilities of the coordinating centre, steering committee, endpoint adjudication committee, data management team, and other individuals overseeing the trial, if applicable (see Item 21a for data monitoring committee) **(N/A)**
**Introduction**		
Background and rationale	6a	Description of research question and justification for undertaking the trial, including summary of relevant studies (published and unpublished) examining benefits and harms for each intervention **(Page 3-4)**
	6b	Explanation for choice of comparators **(Page 3-4)**
Objectives	7	Specific objectives or hypotheses **(Page 5)**

For peer review only

Trial design	8	Description of trial design including type of trial (eg, parallel group, crossover, factorial, single group), allocation ratio, and framework (eg, superiority, equivalence, noninferiority, exploratory) **(Page 5)**

Methods: Participants, interventions, and outcomes

Study setting	9	Description of study settings (eg, community clinic, academic hospital) and list of countries where data will be collected. Reference to where list of study sites can be obtained **(Page 6)**
Eligibility criteria	10	Inclusion and exclusion criteria for participants. If applicable, eligibility criteria for study centres and individuals who will perform the interventions (eg, surgeons, psychotherapists) **(Page 6)**
Interventions	11a	Interventions for each group with sufficient detail to allow replication, including how and when they will be administered **(Page 7)**
	11b	Criteria for discontinuing or modifying allocated interventions for a given trial participant (eg, drug dose change in response to harms, participant request, or improving/worsening disease) **(N/A)**
	11c	Strategies to improve adherence to intervention protocols, and any procedures for monitoring adherence (eg, drug tablet return, laboratory tests) **(N/A)**
	11d	Relevant concomitant care and interventions that are permitted or prohibited during the trial **(Page 7)**
Outcomes	12	Primary, secondary, and other outcomes, including the specific measurement variable (eg, systolic blood pressure), analysis metric (eg, change from baseline, final value, time to event), method of aggregation (eg, median, proportion), and time point for each outcome. Explanation of the clinical relevance of chosen efficacy and harm outcomes is strongly recommended **(Page 7-8)**
Participant timeline	13	Time schedule of enrolment, interventions (including any run-ins and washouts), assessments, and visits for participants. A schematic diagram is highly recommended (see Figure) **(Page 8-9)**
Sample size	14	Estimated number of participants needed to achieve study objectives and how it was determined, including clinical and statistical assumptions supporting any sample size calculations **(Page 9)**
Recruitment	15	Strategies for achieving adequate participant enrolment to reach target sample size **(Page 9)**

For peer review only - http://bmjopen.bmj.com/site/about/guidelines.xhtml

Methods: Assignment of interventions (for controlled trials)

Allocation:

Sequence generation	16a	Method of generating the allocation sequence (eg, computer-generated random numbers), and list of any factors for stratification. To reduce predictability of a random sequence, details of any planned restriction (eg, blocking) should be provided in a separate document that is unavailable to those who enrol participants or assign interventions **(Page 9)**
Allocation concealment mechanism	16b	Mechanism of implementing the allocation sequence (eg, central telephone; sequentially numbered, opaque, sealed envelopes), describing any steps to conceal the sequence until interventions are assigned **(Page 9)**
Implementation	16c	Who will generate the allocation sequence, who will enrol participants, and who will assign participants to interventions **(Page 9)**
Blinding (masking)	17a	Who will be blinded after assignment to interventions (eg, trial participants, care providers, outcome assessors, data analysts), and how **(Page 9-10)**
	17b	If blinded, circumstances under which unblinding is permissible, and procedure for revealing a participant's allocated intervention during the trial **(N/A)**

Methods: Data collection, management, and analysis

Data collection methods	18a	Plans for assessment and collection of outcome, baseline, and other trial data, including any related processes to promote data quality (eg, duplicate measurements, training of assessors) and a description of study instruments (eg, questionnaires, laboratory tests) along with their reliability and validity, if known. Reference to where data collection forms can be found, if not in the protocol **(Page 10)**
	18b	Plans to promote participant retention and complete follow-up, including list of any outcome data to be collected for participants who discontinue or deviate from intervention protocols
Data management	19	Plans for data entry, coding, security, and storage, including any related processes to promote data quality (eg, double data entry; range checks for data values). Reference to where details of data management procedures can be found, if not in protocol **(Page 10)**
Statistical methods	20a	Statistical methods for analysing primary and secondary outcomes. Reference to where other details of the statistical analysis plan can be found, if not in the protocol **(Page 10)**
	20b	Methods for any additional analyses (eg, subgroup and adjusted analyses) **(Page 10)**

	20c	Definition of analysis population relating to protocol non-adherence (eg, as randomised analysis), and any statistical methods to handle missing data (eg, multiple imputation) **(Page 10)**
**Methods: Monitoring**		
Data monitoring	21a	Composition of data monitoring committee (DMC); summary of its role and reporting structure; statement of whether it is independent from the sponsor and competing interests; and reference to where further details about its charter can be found, if not in the protocol. Alternatively, explanation of why a DMC is not needed **(Suppl. File 1)**
	21b	Description of any interim analyses and stopping guidelines, including who will have access to these interim results and make the final decision to terminate the trial **(Suppl. File 1)**
Harms	22	Plans for collecting, assessing, reporting, and managing solicited and spontaneously reported adverse events and other unintended effects of trial interventions or trial conduct **(Suppl. File 1)**
Auditing	23	Frequency and procedures for auditing trial conduct, if any, and whether the process will be independent from investigators and the sponsor **(Suppl. File 1)**
**Ethics and dissemination**		
Research ethics approval	24	Plans for seeking research ethics committee/institutional review board (REC/IRB) approval **(Page 5)**
Protocol amendments	25	Plans for communicating important protocol modifications (eg, changes to eligibility criteria, outcomes, analyses) to relevant parties (eg, investigators, REC/IRBs, trial participants, trial registries, journals, regulators) **(Suppl. File 1)**
Consent or assent	26a	Who will obtain informed consent or assent from potential trial participants or authorised surrogates, and how (see Item 32) **(Page 9)**
	26b	Additional consent provisions for collection and use of participant data and biological specimens in ancillary studies **(Page 11-12)**
Confidentiality	27	How personal information about potential and enrolled participants will be collected, shared, and maintained in order to protect confidentiality before, during, and after the trial **(Page 10)**
Declaration of interests	28	Financial and other competing interests for principal investigators for the overall trial and each study site **(Page 1)**
Access to data	29	Statement of who will have access to the final trial dataset, and disclosure of contractual agreements that limit such access for investigators **(N/A)**
Ancillary and post-trial care	30	Provisions, if any, for ancillary and post-trial care, and for compensation to those who suffer harm from trial participation **(N/A)**

For peer review only - http://bmjopen.bmj.com/site/about/guidelines.xhtml

Dissemination policy	31a	Plans for investigators and sponsor to communicate trial results to participants, healthcare professionals, the public, and other relevant groups (eg, via publication, reporting in results databases, or other data sharing arrangements), including any publication restrictions **(Suppl. File 1)**
	31b	Authorship eligibility guidelines and any intended use of professional writers **(Suppl. File 1)**
	31c	Plans, if any, for granting public access to the full protocol, participant-level dataset, and statistical code **(Suppl. File 1)**
**Appendices**		
Informed consent materials	32	Model consent form and other related documentation given to participants and authorised surrogates **(Suppl. File 1)**
Biological specimens	33	Plans for collection, laboratory evaluation, and storage of biological specimens for genetic or molecular analysis in the current trial and for future use in ancillary studies, if applicable **(Pages 11-12)**

*It is strongly recommended that this checklist be read in conjunction with the SPIRIT 2013 Explanation & Elaboration for important clarification on the items. Amendments to the protocol should be tracked and dated. The SPIRIT checklist is copyrighted by the SPIRIT Group under the Creative Commons "Attribution-NonCommercial-NoDerivs 3.0 Unported" license.

For peer review only

BMJ Open

Non-invasive, vagus nerve stimulation to reduce ileus after colorectal surgery: Protocol for a feasibility trial with nested mechanistic studies

Journal:	*BMJ Open*
Manuscript ID	bmjopen-2020-046313.R1
Article Type:	Protocol
Date Submitted by the Author:	01-Jan-2021
Complete List of Authors:	Chapman, Stephen; University of Leeds, Leeds Institute of Medical Research<br>Naylor, Maureen; West Riding of Yorkshire Ileostomy Association<br>Czoski Murray, Carolyn; University of Leeds, Leeds Institute of Health Sciences<br>Tolan, Damian; Leeds Teaching Hospitals NHS Trust<br>Stocken, Deborah; University of Leeds, Leeds Institute of Clinical Trials Research<br>Jayne, David; University of Leeds, Leeds Institute of Medical Research
**Primary Subject Heading**:	Surgery
Secondary Subject Heading:	Gastroenterology and hepatology, Qualitative research, Research methods
Keywords:	SURGERY, GASTROENTEROLOGY, NEUROPHYSIOLOGY

SCHOLARONE™
Manuscripts

I, the Submitting Author has the right to grant and does grant on behalf of all authors of the Work (as defined in the below author licence), an exclusive licence and/or a non-exclusive licence for contributions from authors who are: i) UK Crown employees; ii) where BMJ has agreed a CC-BY licence shall apply, and/or iii) in accordance with the terms applicable for US Federal Government officers or employees acting as part of their official duties; on a worldwide, perpetual, irrevocable, royalty-free basis to BMJ Publishing Group Ltd ("BMJ") its licensees and where the relevant Journal is co-owned by BMJ to the co-owners of the Journal, to publish the Work in this journal and any other BMJ products and to exploit all rights, as set out in our licence.

The Submitting Author accepts and understands that any supply made under these terms is made by BMJ to the Submitting Author unless you are acting as an employee on behalf of your employer or a postgraduate student of an affiliated institution which is paying any applicable article publishing charge ("APC") for Open Access articles. Where the Submitting Author wishes to make the Work available on an Open Access basis (and intends to pay the relevant APC), the terms of reuse of such Open Access shall be governed by a Creative Commons licence – details of these licences and which Creative Commons licence will apply to this Work are set out in our licence referred to above.

Other than as permitted in any relevant BMJ Author's Self Archiving Policies, I confirm this Work has not been accepted for publication elsewhere, is not being considered for publication elsewhere and does not duplicate material already published. I confirm all authors consent to publication of this Work and authorise the granting of this licence.

For peer review only

Non-invasive, vagus nerve stimulation to reduce ileus after colorectal surgery: Protocol for a feasibility trial with nested mechanistic studies

Stephen J Chapman[1]. Maureen Naylor [2]. Carolyn J Czoski Murray[3]. Damian Tolan[4]. Deborah D Stocken[5]. David Jayne[6].

1. Leeds Institute of Medical Research, University of Leeds, UK; S.Chapman@leeds.ac.uk
2. West Riding of Yorkshire Ileostomy Association; m.naylor70@btinternet.com
3. Leeds Institute of Health Sciences, University of Leeds, UK; C.J.CzoskiMurray@leeds.ac.uk
4. Leeds Teaching Hospitals NHS Trust, Leeds, UK; Damian.Tolan@nhs.net
5. Leeds Institute for Clinical Trials Research, University of Leeds, UK: D.D.Stocken@leeds.ac.uk
6. Leeds Institute of Medical Research, University of Leeds, UK; D.G.Jayne@leeds.ac.uk

Corresponding Author

Stephen J Chapman, Room 7.16 Clinical Sciences Building, St. James's University Hospital, University of Leeds, LS2 9JT, United Kingdom

Email: S.Chapman@leeds.ac.uk; Twitter: @SJ_Chapman; ORCID: 0000-0003-2413-5690

Running title: Non-invasive vagus nerve stimulation to reduce ileus

Category: Protocol

Main body word count: 3,333

Keywords: ileus, colorectal surgery, vagus nerve stimulation, Feasibility RCT

For peer review only

Abstract

Introduction: Ileus is a common and distressing condition characterised by gut dysfunction after surgery. Whilst a number of interventions have aimed to curtail its impact on patients and healthcare systems, ileus is still an unmet challenge. Electrical stimulation of the vagus nerve is a promising new treatment due to its role in modulating the neuro-immune axis through a novel anti-inflammatory reflex. The protocol for a feasibility study of non-invasive vagus nerve stimulation (nVNS), and a programme of mechanistic and qualitative studies, is described.

Methods and Analysis: This is a participant-blinded, parallel-group, randomised, sham-controlled feasibility trial (IDEAL Stage 2b) of self-administered nVNS. One hundred and forty patients planned for elective, minimally-invasive, colorectal surgery, will be randomised to four schedules of nVNS before and after surgery. Feasibility outcomes include assessments of recruitment and attrition, adequacy of blinding, and compliance to the intervention. Clinical outcomes include bowel function and length of hospital stay. A series of mechanistic sub-studies exploring the impact of nVNS on inflammation and bowel motility will inform the design of the final stimulation schedule. Semi-structured interviews with participants will explore experiences and perceptions of the intervention, whilst interviews with patients who decline participation will explore barriers to recruitment.

Ethics and Dissemination: The protocol has been approved by the Tyne & Wear South NHS Research Ethics Committee (19/NE/0217) on 2nd July 2019. Feasibility, mechanistic, and qualitative findings will be disseminated to national and international partners through peer-reviewed publications, academic conferences, social media channels, and stakeholder engagement activities. The findings will build a case for or against progression to a definitive randomised assessment as well as informing key elements of study design.

Trial Registration: ISRCTN; ISRCTN62033341; Prospectively registered on 11th October 2019; http://www.isrctn.com/ISRCTN62033341.

For peer review only

Strengths and limitations of this study

- Feasibility outcomes will support the development of a definitive, randomised controlled trial, including considerations of recruitment, device compliance, and sham-blinding.
- Qualitative outcomes will explore possible barriers to participation and compliance with the self-administered device before and after colorectal surgery
- The study will not explore the most appropriate outcome to be used in a definitive randomised controlled trial, but other related work done in parallel will fill this need.

For peer review only

Introduction

Ileus is a distressing condition which occurs in 10-20% of patients undergoing major abdominal surgery (1). It is characterised by painful abdominal distension, persistent vomiting, and delayed bowel function. This usually resolves 2-4 days after surgery, but in some cases, it may persist in excess of 10 days (2). The impact of ileus is extensive. For patients, it increases the length of hospital stay and increases the risk of serious postoperative complications such as pneumonia, surgical site infection, and venous thrombo-embolic events (3). For hospitals, it is economically burdensome and is associated with an increase of 71% in healthcare costs (4). Once considered to be a normal consequence of surgical recovery, the Association of Coloproctology in Great Britain & Ireland now considers ileus to be a research priority (5).

In the last 20 years, a number of interventions to prevent ileus and its clinical sequelae have been explored (6). Strategies to encourage early or sham-feeding (chewing gum) have been studied extensively but evidence for their effectiveness remains contentious (7, 8). New approaches to perioperative management (such as intravenous lidocaine) have been explored but uncertainty relating to efficacy, dose, and timing persist (9). Novel drugs such as mu-opioid receptor antagonists have shown promising results but economic and regulatory barriers have limited their uptake beyond North America. The consensus of existing literature suggests that minimally-invasive surgery and protocol-driven recovery offer the best chances of preventing ileus (10). Uncertainty exists over the pathophysiology of ileus, however, which is problematic for designing clinical interventions that are justified by strong scientific evidence.

Stimulation of the vagus nerve may represent a novel and evidence-based approach to reduce ileus after surgery. In pre-clinical models, electrical stimulation of the vagus nerve accelerates the recovery of gastrointestinal transit. This is facilitated through vagal afferents targeting the hypothalamic-pituitary-adrenal axis and vagal efferents targeting the cholinergic anti-inflammatory pathway, which attenuate inflammatory-mediated gut dysfunction (11-13). Early clinical studies have suggested that this is directly translatable to humans undergoing surgery (14, 15). Challenges exist, however, as to how vagus nerve stimulation can be done safely,

For peer review only

how its efficacy can be optimised, and how patient-acceptability can be ensured. A protocol for a randomised feasibility trial of non-invasive vagus nerve stimulation (nVNS) to reduce ileus, as well as a series of mechanistic and qualitative studies, is described.

For peer review only

Methods

Ethics & Governance

Ethical approval for the study was obtained from North East - Tyne & Wear South Research Ethics Committee (19/NE/0217) on 2nd July 2019. The study was prospectively registered on the ISRCTN registry (ISRCTN62033341; http://www.isrctn.com/ISRCTN62033341) on 11th October 2019 prior to the start of recruitment (Supplementary File 1). The study devices will be provided through the manufacturer's (electroCore™, Inc) Investigator Initiated Trial Programme. The present manuscript is reported according to the Standard Protocol Items Recommendation For Interventional Trials (SPIRIT) 2013 Checklist (16).

Aims & Objectives

The aim of this study is to determine the feasibility of performing a multicentre, phase-III, randomised-controlled trial (RCT) of nVNS to reduce ileus after colorectal surgery. The following objectives will explore the case for progression to a phase-III trial:

- To estimate the proportion of patients screened who are eligible for approach
- To estimate the number of approached patients who consent to randomisation
- To assess the adequacy of participant blinding to the allocated intervention
- To assess participant compliance to the intervention schedule
- To explore the safety of the intervention schedule
- To estimate the rate of missing outcome data, rate of withdrawal, and loss to follow up

Study Design

This study is a participant-blinded, parallel-group, randomised, sham-controlled feasibility trial (IDEAL Stage 2b) of nVNS to reduce ileus after colorectal surgery (Figure 1). One hundred and forty participants will be recruited across two study sites. Participants will be randomised to four arms (1:1:1:1) according to the following stimulation schedules:

For peer review only

- Pre-operative stimulation and post-operative stimulation
- Pre-operative sham and post-operative stimulation
- Pre-operative stimulation and post-operative sham
- Pre-operative sham and post-operative sham

The estimated trial duration is 24 months and all participants will be followed up for 30 days after the date of surgery.

Study Setting

The intervention will take place within the community (pre-operative stimulation) and in hospital (post-operative stimulation). All participants will undergo surgery at one of two large teaching hospitals (St. James's University Hospital, Leeds; and Bradford Royal Infirmary, Bradford). Both provide elective colorectal cancer services including laparoscopic surgery within a programme of enhanced recovery.

Eligibility Criteria

To be considered eligible for the trial, patients must satisfy all of the following inclusion criteria:

- Aged ≥ 18 years
- Able to provide written informed consent
- Planned to undergo elective, minimally-invasive (laparoscopic or robotic) surgery with a planned anastomosis/no routine plans for a diverting stoma, including one of the procedures listed in Table 1.

Presence of any of the following exclusion criteria will preclude participation in the trial:

- Severe cardiac disease (myocardial infarction in the last 12 months; heart failure with New York Heart Association Scale >2; 2nd or 3rd degree atrioventricular block, permanent atrial fibrillation/flutter, or previous ventricular tachycardia/fibrillation).
- Seizures or recurrent episodes of syncope (>1) in the last five years

For peer review only

- Previous transient ischaemic attack or cerebral vascular accident
- Previous vagotomy at any anatomical location
- Confirmed diagnosis of chronic gastrointestinal inflammatory condition
- Confirmed diagnosis of neuro-endocrine tumour
- Existing gastrointestinal stoma
- Implanted electrical stimulator device (such as cardiac pacemaker or defibrillator
- Structural abnormality of the neck anatomy which may impact on use of the device
- Belonging to a vulnerable population (such as those lacking capacity, prisoners)
- Pregnant, nursing, or thinking of becoming pregnant during the study period

Participants will not be eligible for entry into other interventional studies which aim to optimise surgical recovery or for which the intervention may impact on bowel function.

Intervention

Non-invasive vagus nerve stimulation will be self-administered by participants using the GammaCore® device (electroCore™, LLC, New Jersey, USA). This provides a transcutaneous electrical stimulation comprising of 5 x 5KHz sine-wave complexes delivered over 1ms and repeated at 25Hz (maximum voltage: 24V; maximum output current: 60mA). The surface landmark of the carotid pulse is used to guide positioning of the device over the cervical vagus nerve. Participants will self-administer the device twice daily for five days before and after surgery, with each administration lasting for 2 minutes on either side of the neck. Participants will be instructed to adjust the stimulation setting to the maximum tolerated level. A sham device identical in appearance will be used as a control. This produces a sub-therapeutic stimulation (0.1Hz biphasic direct current) whilst providing a perceptible stimulation sensation.

For peer review only

Study Outcome Measures

The following outcomes will assess the feasibility of a phase-III randomised controlled trial of nVNS to reduce ileus:

- Proportion of eligible patients identified from screening logs
- Number of eligible patients randomised (per month) and reasons for approach failure
- Adequacy of participant blinding according to a blinding poll (Bang Blinding Index) (17)
- Compliance to the pre- and post-operative stimulation schedules according to participant-reported diaries (maximum of 10 stimulations before and after surgery)
- Incidence of complications and serious complications within 30 days
- Proportion of missing clinical outcome data, withdrawals, and loss to follow-up

Progression will be determined using the parameters outlined in Table 2. “Progress” outcomes will build a case for progression to a phase-III trial. “Adjust” outcomes will build a case for progression after appropriate amendments have been made. “Stop” outcomes will contraindicate a case for progression, unless modifiable barriers are identified and further assessments of feasibility are undertaken. The predominance of such outcomes will be considered by the research team in the decision-making process.

Clinical outcomes will be collected to explore variability in candidate measures including: number of postoperative days to first flatus, first stool, first oral intake, hospital discharge, and GI-2 (composite outcome of passage of stool and oral tolerance) (18).

Participant timeline

The timing of study procedures, clinical assessments, and collection points are summarised in Table 3. Participants will attend a pre-intervention study visit, where a trained investigator will provide an interactive tutorial on how to self-administer the device. This will provide an opportunity to practice self-administration under direct supervision. Allocation of study devices will take place at two timepoints. The first will take place at the pre-intervention study visit,

For peer review only

where devices will be provided for use in the community. Upon admission to hospital, these will be collected and switched with the post-operative device. Throughout the stimulation schedule, participants will record their compliance to the device schedule using stimulation diaries. Gastrointestinal function will be recorded daily, as reported by patients. On the last stimulation day, participants will complete a poll to explore the performance of blinding before and after surgery. Follow up data (including the occurrence of complications and serious complications) will be collected 30 days after surgery to capture events which may occur during the index hospital admission or after discharge.

Sample Size

As a feasibility study, no formal power calculation has been performed. In this four-arm parallel-group design, 35 participants per arm will allow robust estimates of variability in continuous clinical outcomes and 70 patients randomised to pre- and post- surgery groups will allow robust estimates of recruitment parameters (19). The total sample size of 140 allows robust exploration of both clinical and recruitment-related objectives.

Recruitment

Participants will be approached with written information in the outpatient departments of St. James's University Hospital and Bradford Royal Infirmary. Research staff will be available to discuss the study over the phone, as well as opportunities to meet in person during patients' routine schedule of pre-assessment and anaesthetic review appointments.

Randomisation & Allocation

Following confirmation of written informed consent, participants will be randomised to one of four intervention arms. The randomisation list will be prepared by an independent statistician using block randomisation with a variable block size. This will be stratified for type of colorectal resection (right- and left-sided) and recruitment centre. Randomisation will be facilitated centrally using an online 24-hour service and will be performed by study investigators.

For peer review only

Blinding

Participants will be blinded to the study intervention before and after surgery. This is made possible through the use of a sham stimulator device which is identical in appearance, weight, and user-interface, as well as being designed to provide a perceptible sub-therapeutic stimulation. All devices will be switched irrespective of whether the type of device (active or sham) remains constant throughout the pre- and post-operative schedules. Investigators and clinical outcome assessors will be unblinded to the intervention. Since outcomes relating to gastrointestinal function are patient-reported, the risk of assessor bias is considered to be low.

Data Collection & Management

All trial data will be prospectively entered on to case report forms by dedicated research staff (a clinical research fellow and research nurses). Physical data will be stored in a restricted-access research unit, accessible only to the research team. All electronic data will be stored in pseudo-anonymised format at University of Leeds according to sponsor requirements. As a feasibility study, a data monitoring committee is not required.

Statistical Analysis Plan

All analyses will follow a predetermined statistical analysis plan (20) and will be overseen by a qualified medical statistician. Recruitment, baseline characteristics, compliance and outcome data will be presented descriptively as rates (categorical) and means (continuous) with 95 per cent confidence intervals. No formal statistical hypothesis testing across randomised groups will be conducted. Groups may be collapsed to pre- and post- surgery stimulus vs sham and reported descriptively. Blinding will be analysed according to the Bang Blinding Index, with an index of >0.2 representing unblinding, -0.2< x < 0.2 representing random guesses, and < -0.2 representing opposite guessing (also considered a source of unblinding) (17). All analyses will be performed for intention-to-treat (treatment as randomised) and per-protocol populations. Possible reasons for exclusion from the per-protocol population include non-compliance to the device schedule (defined as any deviation from the five-day

For peer review only

pre- and post-operative stimulation schedule), conversion from laparoscopic to open surgery, formation of unplanned stoma, return to theatre prior to the return of bowel function, and randomisation errors. The trial will be reported according to CONSORT (21).

Patient and public involvement

As a self-administered device, patient and public involvement is essential at each stage of the study. The design of this feasibility trial was informed through a patient focus group in collaboration with a study-specific patient representative. A patient advisory group comprising of 4-6 patients with experience of the intervention will be convened on six occasions across three years (including one year prior to study recruitment and two years during recruitment). The programme of discussion will be determined by the group, but will include issues of patient approach, intervention burden, device training, and design of study materials.

Dissemination

Feasibility, mechanistic, and qualitative findings will be disseminated to professional and patient groups via peer-reviewed publications, academic conferences, social-media channels, and stakeholder engagement activities.

Trial Sub-studies

Participants will be invited to take part in the following optional sub-studies:

- *Inflammatory response sub-study*

  The inflammatory response sub-study will explore the effect of nVNS on measures of intestinal and systemic inflammation. This will explore differences in efficacy between randomised arms, which will assist with the design of the final intervention. Recruitment to this sub-study will take place at the lead site only (St. James's University Hospital). Additional eligibility criteria will apply, as outlined in Table 4. Bloods draws will be performed at baseline, 2 hours, 24 hours, and 48 hours after the

For peer review only

start of surgery. Peritoneal lavage fluid will also be collected at the start and end of surgery. Lavages will be performed by instilling 20ml of sterile 0.9% NaCl onto the small bowel using a foley catheter and bladder tip syringe, followed by collection of 5-10ml of fluid after 30 seconds, as described previously (22). Serum (systemic) and peritoneal lavage (intestinal) levels of tumour necrosis factor (TNF) alpha, interleukin (IL) 1-beta, and IL-6 will be analysed using enzyme-linked immunosorbent assays (ELISA) (23, 24).

– *Bowel motility sub-study*

The motility sub-study will explore the effect of nVNS on global small bowel motility. Differences in efficacy between randomised groups will inform the design of the final intervention. Recruitment will take place at the lead site only. Additional eligibility criteria (Table 4) will apply with a recruitment target of 10 participants per group (total sample: 40 participants). Magnetic resonance (MR) enterography will be performed at baseline and on the third post-operative day in the supine position under breath hold conditions whilst a series of motility acquisitions are performed. Oral preparation with a single glass of water will take place 30 minutes prior to the scan. These will be registered with dedicated software and a quantitative motility score generated to depict bowel wall motion (25).

– *Qualitative sub-study*

A qualitative sub-study will explore participants' acceptability of the trial recruitment process, intervention, their experiences of self-administration, and any challenges with device blinding. Recruitment will take place at the lead site with a target sample of 5 participants per group (total sample: 20 participants). In addition, 5 patients who declined participation in the study will be recruited to explore barriers to study enrolment. A maximum variation approach to sampling will be used to ensure that a wide variety of perspectives are captured, account for age, sex, and indication for

For peer review only

surgery). Semi-structured interviews will be conducted after hospital discharge in hospital or in the community. Questions will be informed by a topic guide and will be recorded in preparation for transcription. A thematic framework approach will be used for analysis, comprising five key stages: I) familiarisation with data; II) identifying the thematic framework; III) indexing; IV) charting; and V) mapping and interpreting (26). The thematic analysis will be modified in light of new data and a process of constant comparison will be used to examine across themes and cases. Qualitative data will be managed in NVivo® 11 (Melbourne, Australia).

For peer review only

Discussion

Although Ileus is a common problem after surgery, limited progress has been made to curtail its impact on patients and healthcare systems in the last 20 years (6). The most successful measures have been the widespread introduction of minimally invasive surgery and the development of evidence-based enhanced recovery protocols. These gains are likely facilitated through reduced inflammatory- and opioid-induced intestinal smooth muscle dysfunction, leading to faster recovery of bowel function (27, 28). The use of vagus nerve stimulation to reduce ileus is a new concept which draws on similar principles. Its efficacy has been demonstrated consistently in pre-clinical studies, where stimulation of the vagus nerve has been shown to reduce intestinal inflammation and restore small bowel transit through activation of a cholinergic anti-inflammatory pathway (11). In keeping with this, intra-operative stimulation of the vagus nerve has been shown to attenuate systemic markers of inflammation in patients undergoing major abdominal surgery (14). Taken together, this suggests that the anti-inflammatory mechanism of vagus nerve stimulation is directly translatable to humans and may represent a novel approach to reduce ileus after surgery.

Most previous studies of vagus nerve stimulation have involved surgical access to the neck and abdomen. This is challenging for clinical translation because the procedure is invasive and technically demanding. nVNS aims to mitigate these challenges by facilitating non-invasive stimulation over the cervical surface landmark. Stimulation can be performed before and after surgery, which is attractive since some evidence suggests that activation of the cholinergic anti-inflammatory pathway (previously using prucalopride) is most effective when done in the pre-operative period (29). Stimulation can also be performed on multiple occasions rather than a single intra-operative episode, which increases the opportunity for intervention. A schedule of five days is used in the present study as this represents the time period during which most patients are expected to regain bowel function (2). In addition, stimulation can be self-administered by patients which encourages active participation in recovery. Previous evidence has shown that active participation is a key motivator of recovery after surgery (30).

For peer review only

There are several possible challenges associated with studying and implementing nVNS in this setting. Firstly, self-administration requires adequate skill, understanding, and compliance, which may be difficult in the post-operative setting. A dedicated programme of training, including device positioning and administration, aims to mitigate this. Secondly, the non-invasive approach to stimulation is less precise than conventional stimulation since contact with the vagus nerve cannot be confirmed objectively. This is mitigated through the use of a standard technique (stimulation over the cervical surface landmark), which has been shown to successfully increase heart rate variability, reduce markers of systemic inflammation, and generate vagal somatosensory evoked potentials (indicators of vagal stimulation) in healthy volunteers (31-34). Finally, identifying an adequate sham intervention by which to compare nVNS is challenging. In previous healthy volunteer studies, nVNS has been shown to significantly reduce the expression of inflammatory cytokines from baseline but not when using the sham device (32). It is possible that manual instrumentation of the device may be sufficient to elicit some vagal activity, but this is considered minimal and is comparable across both device types. Providing that these challenges are addressed, nVNS may provide a practical approach to stimulating the vagus nerve in the pre- and post-operative setting.

The present study will inform the feasibility of a phase-III randomised controlled trial of nVNS by addressing key challenges in trial design. This includes compliance to the stimulation schedule, which is important in the setting of major abdominal surgery where unique barriers may exist. It also includes the adequacy of participant blinding. This is important in studies of electrical nerve stimulation, where sham-controls are often associated with statistical improvements in outcomes (35). Some issues of feasibility are beyond the scope of this study. The selection of outcomes for ileus are poorly standardised, with over 50 different outcomes identified from previous literature in the last 20 years (36). Such wide variation is challenging for evaluating new treatments and translating these into clinical practice. To address this, an international core outcome set for gastrointestinal recovery after major abdominal surgery is under development and will provide a standardised approach to outcome reporting in the

For peer review only

future (37). In addition, the feasibility of implementing nVNS into enhanced recovery protocols across different settings is unclear. The present study explores the feasibility of nVNS in two large teaching hospitals, but further consideration of barriers to implementation will be required.

For peer review only

Ethics approval: Research ethics approval was confirmed by the North East - Tyne & Wear South NHS Research Ethics Committee (19/NE/0217) on 2nd July 2019. Written, informed consent to participate will be obtained from all participants.

Availability of data and materials: A model consent form is available on request.

Competing interests: No competing interests are declared. The views expressed in this publication are those of the authors and not necessarily those of the NHS, the National Institute for Health Research, Health Education England, or the Department of Health.

Funding: This study is funded by a National Institute for Health Research Doctoral Research Fellowship (S Chapman; DRF-2018-11-049). Study devices are provided through the manufacturer's (electroCore™, Inc) Investigator Initiated Trial Programme.

Contributions: SJC and DJ conceptualised the study and all authors (SJC, MN, CJCM, DT, DDS, and DJ) contributed to its design. SJC prepared the protocol draft, which was reviewed and approved by all authors (SJC, MN, CJCM, DT, DDS, and DJ). The Clinical Trials Research Unit at University of Leeds oversees randomisation and University of Leeds is the study sponsor. DDS is the statistical guarantor and DJ is the overall study guarantor.

Copyright: The Corresponding Author has the right to grant on behalf of all authors and does grant on behalf of all authors, a worldwide licence to the Publishers and its licensees in perpetuity, in all forms, formats and media (whether known now or created in the future), to i) publish, reproduce, distribute, display and store the Contribution, ii) translate the Contribution into other languages, create adaptations, reprints, include within collections and create summaries, extracts and/or, abstracts of the Contribution, iii) create any other derivative work(s) based on the Contribution, iv) to exploit all subsidiary rights in the Contribution, v) the inclusion of electronic links from the Contribution to third party material where-ever it may be located; and, vi) licence any third party to do any or all of the above.

Acknowledgements: None

For peer review only

References

1. Scarborough JE. Schumacher J. Kent KC. Heise CP. Greenberg CC. Associations of Specific Postoperative Complications With Outcomes After Elective Colon Resection: A Procedure-Targeted Approach Toward Surgical Quality Improvement. JAMA Surg 2017;152:e164681.
2. Yorkshire Surgical Research Collaborative. Multicentre observational study of gastrointestinal recovery after elective colorectal surgery. Colorectal Dis 2018;20:536-544.
3. Tevis SE. Carchman EH. Foley EF. Harms BA. Heise CP. Kennedy GD. Postoperative Ileus-More than Just Prolonged Length of Stay? J Gastrointest Surg. 2015;19:1684-90.
4. Mao H. Milne TGE. O'Grady G. Vather R. Edlin R. Bissett I. Prolonged Postoperative Ileus Significantly Increases the Cost of Inpatient Stay for Patients Undergoing Elective Colorectal Surgery: Results of a Multivariate Analysis of Prospective Data at a Single Institution. Dis Colon Rectum. 2019;62:631-637.
5. Tiernan J, Cook A, Geh I. et al. Use of a modified Delphi approach to develop research priorities for the association of coloproctology of Great Britain and Ireland. Colorectal Dis 2014;16:965-975.
6. Chapman SJ. Pericleous A. Downey C. Jayne DG. Postoperative ileus following major colorectal surgery. Br J Surg 2018;105:797-810.
7. Herbert G. Perry R. Andersen HK. Atkinson C. Penfold C. Lewis SJ, et al. Early enteral nutrition within 24 hours of lower gastrointestinal surgery versus later commencement for length of hospital stay and postoperative complications. Cochrane Database Syst Rev 2019: CD004080.
8. Short V. Herbert G. Perry R. Atkinson C. Ness AR. Penfold C et al. Chewing gum for postoperative recovery of gastrointestinal function. Cochrane Database Syst Rev 2015: CD006506.
9. Kranke P. Jokinen J. Pace NL. Schnabel A. Hollmann MW. Hahnenkamp K. et al. Continuous intravenous perioperative lidocaine infusion for postoperative pain and recovery. Cochrane Database Syst Rev 2015 :CD009642
10. Chapman SJ. Wells CI. Challenges in ileus research. Colorectal Dis 2018;20:639.
11. Matteoli G. Gomez-Pinilla PJ. Nemethova. A. Di Giovangiulio M. Cailotto C. van Bree SH. et al. A distinct vagal anti-inflammatory pathway modulates intestinal muscularis resident macrophages independent of the spleen. Gut 2014;63:938–948.
12. de Jonge WJ. van der Zanden EP. The FO. Bijlsma MF. van Westerloo DJ. et al. Stimulation of the vagus nerve attenuates macrophage activation by activating the Jak2-STAT3 signalling pathway. Nat Immunol 2005;6:844-851.
13. Bonaz B. Sinniger V. Pellissier S. The Vagus Nerve in the Neuro-Immune Axis: Implications in the Pathology of the Gastrointestinal Tract. Front Immunol 2017;8:1452.

14. Stakenborg N. Wolthuis AM. Gomez-Pinilla PJ. Farro G. Di Giovangiulio M. Bosmans G. et al. Abdominal vagus nerve stimulation as a new therapeutic approach to prevent postoperative ileus. Neurogastro & Motility. 2017;29:e13075.
15. Hong G. Pintea B. Lingohr P. Coch C. Randau T. Schaefer N. et al. Effect of transcutaneous vagus nerve stimulation on muscle activity in the gastrointestinal tract (transVaGa): a prospective clinical trial. International J of Colorectal Dis 2019;34:417–422.
16. Chan AW. Tetzlaff JM. Altman DG, et al. SPIRIT 2013 statement: defining standard protocol items for clinical trials. Ann Intern Med. 2013;158:200-7.
17. Bang H. Ni L. Davis CE. Assessment of blinding in clinical trials. Control Clin Trials 2004;25:143-156.
18. van Bree SH. Bemelman WA. Hollmann MW. Zwinderman AH. Matteoli G. El Temna S. et al. Identification of clinical outcome measures for recovery of gastrointestinal motility in postoperative ileus. Ann Surg 2014;259;708-714.
19. Teare MD. Dimairo M. Shephard N. Hayman A. Whitehead A. Walters SJ. Sample size requirements to estimate key design parameters from external pilot randomised controlled trials: a simulation study. Trials 2014;15:264.
20. Gamble C. Krishan A. Stocken D. Guidelines for the Content of Statistical Analysis Plans in Clinical Trials. JAMA 2017;318:2337-2343.
21. Moher D. Hopewell S. Schulz KF. CONSORT 2010 explanation and elaboration: updated guidelines for reporting parallel group randomised trials. BMJ 2010;340:c869
22. The FO. Bennink RJ. Ankum WM. et al. Intestinal handling-induced mast cell activation and inflammation in human postoperative ileus. Gut 2008;57:33-40.
23. Lange T. Dimitrov S. Born J. Effect of sleep and circadian rhythm on the human immune system. Ann N Y Acad Sci 210;1193:48-59.
24. Baigrie J. Lamont PM. Kwiatkowski D. Dallman MJ. Morris PJ. Systemic cytokine response after major surgery. Br J Surgery 1992;79:757-60.
25. Menys A. Taylor SA. Emmanuel A. Ahmed A. Plumb AA. Odille F. Alam A. Halligan S. Atkinson D. Global small bowel motility: assessment with dynamic MR imaging. Radiology. 2013;269:443-50.
26. Ritchie J. Lewis J. McNaughton Nicholls C. Ormston R. Qualitative Research Practice: A Guide for Social Science Students and Researchers 2nd Ed. Sage. London. 2014
27. Schwenk W. Jacobi C. Mansmann U. Böhm B. Müller JM. Inflammatory response after laparoscopic and conventional colorectal resections - results of a prospective randomized trial. Langenbecks Arch Surg 2000;385:2-9.
28. Venara A. Duchalais E. Dariel A. Aubert P. Durand T. Meurette G. Rolli-Derkinderen M. Hamy A. Neunlist M. Anti-inflammatory Effects of Enhanced Recovery Programs on Early-Stage Colorectal Cancer Surgery. World J Surg 2018;42:953-964.

For peer review only

29. Stakenborg N. Labeeuw E. Gomez-Pinilla PJ. De Schepper S. Aerts R. et al. Preoperative administration of the 5-HT4 receptor agonist prucalopride reduces intestinal inflammation and shortens postoperative ileus via cholinergic enteric neurons. Gut 2019;68:1406-1416.
30. Chapman SJ. Czoski Murray CJ. Lonsdale MDS. Boyes S. Tiernan JP. et al. Information needs for recovery after colorectal surgery: Patient focus group study. *Colorectal Dis* 2020 (In Press).
31. Brock C. Brock B. Aziz Q. Møller HJ. Pfeiffer Jensen M. Drewes AM. Farmer AD. Transcutaneous cervical vagal nerve stimulation modulates cardiac vagal tone and tumor necrosis factor-alpha. Neurogastroenterol Motil 2017;29. doi: 10.1111/nmo.12999.
32. Lerman I. Hauger R. Sorkin L. Proudfoot J. Davis B. et al. Noninvasive Transcutaneous Vagus Nerve Stimulation Decreases Whole Blood Culture-Derived Cytokines and Chemokines: A Randomized, Blinded, Healthy Control Pilot Trial. Neuromodulation 2016;19:283-90.
33. Vecchio E. Bassez I. Ricci K. Tassorelli C. Liebler E. et al. Effect of Non-invasive Vagus Nerve Stimulation on Resting-State Electroencephalography and Laser-Evoked Potentials in Migraine Patients: Mechanistic Insights. Front Hum Neurosci 2018;13;12:366.
34. Nonis R. D'Ostilio K. Schoenen J. Magis D. Evidence of activation of vagal afferents by non-invasive vagus nerve stimulation: An electrophysiological study in healthy volunteers. *Cephalgia* 2017;37:1285-1293.
35. Tan K. Wells CI. Dinning P. Bissett IP. O'Grady G. Placebo Response Rates in Electrical Nerve Stimulation Trials for Fecal Incontinence and Constipation: A Systematic Review and Meta-Analysis. Neuromodulation 2019. doi: 10.1111/ner.13092. [Epub ahead of print]
36. Chapman SJ. Thorpe G. Vallance AE. Harji DP. Lee MJ. et al. Systematic review of definitions and outcome measures for return of bowel function after gastrointestinal surgery. BJS Open 2018;3:1-10.
37. Chapman SJ. Lee MJ. Blackwell S. Arnott R. Ten Broek RPG. et al. Establishing core outcome sets for gastrointestinal recovery in studies of postoperative ileus and small bowel obstruction: protocol for a nested methodological study. Colorectal Dis. 2019. doi: 10.1111/codi.14899. [Epub ahead of print]

For peer review only

2

Table 1 – Eligible Procedures

**Right sided resection**	**Left sided resection**
Ileocolic/caecal resection	Extended left hemicolectomy
Right hemicolectomy	Left hemicolectomy
Extended right hemicolectomy	Sigmoid colectomy
Transverse colectomy	Rectosigmoid colectomy
**Miscellaneous (side grouped accordingly)**	Anterior resection*
Other segmental colonic resection	

For peer review only

Table 2 – Progression criteria

Criteria	Stop	Adjust	Progress
Proportion of eligible patients identified from screening logs	<10%	10-20%	>20%
Number of eligible patients randomised over 24 months (site: SJUH)	≤2 per month	3-4 per month	≥5 per month
Number of eligible patients randomised over 18 months (site: BRI)	<1 per month	1-2 per month	≥3 per month
Adequacy of participant blinding (according to Bang Blinding Index)	Index < -0.5<br>Or Index > 0.5	Index -0.2 to -0.5<br>Or Index 0.2 to 0.5	Index 0 to -0.19<br>Or Index 0 to 0.19
Average compliance to the study treatment schedule *	<10/20 stimulations across 10 days	10-15/20 stimulations across 10 days	≥16/20 stimulations across 10 days
Proportion of missing clinical outcome data	>40%	15-30%	<15%
Proportion of randomised patients lost to follow up	>40%	15-30%	<15%
Incidence of complications or serious complications	>20% increase in complications	5-20% increase in complications	<5% increase in complications

BRI: Bradford Royal Infirmary, Bradford; SJUH: St. James's University Hospital, Leeds

For peer review only

Table 3 – Schedule of Events

	Events	Baseline	Preop study visit	5 days before surgery	Day of surgery (pre-op)	Day of surgery (Intraop)	POD 1	POD 2	POD 3	POD 4	POD 5	POD 6-10	After discharge	30-day phone F/U
Study Procedures	Trial consent	✓												
	Device training		✓											
	Device distribution		✓		✓									
	Self-administered stimulation			✓			✓	✓	✓	✓	✓			
	Stimulation diary			✓			✓	✓	✓	✓	✓			
	Blinding poll										✓			
Data collection	Eligibility CRF	✓												
	Baseline CRF	✓												
	Operative CRF					✓								
	Gastrointestinal Function CRF						✓	✓	✓	✓	✓	✓		
	Follow-up CRF													✓
Sub-studies*	*Optional sub-study consents*	✓												
	Inflammatory response study<br>Blood only: ✓; Blood and fluid ✓✓				✓	✓✓	✓		✓					
	Small bowel motility study (MRI)		✓						✓					
	Qualitative sub-study (participant or non-participant interview)												✓	

**Sub-studies are optional and undertaken at the lead site only*
CRF: Case Report Form; F/U: follow up; MRI: magnetic resonance imaging; POD: postoperative day

For peer review only

Table 4 – Sub-study additional exclusion criteria

**Inflammatory response sub-study**
• Regular use of non-steroidal anti-inflammatory drugs in the preceding 7 days<br>• Medical condition requiring oral or injectable steroids<br>• Neoadjuvant chemoradiotherapy within 12 months prior to surgery
**Bowel motility sub-study**
• Indwelling pacemaker or cardiac defibrillator device<br>• Non-MR compatible metallic implants, prostheses, neurosurgical clips, indwelling stimulator or pump devices, which would preclude safe MRI<br>• Claustrophobia<br>• Metallic foreign bodies in the eyes

MRI: Magnetic Resonance Imaging

For peer review only

Figure 1 – Study flow chart

For peer review only

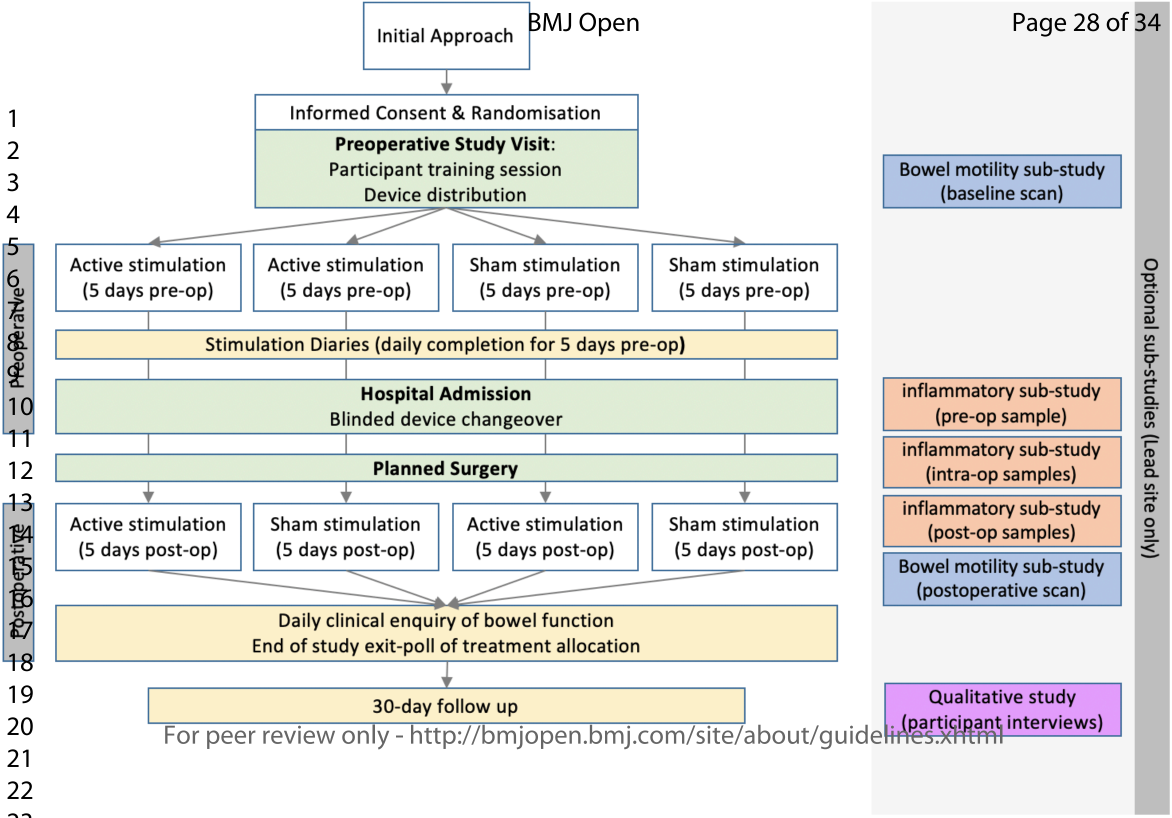

For peer review only - http://bmjopen.bmj.com/site/about/guidelines.xhtml

Supplementary File

Study Status

The study first began recruitment on 2nd January 2020 and is expected to complete by 31st August 2021. The current protocol version is v1.2 (13th February 2020).

Auditing

The conduct of the trial will be audited monthly by the principal investigator. In line with Good Clinical Practice, a study site folder will be held at each participating recruitment site. This will contain the current study protocol, delegation log outlining responsibilities of all research team members, and all study correspondence. Data relating to recruitment will be uploaded to the NIHR Central Portfolio Management System on a monthly basis.

Adverse Events

The reporting of adverse events will be done in line with Good Clinical Practice. All serious adverse events will be reported to the sponsor within 24 hours of research staff becoming aware of the event. Serious adverse events that are considered to be unexpected and related to the study will be escalated to the research ethics committee.

Study Amendments

If amendments to the study protocol or documents are required, this will be done and communicated in line with Health Research Authority guidelines. All amendments will be categorised and submitted for review by the Research Ethics Committee, as appropriate.

Dissemination Plan

The findings of the study and sub-studies will be disseminated at academic conferences and in peer-reviewed manuscripts. The results will also be summarised in plain English visual abstracts and disseminated to research participants and the public.

For peer review only

Study Sponsor

The sponsor for this study is University of Leeds

Recruitment & Protocol Materials

Study recruitment and protocol materials are available upon request from the corresponding author.

For peer review only

SPIRIT

STANDARD PROTOCOL ITEMS: RECOMMENDATIONS FOR INTERVENTIONAL TRIALS

SPIRIT 2013 Checklist: Recommended items to address in a clinical trial protocol and related documents*

Section/item	Item No	Description
**Administrative information**		
Title	1	Descriptive title identifying the study design, population, interventions, and, if applicable, trial acronym **(Page 1)**
Trial registration	2a	Trial identifier and registry name. If not yet registered, name of intended registry **(Page 5)**
	2b	All items from the World Health Organization Trial Registration Data Set **(Page 5)**
Protocol version	3	Date and version identifier **(Page 5)**
Funding	4	Sources and types of financial, material, and other support **(Page 1)**
Roles and responsibilities	5a	Names, affiliations, and roles of protocol contributors **(Page 1)**
	5b	Name and contact information for the trial sponsor **(Suppl. File 1)**
	5c	Role of study sponsor and funders, if any, in study design; collection, management, analysis, and interpretation of data; writing of the report; and the decision to submit the report for publication, including whether they will have ultimate authority over any of these activities **(Page 1)**
	5d	Composition, roles, and responsibilities of the coordinating centre, steering committee, endpoint adjudication committee, data management team, and other individuals overseeing the trial, if applicable (see Item 21a for data monitoring committee) **(N/A)**
**Introduction**		
Background and rationale	6a	Description of research question and justification for undertaking the trial, including summary of relevant studies (published and unpublished) examining benefits and harms for each intervention **(Page 3-4)**
	6b	Explanation for choice of comparators **(Page 3-4)**
Objectives	7	Specific objectives or hypotheses **(Page 5)**

For peer review only - http://bmjopen.bmj.com/site/about/guidelines.xhtml

Trial design	8	Description of trial design including type of trial (eg, parallel group, crossover, factorial, single group), allocation ratio, and framework (eg, superiority, equivalence, noninferiority, exploratory) **(Page 5)**

Methods: Participants, interventions, and outcomes

Study setting	9	Description of study settings (eg, community clinic, academic hospital) and list of countries where data will be collected. Reference to where list of study sites can be obtained **(Page 6)**
Eligibility criteria	10	Inclusion and exclusion criteria for participants. If applicable, eligibility criteria for study centres and individuals who will perform the interventions (eg, surgeons, psychotherapists) **(Page 6)**
Interventions	11a	Interventions for each group with sufficient detail to allow replication, including how and when they will be administered **(Page 7)**
	11b	Criteria for discontinuing or modifying allocated interventions for a given trial participant (eg, drug dose change in response to harms, participant request, or improving/worsening disease) **(N/A)**
	11c	Strategies to improve adherence to intervention protocols, and any procedures for monitoring adherence (eg, drug tablet return, laboratory tests) **(N/A)**
	11d	Relevant concomitant care and interventions that are permitted or prohibited during the trial **(Page 7)**
Outcomes	12	Primary, secondary, and other outcomes, including the specific measurement variable (eg, systolic blood pressure), analysis metric (eg, change from baseline, final value, time to event), method of aggregation (eg, median, proportion), and time point for each outcome. Explanation of the clinical relevance of chosen efficacy and harm outcomes is strongly recommended **(Page 7-8)**
Participant timeline	13	Time schedule of enrolment, interventions (including any run-ins and washouts), assessments, and visits for participants. A schematic diagram is highly recommended (see Figure) **(Page 8-9)**
Sample size	14	Estimated number of participants needed to achieve study objectives and how it was determined, including clinical and statistical assumptions supporting any sample size calculations **(Page 9)**
Recruitment	15	Strategies for achieving adequate participant enrolment to reach target sample size **(Page 9)**

For peer review only - http://bmjopen.bmj.com/site/about/guidelines.xhtml

Methods: Assignment of interventions (for controlled trials)

Allocation:

Sequence generation	16a	Method of generating the allocation sequence (eg, computer-generated random numbers), and list of any factors for stratification. To reduce predictability of a random sequence, details of any planned restriction (eg, blocking) should be provided in a separate document that is unavailable to those who enrol participants or assign interventions **(Page 9)**
Allocation concealment mechanism	16b	Mechanism of implementing the allocation sequence (eg, central telephone; sequentially numbered, opaque, sealed envelopes), describing any steps to conceal the sequence until interventions are assigned **(Page 9)**
Implementation	16c	Who will generate the allocation sequence, who will enrol participants, and who will assign participants to interventions **(Page 9)**
Blinding (masking)	17a	Who will be blinded after assignment to interventions (eg, trial participants, care providers, outcome assessors, data analysts), and how **(Page 9-10)**
	17b	If blinded, circumstances under which unblinding is permissible, and procedure for revealing a participant's allocated intervention during the trial **(N/A)**

Methods: Data collection, management, and analysis

Data collection methods	18a	Plans for assessment and collection of outcome, baseline, and other trial data, including any related processes to promote data quality (eg, duplicate measurements, training of assessors) and a description of study instruments (eg, questionnaires, laboratory tests) along with their reliability and validity, if known. Reference to where data collection forms can be found, if not in the protocol **(Page 10)**
	18b	Plans to promote participant retention and complete follow-up, including list of any outcome data to be collected for participants who discontinue or deviate from intervention protocols
Data management	19	Plans for data entry, coding, security, and storage, including any related processes to promote data quality (eg, double data entry; range checks for data values). Reference to where details of data management procedures can be found, if not in protocol **(Page 10)**
Statistical methods	20a	Statistical methods for analysing primary and secondary outcomes. Reference to where other details of the statistical analysis plan can be found, if not in the protocol **(Page 10)**
	20b	Methods for any additional analyses (eg, subgroup and adjusted analyses) **(Page 10)**

For peer review only - http://bmjopen.bmj.com/site/about/guidelines.xhtml

	20c	Definition of analysis population relating to protocol non-adherence (eg, as randomised analysis), and any statistical methods to handle missing data (eg, multiple imputation) **(Page 10)**
**Methods: Monitoring**		
Data monitoring	21a	Composition of data monitoring committee (DMC); summary of its role and reporting structure; statement of whether it is independent from the sponsor and competing interests; and reference to where further details about its charter can be found, if not in the protocol. Alternatively, explanation of why a DMC is not needed **(Suppl. File 1)**
	21b	Description of any interim analyses and stopping guidelines, including who will have access to these interim results and make the final decision to terminate the trial **(Suppl. File 1)**
Harms	22	Plans for collecting, assessing, reporting, and managing solicited and spontaneously reported adverse events and other unintended effects of trial interventions or trial conduct **(Suppl. File 1)**
Auditing	23	Frequency and procedures for auditing trial conduct, if any, and whether the process will be independent from investigators and the sponsor **(Suppl. File 1)**
**Ethics and dissemination**		
Research ethics approval	24	Plans for seeking research ethics committee/institutional review board (REC/IRB) approval **(Page 5)**
Protocol amendments	25	Plans for communicating important protocol modifications (eg, changes to eligibility criteria, outcomes, analyses) to relevant parties (eg, investigators, REC/IRBs, trial participants, trial registries, journals, regulators) **(Suppl. File 1)**
Consent or assent	26a	Who will obtain informed consent or assent from potential trial participants or authorised surrogates, and how (see Item 32) **(Page 9)**
	26b	Additional consent provisions for collection and use of participant data and biological specimens in ancillary studies **(Page 11-12)**
Confidentiality	27	How personal information about potential and enrolled participants will be collected, shared, and maintained in order to protect confidentiality before, during, and after the trial **(Page 10)**
Declaration of interests	28	Financial and other competing interests for principal investigators for the overall trial and each study site **(Page 1)**
Access to data	29	Statement of who will have access to the final trial dataset, and disclosure of contractual agreements that limit such access for investigators **(N/A)**
Ancillary and post-trial care	30	Provisions, if any, for ancillary and post-trial care, and for compensation to those who suffer harm from trial participation **(N/A)**

For peer review only - http://bmjopen.bmj.com/site/about/guidelines.xhtml

Dissemination policy	31a	Plans for investigators and sponsor to communicate trial results to participants, healthcare professionals, the public, and other relevant groups (eg, via publication, reporting in results databases, or other data sharing arrangements), including any publication restrictions **(Suppl. File 1)**
	31b	Authorship eligibility guidelines and any intended use of professional writers **(Suppl. File 1)**
	31c	Plans, if any, for granting public access to the full protocol, participant-level dataset, and statistical code **(Suppl. File 1)**
**Appendices**		
Informed consent materials	32	Model consent form and other related documentation given to participants and authorised surrogates **(Suppl. File 1)**
Biological specimens	33	Plans for collection, laboratory evaluation, and storage of biological specimens for genetic or molecular analysis in the current trial and for future use in ancillary studies, if applicable **(Pages 11-12)**

*It is strongly recommended that this checklist be read in conjunction with the SPIRIT 2013 Explanation & Elaboration for important clarification on the items. Amendments to the protocol should be tracked and dated. The SPIRIT checklist is copyrighted by the SPIRIT Group under the Creative Commons "Attribution-NonCommercial-NoDerivs 3.0 Unported" license.

For peer review only

Non-invasive, vagus nerve stimulation to reduce ileus after colorectal surgery: Protocol for a feasibility trial with nested mechanistic studies

Journal:	*BMJ Open*
Manuscript ID	bmjopen-2020-046313.R2
Article Type:	Protocol
Date Submitted by the Author:	26-Jan-2021
Complete List of Authors:	Chapman, Stephen; University of Leeds, Leeds Institute of Medical Research<br>Naylor, Maureen; West Riding of Yorkshire Ileostomy Association<br>Czoski Murray, Carolyn; University of Leeds, Leeds Institute of Health Sciences<br>Tolan, Damian; Leeds Teaching Hospitals NHS Trust<br>Stocken, Deborah; University of Leeds, Leeds Institute of Clinical Trials Research<br>Jayne, David; University of Leeds, Leeds Institute of Medical Research
**Primary Subject Heading**:	Surgery
Secondary Subject Heading:	Gastroenterology and hepatology, Qualitative research, Research methods
Keywords:	SURGERY, GASTROENTEROLOGY, NEUROPHYSIOLOGY

SCHOLARONE™ Manuscripts

I, the Submitting Author has the right to grant and does grant on behalf of all authors of the Work (as defined in the below author licence), an exclusive licence and/or a non-exclusive licence for contributions from authors who are: i) UK Crown employees; ii) where BMJ has agreed a CC-BY licence shall apply, and/or iii) in accordance with the terms applicable for US Federal Government officers or employees acting as part of their official duties; on a worldwide, perpetual, irrevocable, royalty-free basis to BMJ Publishing Group Ltd ("BMJ") its licensees and where the relevant Journal is co-owned by BMJ to the co-owners of the Journal, to publish the Work in this journal and any other BMJ products and to exploit all rights, as set out in our licence.

The Submitting Author accepts and understands that any supply made under these terms is made by BMJ to the Submitting Author unless you are acting as an employee on behalf of your employer or a postgraduate student of an affiliated institution which is paying any applicable article publishing charge ("APC") for Open Access articles. Where the Submitting Author wishes to make the Work available on an Open Access basis (and intends to pay the relevant APC), the terms of reuse of such Open Access shall be governed by a Creative Commons licence – details of these licences and which Creative Commons licence will apply to this Work are set out in our licence referred to above.

Other than as permitted in any relevant BMJ Author's Self Archiving Policies, I confirm this Work has not been accepted for publication elsewhere, is not being considered for publication elsewhere and does not duplicate material already published. I confirm all authors consent to publication of this Work and authorise the granting of this licence.

For peer review only

Non-invasive, vagus nerve stimulation to reduce ileus after colorectal surgery: Protocol for a feasibility trial with nested mechanistic studies

Stephen J Chapman[1]. Maureen Naylor [2]. Carolyn J Czoski Murray[3]. Damian Tolan[4]. Deborah D Stocken[5]. David Jayne[6].

1. Leeds Institute of Medical Research, University of Leeds, UK; S.Chapman@leeds.ac.uk
2. West Riding of Yorkshire Ileostomy Association; m.naylor70@btinternet.com
3. Leeds Institute of Health Sciences, University of Leeds, UK; C.J.CzoskiMurray@leeds.ac.uk
4. Leeds Teaching Hospitals NHS Trust, Leeds, UK; Damian.Tolan@nhs.net
5. Leeds Institute for Clinical Trials Research, University of Leeds, UK: D.D.Stocken@leeds.ac.uk
6. Leeds Institute of Medical Research, University of Leeds, UK; D.G.Jayne@leeds.ac.uk

Corresponding Author

Stephen J Chapman, Room 7.16 Clinical Sciences Building, St. James's University Hospital, University of Leeds, LS2 9JT, United Kingdom

Email: S.Chapman@leeds.ac.uk; Twitter: @SJ_Chapman; ORCID: 0000-0003-2413-5690

Running title: Non-invasive vagus nerve stimulation to reduce ileus

Category: Protocol

Main body word count: 3,308

Keywords: ileus, colorectal surgery, vagus nerve stimulation, Feasibility RCT

For peer review only

Abstract

Introduction: Ileus is a common and distressing condition characterised by gut dysfunction after surgery. Whilst a number of interventions have aimed to curtail its impact on patients and healthcare systems, ileus is still an unmet challenge. Electrical stimulation of the vagus nerve is a promising new treatment due to its role in modulating the neuro-immune axis through a novel anti-inflammatory reflex. The protocol for a feasibility study of non-invasive vagus nerve stimulation (nVNS), and a programme of mechanistic and qualitative studies, is described.

Methods and Analysis: This is a participant-blinded, parallel-group, randomised, sham-controlled feasibility trial (IDEAL Stage 2b) of self-administered nVNS. One hundred and forty patients planned for elective, minimally-invasive, colorectal surgery, will be randomised to four schedules of nVNS before and after surgery. Feasibility outcomes include assessments of recruitment and attrition, adequacy of blinding, and compliance to the intervention. Clinical outcomes include bowel function and length of hospital stay. A series of mechanistic sub-studies exploring the impact of nVNS on inflammation and bowel motility will inform the design of the final stimulation schedule. Semi-structured interviews with participants will explore experiences and perceptions of the intervention, whilst interviews with patients who decline participation will explore barriers to recruitment.

Ethics and Dissemination: The protocol has been approved by the Tyne & Wear South NHS Research Ethics Committee (19/NE/0217) on 2nd July 2019. Feasibility, mechanistic, and qualitative findings will be disseminated to national and international partners through peer-reviewed publications, academic conferences, social media channels, and stakeholder engagement activities. The findings will build a case for or against progression to a definitive randomised assessment as well as informing key elements of study design.

Trial Registration: ISRCTN; ISRCTN62033341; Prospectively registered on 11th October 2019; http://www.isrctn.com/ISRCTN62033341.

For peer review only

Strengths and limitations of this study

- Feasibility outcomes will support the development of a definitive, randomised controlled trial, including considerations of recruitment, device compliance, and sham-blinding.
- Qualitative outcomes will explore possible barriers to participation and compliance with the self-administered device before and after colorectal surgery
- The study will not explore the most appropriate outcome to be used in a definitive randomised controlled trial, but other related work done in parallel will fill this need.

For peer review only

Introduction

Ileus is a distressing condition which occurs in 10-20% of patients undergoing major abdominal surgery (1). It is characterised by painful abdominal distension, persistent vomiting, and delayed bowel function. This usually resolves 2-4 days after surgery, but in some cases, it may persist in excess of 10 days (2). The impact of ileus is extensive. For patients, it increases the length of hospital stay and increases the risk of serious postoperative complications such as pneumonia, surgical site infection, and venous thrombo-embolic events (3). For hospitals, it is economically burdensome and is associated with an increase of 71% in healthcare costs (4). Once considered to be a normal consequence of surgical recovery, the Association of Coloproctology in Great Britain & Ireland now considers ileus to be a research priority (5).

In the last 20 years, a number of interventions to prevent ileus and its clinical sequelae have been explored (6). Strategies to encourage early or sham-feeding (chewing gum) have been studied extensively but evidence for their effectiveness remains contentious (7, 8). New approaches to perioperative management (such as intravenous lidocaine) have been explored but uncertainty relating to efficacy, dose, and timing persist (9). Novel drugs such as mu-opioid receptor antagonists have shown promising results but economic and regulatory barriers have limited their uptake beyond North America. The consensus of existing literature suggests that minimally-invasive surgery and protocol-driven recovery offer the best chances of preventing ileus (10). Uncertainty exists over the pathophysiology of ileus, however, which is problematic for designing clinical interventions that are justified by strong scientific evidence.

Stimulation of the vagus nerve may represent a novel and evidence-based approach to reduce ileus after surgery. In pre-clinical models, electrical stimulation of the vagus nerve accelerates the recovery of gastrointestinal transit. This is facilitated through vagal afferents targeting the hypothalamic-pituitary-adrenal axis and vagal efferents targeting the cholinergic anti-inflammatory pathway, which attenuate inflammatory-mediated gut dysfunction (11-13). Early clinical studies have suggested that this is directly translatable to humans undergoing surgery (14, 15). Challenges exist, however, as to how vagus nerve stimulation can be done safely,

For peer review only

how its efficacy can be optimised, and how patient-acceptability can be ensured. A protocol for a randomised feasibility trial of non-invasive vagus nerve stimulation (nVNS) to reduce ileus, as well as a series of mechanistic and qualitative studies, is described.

For peer review only

Methods

Ethics & Governance

Ethical approval for the study was obtained from North East - Tyne & Wear South Research Ethics Committee (19/NE/0217) on 2nd July 2019. The study was prospectively registered on the ISRCTN registry (ISRCTN62033341; http://www.isrctn.com/ISRCTN62033341) on 11th October 2019 prior to the start of recruitment (Supplementary File 1). The study devices will be provided through the manufacturer's (electroCore™, Inc) Investigator Initiated Trial Programme. The present manuscript is reported according to the Standard Protocol Items Recommendation For Interventional Trials (SPIRIT) 2013 Checklist (16).

Aims & Objectives

The aim of this study is to determine the feasibility of performing a multicentre, phase-III, randomised-controlled trial (RCT) of nVNS to reduce ileus after colorectal surgery. The following objectives will explore the case for progression to a phase-III trial:

- To estimate the proportion of patients screened who are eligible for approach
- To estimate the number of approached patients who consent to randomisation
- To assess the adequacy of participant blinding to the allocated intervention
- To assess participant compliance to the intervention schedule
- To explore the safety of the intervention schedule
- To estimate the rate of missing outcome data, rate of withdrawal, and loss to follow up

Study Design

This study is a participant-blinded, parallel-group, randomised, sham-controlled feasibility trial (IDEAL Stage 2b) of nVNS to reduce ileus after colorectal surgery (Figure 1). One hundred and forty participants will be recruited across two study sites. Participants will be randomised to four arms (1:1:1:1) according to the following stimulation schedules:

For peer review only

- Pre-operative stimulation and post-operative stimulation
- Pre-operative sham and post-operative stimulation
- Pre-operative stimulation and post-operative sham
- Pre-operative sham and post-operative sham

The estimated trial duration is 24 months and all participants will be followed up for 30 days after the date of surgery.

Study Setting

The intervention will take place within the community (pre-operative stimulation) and in hospital (post-operative stimulation). All participants will undergo surgery at one of two large teaching hospitals (St. James's University Hospital, Leeds; and Bradford Royal Infirmary, Bradford). Both provide elective colorectal cancer services including laparoscopic surgery within a programme of enhanced recovery.

Eligibility Criteria

To be considered eligible for the trial, patients must satisfy all of the following inclusion criteria:

- Aged ≥ 18 years
- Able to provide written informed consent
- Planned to undergo elective, minimally-invasive (laparoscopic or robotic) surgery with a planned anastomosis/no routine plans for a diverting stoma, including one of the procedures listed in Table 1.

Presence of any of the following exclusion criteria will preclude participation in the trial:

- Severe cardiac disease (myocardial infarction in the last 12 months; heart failure with New York Heart Association Scale >2; 2nd or 3rd degree atrioventricular block, permanent atrial fibrillation/flutter, or previous ventricular tachycardia/fibrillation).
- Seizures or recurrent episodes of syncope (>1) in the last five years

For peer review only

- Previous transient ischaemic attack or cerebral vascular accident
- Previous vagotomy at any anatomical location
- Confirmed diagnosis of chronic gastrointestinal inflammatory condition
- Confirmed diagnosis of neuro-endocrine tumour
- Existing gastrointestinal stoma
- Implanted electrical stimulator device (such as cardiac pacemaker or defibrillator
- Structural abnormality of the neck anatomy which may impact on use of the device
- Belonging to a vulnerable population (such as those lacking capacity, prisoners)
- Pregnant, nursing, or thinking of becoming pregnant during the study period

Participants will not be eligible for entry into other interventional studies which aim to optimise surgical recovery or for which the intervention may impact on bowel function.

Intervention

Non-invasive vagus nerve stimulation will be self-administered by participants using the GammaCore® device (electroCore™, LLC, New Jersey, USA). This provides a transcutaneous electrical stimulation comprising of 5 x 5KHz sine-wave complexes delivered over 1ms and repeated at 25Hz (maximum voltage: 24V; maximum output current: 60mA). The surface landmark of the carotid pulse is used to guide positioning of the device over the cervical vagus nerve. Participants will self-administer the device twice daily for five days before and after surgery, with each administration lasting for 2 minutes on either side of the neck. Participants will be instructed to adjust the stimulation setting to the maximum tolerated level. A sham device identical in appearance will be used as a control. This produces a sub-therapeutic stimulation (0.1Hz biphasic direct current) whilst providing a perceptible stimulation sensation.

For peer review only

Study Outcome Measures

The following outcomes will assess the feasibility of a phase-III randomised controlled trial of nVNS to reduce ileus:

- Proportion of eligible patients identified from screening logs
- Number of eligible patients randomised (per month) and reasons for approach failure
- Adequacy of participant blinding according to a blinding poll (Bang Blinding Index) (17)
- Compliance to the pre- and post-operative stimulation schedules according to participant-reported diaries (maximum of 10 stimulations before and after surgery)
- Incidence of complications and serious complications within 30 days
- Proportion of missing clinical outcome data, withdrawals, and loss to follow-up

Progression will be determined using the parameters outlined in Table 2. “Progress” outcomes will build a case for progression to a phase-III trial. “Adjust” outcomes will build a case for progression after appropriate amendments have been made. “Stop” outcomes will contraindicate a case for progression, unless modifiable barriers are identified and further assessments of feasibility are undertaken. The predominance of such outcomes will be considered by the research team in the decision-making process.

Clinical outcomes will be collected to explore variability in candidate measures including: number of postoperative days to first flatus, first stool, first oral intake, hospital discharge, and GI-2 (composite outcome of passage of stool and oral tolerance) (18).

Participant timeline

The timing of study procedures, clinical assessments, and collection points are summarised in Table 3. Participants will attend a pre-intervention study visit, where a trained investigator will provide an interactive tutorial on how to self-administer the device. This will provide an opportunity to practice self-administration under direct supervision. Allocation of study devices will take place at two timepoints. The first will take place at the pre-intervention study visit,

For peer review only

where devices will be provided for use in the community. Upon admission to hospital, these will be collected and switched with the post-operative device. Throughout the stimulation schedule, participants will record their compliance to the device schedule using stimulation diaries. Gastrointestinal function will be recorded daily, as reported by patients. On the last stimulation day, participants will complete a poll to explore the performance of blinding before and after surgery. Follow up data (including the occurrence of complications and serious complications) will be collected 30 days after surgery to capture events which may occur during the index hospital admission or after discharge.

Sample Size

As a feasibility study, no formal power calculation has been performed. In this four-arm parallel-group design, 35 participants per arm will allow robust estimates of variability in continuous clinical outcomes and 70 patients randomised to pre- and post- surgery groups will allow robust estimates of recruitment parameters (19). The total sample size of 140 allows robust exploration of both clinical and recruitment-related objectives.

Recruitment

Participants will be approached with written information in the outpatient departments of St. James's University Hospital and Bradford Royal Infirmary. Research staff will be available to discuss the study over the phone, as well as opportunities to meet in person during patients' routine schedule of pre-assessment and anaesthetic review appointments.

Randomisation & Allocation

Following confirmation of written informed consent, participants will be randomised to one of four intervention arms. The randomisation list will be prepared by an independent statistician using block randomisation with a variable block size. This will be stratified for type of colorectal resection (right- and left-sided) and recruitment centre. Randomisation will be facilitated centrally using an online 24-hour service and will be performed by study investigators.

For peer review only

Blinding

Participants will be blinded to the study intervention before and after surgery. This is made possible through the use of a sham stimulator device which is identical in appearance, weight, and user-interface, as well as being designed to provide a perceptible sub-therapeutic stimulation. All devices will be switched irrespective of whether the type of device (active or sham) remains constant throughout the pre- and post-operative schedules. Investigators and clinical outcome assessors will be unblinded to the intervention. Since outcomes relating to gastrointestinal function are patient-reported, the risk of assessor bias is considered to be low.

Data Collection & Management

All trial data will be prospectively entered on to case report forms by dedicated research staff (a clinical research fellow and research nurses). Physical data will be stored in a restricted-access research unit, accessible only to the research team. All electronic data will be stored in pseudo-anonymised format at University of Leeds according to sponsor requirements. As a feasibility study, a data monitoring committee is not required.

Statistical Analysis Plan

All analyses will follow a predetermined statistical analysis plan (20) and will be overseen by a qualified medical statistician. Recruitment, baseline characteristics, compliance and outcome data will be presented descriptively as rates (categorical) and means (continuous) with 95 per cent confidence intervals. No formal statistical hypothesis testing across randomised groups will be conducted. Groups may be collapsed to pre- and post- surgery stimulus vs sham and reported descriptively. Blinding will be analysed according to the Bang Blinding Index, with an index of >0.2 representing unblinding, $-0.2< x < 0.2$ representing random guesses, and < -0.2 representing opposite guessing (also considered a source of unblinding) (17). All analyses will be performed for intention-to-treat (treatment as randomised) and per-protocol populations. Possible reasons for exclusion from the per-protocol population include non-compliance to the device schedule (defined as any deviation from the five-day

For peer review only - http://bmjopen.bmj.com/site/about/guidelines.xhtml

pre- and post-operative stimulation schedule), conversion from laparoscopic to open surgery, formation of unplanned stoma, return to theatre prior to the return of bowel function, and randomisation errors. The trial will be reported according to CONSORT (21).

Patient and public involvement

As a self-administered device, patient and public involvement is essential at each stage of the study. The design of this feasibility trial was informed through a patient focus group in collaboration with a study-specific patient representative. A patient advisory group comprising of 4-6 patients with experience of the intervention will be convened on six occasions across three years (including one year prior to study recruitment and two years during recruitment). The programme of discussion will be determined by the group, but will include issues of patient approach, intervention burden, device training, and design of study materials.

Dissemination

Feasibility, mechanistic, and qualitative findings will be disseminated to professional and patient groups via peer-reviewed publications, academic conferences, social-media channels, and stakeholder engagement activities.

Trial Sub-studies

Participants will be invited to take part in the following optional sub-studies:

- *Inflammatory response sub-study*

  The inflammatory response sub-study will explore the effect of nVNS on measures of systemic and peritoneal inflammation. This will explore differences in efficacy between randomised arms, which will assist with the design of the final intervention. Recruitment to this sub-study will take place at the lead site only (St. James's University Hospital). Additional eligibility criteria will apply, as outlined in Table 4. Blood draws will be performed at baseline, 2 hours, 24 hours, and 48 hours after the start of

For peer review only

surgery. Peritoneal lavage fluid will also be collected at the start and end of surgery. Lavages will be performed by instilling 20ml of sterile 0.9% NaCl onto the small bowel using a foley catheter and bladder tip syringe, followed by collection of 5-10ml of fluid after 30 seconds, as described previously (22). Serum (systemic) and fluid (peritoneal) levels of tumour necrosis factor (TNF) alpha, interleukin (IL) 1-beta, and IL-6 will be analysed using enzyme-linked immunosorbent assays (ELISA) (23, 24).

– *Bowel motility sub-study*

The motility sub-study will explore the effect of nVNS on global small bowel motility. Differences in efficacy between randomised groups will inform the design of the final intervention. Recruitment will take place at the lead site only. Additional eligibility criteria (Table 4) will apply with a recruitment target of 10 participants per group (total sample: 40 participants). Magnetic resonance (MR) enterography will be performed at baseline and on the third post-operative day in the supine position under breath hold conditions whilst a series of motility acquisitions are performed. Oral preparation with a single glass of water will take place 30 minutes prior to the scan. These will be registered with dedicated software and a quantitative motility score generated to depict bowel wall motion (25).

– *Qualitative sub-study*

A qualitative sub-study will explore participants' acceptability of the trial recruitment process, intervention, their experiences of self-administration, and any challenges with device blinding. Recruitment will take place at the lead site with a target sample of 5 participants per group (total sample: 20 participants). In addition, 5 patients who declined participation in the study will be recruited to explore barriers to study enrolment. A maximum variation approach to sampling will be used to ensure that a wide variety of perspectives are captured, account for age, sex, and indication for surgery). Semi-structured interviews will be conducted after hospital discharge in

For peer review only

hospital or in the community. Questions will be informed by a topic guide and will be recorded in preparation for transcription. A thematic framework approach will be used for analysis, comprising five key stages: I) familiarisation with data; II) identifying the thematic framework; III) indexing; IV) charting; and V) mapping and interpreting (26). The thematic analysis will be modified in light of new data and a process of constant comparison will be used to examine across themes and cases. Qualitative data will be managed in NVivo® 11 (Melbourne, Australia).

For peer review only

Discussion

Although Ileus is a common problem after surgery, limited progress has been made to curtail its impact on patients and healthcare systems in the last 20 years (6). The most successful measures have been the widespread introduction of minimally invasive surgery and the development of evidence-based enhanced recovery protocols. These gains are likely facilitated through inflammatory- and opioid-induced intestinal smooth muscle dysfunction, leading to faster recovery of bowel function (27, 28). The use of vagus nerve stimulation to reduce ileus is a new concept which draws on similar principles. Its efficacy has been demonstrated consistently in pre-clinical studies through activation of a cholinergic anti-inflammatory pathway, leading to a faster restoration of intestinal transit (11). In keeping with this, intra-operative stimulation of the vagus nerve in humans has been shown to attenuate systemic markers of inflammation after major abdominal surgery (14). Taken together, this suggests that the anti-inflammatory mechanism of vagus nerve stimulation is directly translatable to patients and may represent a novel approach to reduce ileus after surgery.

Most previous studies of vagus nerve stimulation have involved surgical access to the neck or abdomen. This is challenging for clinical translation because the procedure is invasive and technically demanding. Non-invasive stimulation over the cervical surface landmark aims to mitigate these challenges and presents a number of possible advantages. One of these is the opportunity for stimulation before and after surgery. This is attractive since some evidence suggests that activation of the cholinergic anti-inflammatory pathway (using prucalopride) is most effective when done pre-operatively (29). Another is the opportunity for stimulation on multiple occasions rather than a single intra-operative episode, which expands the scope for intervention. In this study, a schedule of five days will be used as this represents the time during which most patients are expected to regain bowel function (2). Finally, stimulation can be self-administered by patients which encourages active participation. Previous evidence has shown that active participation is a key motivator during recovery after surgery (30).

For peer review only

In contrast, there are several possible challenges associated with studying and implementing nVNS in this setting. Firstly, patient self-administration requires adequate skill, understanding, and compliance, which may be difficult in the post-operative setting. A dedicated programme of training, including device positioning, aims to mitigate this. Secondly, the non-invasive approach to stimulation is less precise than conventional stimulation since contact with the vagus nerve is not confirmed objectively. This is mitigated through the use of a standard technique (stimulation over the cervical surface landmark), which has been shown to increase heart rate variability, reduce markers of systemic inflammation, and generate vagal somatosensory evoked potentials (indicators of vagal stimulation) previously in healthy volunteers (31-34). Finally, identifying an adequate sham intervention by which to compare nVNS is challenging. In previous healthy volunteer studies, nVNS has been shown to significantly reduce the expression of inflammatory cytokines from baseline but not when using the sham device (32). It is possible that manual instrumentation of the device may be sufficient to elicit some vagal activity, but this is considered minimal and is comparable across both device types. Providing that these challenges can be addressed, nVNS may provide a practical approach to stimulating the vagus nerve in the pre- and post-operative setting.

The present study will inform the feasibility of a phase-III randomised controlled trial of nVNS by addressing key challenges in trial design. This includes compliance to the stimulation schedule in a complex clinical setting where unique barriers may exist. It also includes the adequacy of participant blinding. This is important in studies of electrical nerve stimulation where sham-controls may be associated with statistical improvements in clinical outcomes (35). Some issues of feasibility are beyond the scope of this study. The selection of outcomes for ileus are poorly standardised, with over 50 different outcomes identified from previous literature in the last 20 years (36). To address this, a core outcome set for gastrointestinal recovery is under development by an international collaboration of clinicians and patients (37). This will help to standardise reporting in the future. In addition, the feasibility of implementing nVNS into enhanced recovery after surgery protocols across diverse settings will remain

For peer review only

unclear. The present study explores the feasibility of nVNS in two large teaching hospitals, but further consideration of barriers to implementation will be required.

For peer review only

Ethics approval: Research ethics approval was confirmed by the North East - Tyne & Wear South NHS Research Ethics Committee (19/NE/0217) on 2nd July 2019. Written, informed consent to participate will be obtained from all participants.

Availability of data and materials: A model consent form is available on request.

Competing interests: No competing interests are declared. The views expressed in this publication are those of the authors and not necessarily those of the NHS, the National Institute for Health Research, Health Education England, or the Department of Health.

Funding: This study is funded by a National Institute for Health Research Doctoral Research Fellowship (S Chapman; DRF-2018-11-049). Study devices are provided through the manufacturer's (electroCore™, Inc) Investigator Initiated Trial Programme.

Contributions: SJC and DJ conceptualised the study and all authors (SJC, MN, CJCM, DT, DDS, and DJ) contributed to its design. SJC prepared the protocol draft, which was reviewed and approved by all authors (SJC, MN, CJCM, DT, DDS, and DJ). The Clinical Trials Research Unit at University of Leeds oversees randomisation and University of Leeds is the study sponsor. DDS is the statistical guarantor and DJ is the overall study guarantor.

Copyright: The Corresponding Author has the right to grant on behalf of all authors and does grant on behalf of all authors, a worldwide licence to the Publishers and its licensees in perpetuity, in all forms, formats and media (whether known now or created in the future), to i) publish, reproduce, distribute, display and store the Contribution, ii) translate the Contribution into other languages, create adaptations, reprints, include within collections and create summaries, extracts and/or, abstracts of the Contribution, iii) create any other derivative work(s) based on the Contribution, iv) to exploit all subsidiary rights in the Contribution, v) the inclusion of electronic links from the Contribution to third party material where-ever it may be located; and, vi) licence any third party to do any or all of the above.

Acknowledgements: None

For peer review only

References

1. Scarborough JE. Schumacher J. Kent KC. Heise CP. Greenberg CC. Associations of Specific Postoperative Complications With Outcomes After Elective Colon Resection: A Procedure-Targeted Approach Toward Surgical Quality Improvement. JAMA Surg 2017;152:e164681.
2. Yorkshire Surgical Research Collaborative. Multicentre observational study of gastrointestinal recovery after elective colorectal surgery. Colorectal Dis 2018;20:536-544.
3. Tevis SE. Carchman EH. Foley EF. Harms BA. Heise CP. Kennedy GD. Postoperative Ileus-More than Just Prolonged Length of Stay? J Gastrointest Surg. 2015;19:1684-90.
4. Mao H. Milne TGE. O'Grady G. Vather R. Edlin R. Bissett I. Prolonged Postoperative Ileus Significantly Increases the Cost of Inpatient Stay for Patients Undergoing Elective Colorectal Surgery: Results of a Multivariate Analysis of Prospective Data at a Single Institution. Dis Colon Rectum. 2019;62:631-637.
5. Tiernan J, Cook A, Geh I. et al. Use of a modified Delphi approach to develop research priorities for the association of coloproctology of Great Britain and Ireland. Colorectal Dis 2014;16:965-975.
6. Chapman SJ. Pericleous A. Downey C. Jayne DG. Postoperative ileus following major colorectal surgery. Br J Surg 2018;105:797-810.
7. Herbert G. Perry R. Andersen HK. Atkinson C. Penfold C. Lewis SJ, et al. Early enteral nutrition within 24 hours of lower gastrointestinal surgery versus later commencement for length of hospital stay and postoperative complications. Cochrane Database Syst Rev 2019: CD004080.
8. Short V. Herbert G. Perry R. Atkinson C. Ness AR. Penfold C et al. Chewing gum for postoperative recovery of gastrointestinal function. Cochrane Database Syst Rev 2015: CD006506.
9. Kranke P. Jokinen J. Pace NL. Schnabel A. Hollmann MW. Hahnenkamp K. et al. Continuous intravenous perioperative lidocaine infusion for postoperative pain and recovery. Cochrane Database Syst Rev 2015 :CD009642
10. Chapman SJ. Wells CI. Challenges in ileus research. Colorectal Dis 2018;20:639.
11. Matteoli G. Gomez-Pinilla PJ. Nemethova. A. Di Giovangiulio M. Cailotto C. van Bree SH. et al. A distinct vagal anti-inflammatory pathway modulates intestinal muscularis resident macrophages independent of the spleen. Gut 2014;63:938–948.
12. de Jonge WJ. van der Zanden EP. The FO. Bijlsma MF. van Westerloo DJ. et al. Stimulation of the vagus nerve attenuates macrophage activation by activating the Jak2-STAT3 signalling pathway. Nat Immunol 2005;6:844-851.
13. Bonaz B. Sinniger V. Pellissier S. The Vagus Nerve in the Neuro-Immune Axis: Implications in the Pathology of the Gastrointestinal Tract. Front Immunol 2017;8:1452.

For peer review only

14. Stakenborg N. Wolthuis AM. Gomez-Pinilla PJ. Farro G. Di Giovangiulio M. Bosmans G. et al. Abdominal vagus nerve stimulation as a new therapeutic approach to prevent postoperative ileus. Neurogastro & Motility. 2017;29:e13075.
15. Hong G. Pintea B. Lingohr P. Coch C. Randau T. Schaefer N. et al. Effect of transcutaneous vagus nerve stimulation on muscle activity in the gastrointestinal tract (transVaGa): a prospective clinical trial. International J of Colorectal Dis 2019;34:417–422.
16. Chan AW. Tetzlaff JM. Altman DG, et al. SPIRIT 2013 statement: defining standard protocol items for clinical trials. Ann Intern Med. 2013;158:200-7.
17. Bang H. Ni L. Davis CE. Assessment of blinding in clinical trials. Control Clin Trials 2004;25:143-156.
18. van Bree SH. Bemelman WA. Hollmann MW. Zwinderman AH. Matteoli G. El Temna S. et al. Identification of clinical outcome measures for recovery of gastrointestinal motility in postoperative ileus. Ann Surg 2014;259;708-714.
19. Teare MD. Dimairo M. Shephard N. Hayman A. Whitehead A. Walters SJ. Sample size requirements to estimate key design parameters from external pilot randomised controlled trials: a simulation study. Trials 2014;15:264.
20. Gamble C. Krishan A. Stocken D. Guidelines for the Content of Statistical Analysis Plans in Clinical Trials. JAMA 2017;318:2337-2343.
21. Moher D. Hopewell S. Schulz KF. CONSORT 2010 explanation and elaboration: updated guidelines for reporting parallel group randomised trials. BMJ 2010;340:c869
22. The FO. Bennink RJ. Ankum WM. et al. Intestinal handling-induced mast cell activation and inflammation in human postoperative ileus. Gut 2008;57:33-40.
23. Lange T. Dimitrov S. Born J. Effect of sleep and circadian rhythm on the human immune system. Ann N Y Acad Sci 210;1193:48-59.
24. Baigrie J. Lamont PM. Kwiatkowski D. Dallman MJ. Morris PJ. Systemic cytokine response after major surgery. Br J Surgery 1992;79:757-60.
25. Menys A. Taylor SA. Emmanuel A. Ahmed A. Plumb AA. Odille F. Alam A. Halligan S. Atkinson D. Global small bowel motility: assessment with dynamic MR imaging. Radiology. 2013;269:443-50.
26. Ritchie J. Lewis J. McNaughton Nicholls C. Ormston R. Qualitative Research Practice: A Guide for Social Science Students and Researchers 2nd Ed. Sage. London. 2014
27. Schwenk W. Jacobi C. Mansmann U. Böhm B. Müller JM. Inflammatory response after laparoscopic and conventional colorectal resections - results of a prospective randomized trial. Langenbecks Arch Surg 2000;385:2-9.
28. Venara A. Duchalais E. Dariel A. Aubert P. Durand T. Meurette G. Rolli-Derkinderen M. Hamy A. Neunlist M. Anti-inflammatory Effects of Enhanced Recovery Programs on Early-Stage Colorectal Cancer Surgery. World J Surg 2018;42:953-964.

For peer review only

29. Stakenborg N. Labeeuw E. Gomez-Pinilla PJ. De Schepper S. Aerts R. et al. Preoperative administration of the 5-HT4 receptor agonist prucalopride reduces intestinal inflammation and shortens postoperative ileus via cholinergic enteric neurons. Gut 2019;68:1406-1416.
30. Chapman SJ. Czoski Murray CJ. Lonsdale MDS. Boyes S. Tiernan JP. et al. Information needs for recovery after colorectal surgery: Patient focus group study. *Colorectal Dis* 2020 (In Press).
31. Brock C. Brock B. Aziz Q. Møller HJ. Pfeiffer Jensen M. Drewes AM. Farmer AD. Transcutaneous cervical vagal nerve stimulation modulates cardiac vagal tone and tumor necrosis factor-alpha. Neurogastroenterol Motil 2017;29. doi: 10.1111/nmo.12999.
32. Lerman I. Hauger R. Sorkin L. Proudfoot J. Davis B. et al. Noninvasive Transcutaneous Vagus Nerve Stimulation Decreases Whole Blood Culture-Derived Cytokines and Chemokines: A Randomized, Blinded, Healthy Control Pilot Trial. Neuromodulation 2016;19:283-90.
33. Vecchio E. Bassez I. Ricci K. Tassorelli C. Liebler E. et al. Effect of Non-invasive Vagus Nerve Stimulation on Resting-State Electroencephalography and Laser-Evoked Potentials in Migraine Patients: Mechanistic Insights. Front Hum Neurosci 2018;13;12:366.
34. Nonis R. D'Ostilio K. Schoenen J. Magis D. Evidence of activation of vagal afferents by non-invasive vagus nerve stimulation: An electrophysiological study in healthy volunteers. *Cephalgia* 2017;37:1285-1293.
35. Tan K. Wells CI. Dinning P. Bissett IP. O'Grady G. Placebo Response Rates in Electrical Nerve Stimulation Trials for Fecal Incontinence and Constipation: A Systematic Review and Meta-Analysis. Neuromodulation 2019. doi: 10.1111/ner.13092. [Epub ahead of print]
36. Chapman SJ. Thorpe G. Vallance AE. Harji DP. Lee MJ. et al. Systematic review of definitions and outcome measures for return of bowel function after gastrointestinal surgery. BJS Open 2018;3:1-10.
37. Chapman SJ. Lee MJ. Blackwell S. Arnott R. Ten Broek RPG. et al. Establishing core outcome sets for gastrointestinal recovery in studies of postoperative ileus and small bowel obstruction: protocol for a nested methodological study. Colorectal Dis. 2019. doi: 10.1111/codi.14899. [Epub ahead of print]

For peer review only

2

Table 1 – Eligible Procedures

**Right sided resection**	**Left sided resection**
Ileocolic/caecal resection	Extended left hemicolectomy
Right hemicolectomy	Left hemicolectomy
Extended right hemicolectomy	Sigmoid colectomy
Transverse colectomy	Rectosigmoid colectomy
**Miscellaneous (side grouped accordingly)**	Anterior resection*
Other segmental colonic resection	

For peer review only

Table 2 – Progression criteria

Criteria	Stop	Adjust	Progress
Proportion of eligible patients identified from screening logs	<10%	10-20%	>20%
Number of eligible patients randomised over 24 months (site: SJUH)	≤2 per month	3-4 per month	≥5 per month
Number of eligible patients randomised over 18 months (site: BRI)	<1 per month	1-2 per month	≥3 per month
Adequacy of participant blinding (according to Bang Blinding Index)	Index < -0.5<br>Or Index > 0.5	Index -0.2 to -0.5<br>Or Index 0.2 to 0.5	Index 0 to -0.19<br>Or Index 0 to 0.19
Average compliance to the study treatment schedule *	<10/20 stimulations across 10 days	10-15/20 stimulations across 10 days	≥16/20 stimulations across 10 days
Proportion of missing clinical outcome data	>40%	15-30%	<15%
Proportion of randomised patients lost to follow up	>40%	15-30%	<15%
Incidence of complications or serious complications	>20% increase in complications	5-20% increase in complications	<5% increase in complications

BRI: Bradford Royal Infirmary, Bradford; SJUH: St. James's University Hospital, Leeds

For peer review only

Table 3 – Schedule of Events

	Events	Baseline	Preop study visit	5 days before surgery	Day of surgery (pre-op)	Day of surgery (Intraop)	POD 1	POD 2	POD 3	POD 4	POD 5	POD 6-10	After discharge	30-day phone F/U
**Study Procedures**	Trial consent	✓												
	Device training		✓											
	Device distribution		✓		✓									
	Self-administered stimulation			✓			✓	✓	✓	✓	✓			
	Stimulation diary			✓			✓	✓	✓	✓	✓			
	Blinding poll										✓			
**Data collection**	Eligibility CRF	✓												
	Baseline CRF	✓												
	Operative CRF					✓								
	Gastrointestinal Function CRF						✓	✓	✓	✓	✓	✓		
	Follow-up CRF													✓
**Sub-studies***	*Optional sub-study consents*	✓												
	Inflammatory response study<br>Blood only: ✓; Blood and fluid ✓✓				✓	✓✓	✓		✓					
	Small bowel motility study (MRI)		✓						✓					
	Qualitative sub-study (participant or non-participant interview)												✓	

**Sub-studies are optional and undertaken at the lead site only*
CRF: Case Report Form; F/U: follow up; MRI: magnetic resonance imaging; POD: postoperative day

Table 4 – Sub-study additional exclusion criteria

**Inflammatory response sub-study**
• Regular use of non-steroidal anti-inflammatory drugs in the preceding 7 days<br>• Medical condition requiring oral or injectable steroids<br>• Neoadjuvant chemoradiotherapy within 12 months prior to surgery
**Bowel motility sub-study**
• Indwelling pacemaker or cardiac defibrillator device<br>• Non-MR compatible metallic implants, prostheses, neurosurgical clips, indwelling stimulator or pump devices, which would preclude safe MRI<br>• Claustrophobia<br>• Metallic foreign bodies in the eyes

MRI: Magnetic Resonance Imaging

For peer review only

Figure 1 – Study flow chart

For peer review only

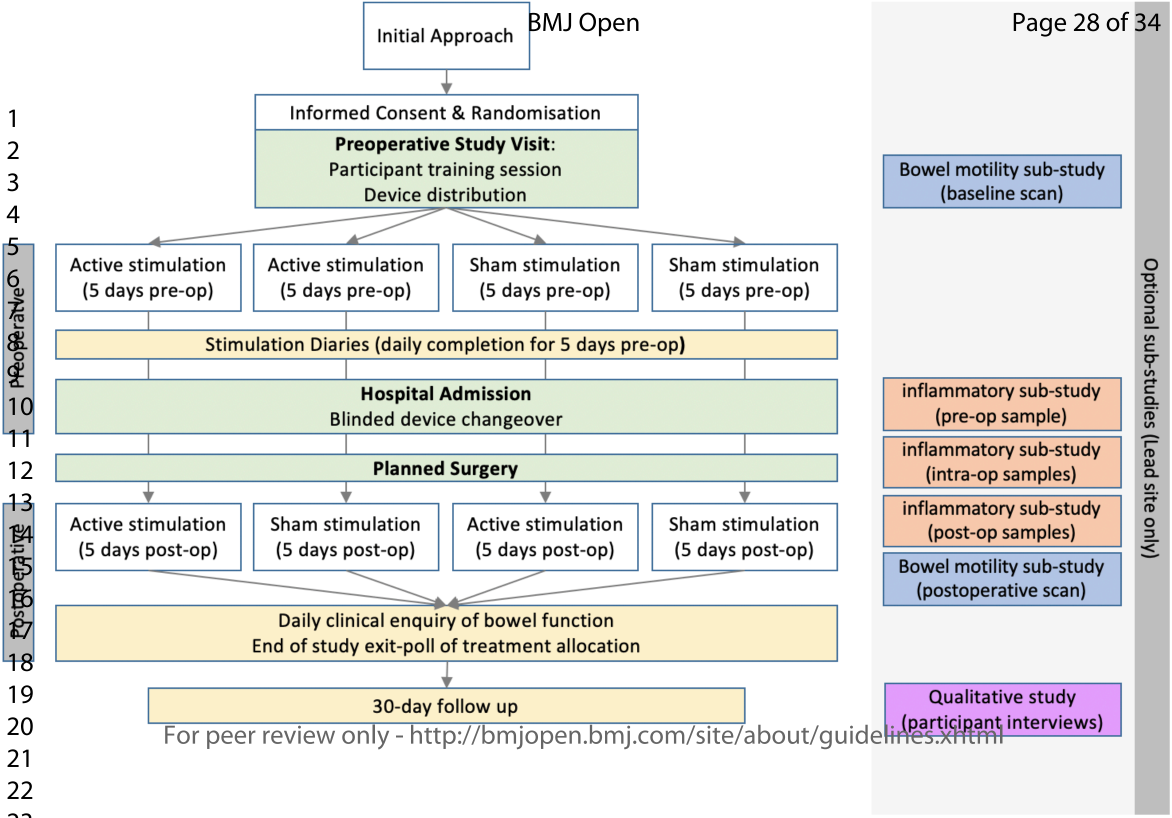
Initial Approach
Informed Consent & Randomisation
Preoperative Study Visit:
Participant training session
Device distribution
Preoperative
Active stimulation (5 days pre-op)
Active stimulation (5 days pre-op)
Sham stimulation (5 days pre-op)
Sham stimulation (5 days pre-op)
Stimulation Diaries (daily completion for 5 days pre-op)
Hospital Admission
Blinded device changeover
Planned Surgery
Postoperative
Active stimulation (5 days post-op)
Sham stimulation (5 days post-op)
Active stimulation (5 days post-op)
Sham stimulation (5 days post-op)
Daily clinical enquiry of bowel function
End of study exit-poll of treatment allocation
30-day follow up
Bowel motility sub-study (baseline scan)
inflammatory sub-study (pre-op sample)
inflammatory sub-study (intra-op samples)
inflammatory sub-study (post-op samples)
Bowel motility sub-study (postoperative scan)
Qualitative study (participant interviews)
Optional sub-studies (Lead site only)

Supplementary File

Study Status

The study first began recruitment on 2nd January 2020 and is expected to complete by 31st August 2021. The current protocol version is v1.2 (13th February 2020).

Auditing

The conduct of the trial will be audited monthly by the principal investigator. In line with Good Clinical Practice, a study site folder will be held at each participating recruitment site. This will contain the current study protocol, delegation log outlining responsibilities of all research team members, and all study correspondence. Data relating to recruitment will be uploaded to the NIHR Central Portfolio Management System on a monthly basis.

Adverse Events

The reporting of adverse events will be done in line with Good Clinical Practice. All serious adverse events will be reported to the sponsor within 24 hours of research staff becoming aware of the event. Serious adverse events that are considered to be unexpected and related to the study will be escalated to the research ethics committee.

Study Amendments

If amendments to the study protocol or documents are required, this will be done and communicated in line with Health Research Authority guidelines. All amendments will be categorised and submitted for review by the Research Ethics Committee, as appropriate.

Dissemination Plan

The findings of the study and sub-studies will be disseminated at academic conferences and in peer-reviewed manuscripts. The results will also be summarised in plain English visual abstracts and disseminated to research participants and the public.

For peer review only

Study Sponsor

The sponsor for this study is University of Leeds

Recruitment & Protocol Materials

Study recruitment and protocol materials are available upon request from the corresponding author.

For peer review only

SPIRIT

STANDARD PROTOCOL ITEMS: RECOMMENDATIONS FOR INTERVENTIONAL TRIALS

SPIRIT 2013 Checklist: Recommended items to address in a clinical trial protocol and related documents*

Section/item	Item No	Description
**Administrative information**		
Title	1	Descriptive title identifying the study design, population, interventions, and, if applicable, trial acronym **(Page 1)**
Trial registration	2a	Trial identifier and registry name. If not yet registered, name of intended registry **(Page 5)**
	2b	All items from the World Health Organization Trial Registration Data Set **(Page 5)**
Protocol version	3	Date and version identifier **(Page 5)**
Funding	4	Sources and types of financial, material, and other support **(Page 1)**
Roles and responsibilities	5a	Names, affiliations, and roles of protocol contributors **(Page 1)**
	5b	Name and contact information for the trial sponsor **(Suppl. File 1)**
	5c	Role of study sponsor and funders, if any, in study design; collection, management, analysis, and interpretation of data; writing of the report; and the decision to submit the report for publication, including whether they will have ultimate authority over any of these activities **(Page 1)**
	5d	Composition, roles, and responsibilities of the coordinating centre, steering committee, endpoint adjudication committee, data management team, and other individuals overseeing the trial, if applicable (see Item 21a for data monitoring committee) **(N/A)**
**Introduction**		
Background and rationale	6a	Description of research question and justification for undertaking the trial, including summary of relevant studies (published and unpublished) examining benefits and harms for each intervention **(Page 3-4)**
	6b	Explanation for choice of comparators **(Page 3-4)**
Objectives	7	Specific objectives or hypotheses **(Page 5)**

For peer review only

Trial design	8	Description of trial design including type of trial (eg, parallel group, crossover, factorial, single group), allocation ratio, and framework (eg, superiority, equivalence, noninferiority, exploratory) **(Page 5)**

Methods: Participants, interventions, and outcomes

Study setting	9	Description of study settings (eg, community clinic, academic hospital) and list of countries where data will be collected. Reference to where list of study sites can be obtained **(Page 6)**
Eligibility criteria	10	Inclusion and exclusion criteria for participants. If applicable, eligibility criteria for study centres and individuals who will perform the interventions (eg, surgeons, psychotherapists) **(Page 6)**
Interventions	11a	Interventions for each group with sufficient detail to allow replication, including how and when they will be administered **(Page 7)**
	11b	Criteria for discontinuing or modifying allocated interventions for a given trial participant (eg, drug dose change in response to harms, participant request, or improving/worsening disease) **(N/A)**
	11c	Strategies to improve adherence to intervention protocols, and any procedures for monitoring adherence (eg, drug tablet return, laboratory tests) **(N/A)**
	11d	Relevant concomitant care and interventions that are permitted or prohibited during the trial **(Page 7)**
Outcomes	12	Primary, secondary, and other outcomes, including the specific measurement variable (eg, systolic blood pressure), analysis metric (eg, change from baseline, final value, time to event), method of aggregation (eg, median, proportion), and time point for each outcome. Explanation of the clinical relevance of chosen efficacy and harm outcomes is strongly recommended **(Page 7-8)**
Participant timeline	13	Time schedule of enrolment, interventions (including any run-ins and washouts), assessments, and visits for participants. A schematic diagram is highly recommended (see Figure) **(Page 8-9)**
Sample size	14	Estimated number of participants needed to achieve study objectives and how it was determined, including clinical and statistical assumptions supporting any sample size calculations **(Page 9)**
Recruitment	15	Strategies for achieving adequate participant enrolment to reach target sample size **(Page 9)**

Methods: Assignment of interventions (for controlled trials)

Allocation:

Sequence generation	16a	Method of generating the allocation sequence (eg, computer-generated random numbers), and list of any factors for stratification. To reduce predictability of a random sequence, details of any planned restriction (eg, blocking) should be provided in a separate document that is unavailable to those who enrol participants or assign interventions **(Page 9)**
Allocation concealment mechanism	16b	Mechanism of implementing the allocation sequence (eg, central telephone; sequentially numbered, opaque, sealed envelopes), describing any steps to conceal the sequence until interventions are assigned **(Page 9)**
Implementation	16c	Who will generate the allocation sequence, who will enrol participants, and who will assign participants to interventions **(Page 9)**
Blinding (masking)	17a	Who will be blinded after assignment to interventions (eg, trial participants, care providers, outcome assessors, data analysts), and how **(Page 9-10)**
	17b	If blinded, circumstances under which unblinding is permissible, and procedure for revealing a participant's allocated intervention during the trial **(N/A)**

Methods: Data collection, management, and analysis

Data collection methods	18a	Plans for assessment and collection of outcome, baseline, and other trial data, including any related processes to promote data quality (eg, duplicate measurements, training of assessors) and a description of study instruments (eg, questionnaires, laboratory tests) along with their reliability and validity, if known. Reference to where data collection forms can be found, if not in the protocol **(Page 10)**
	18b	Plans to promote participant retention and complete follow-up, including list of any outcome data to be collected for participants who discontinue or deviate from intervention protocols
Data management	19	Plans for data entry, coding, security, and storage, including any related processes to promote data quality (eg, double data entry; range checks for data values). Reference to where details of data management procedures can be found, if not in protocol **(Page 10)**
Statistical methods	20a	Statistical methods for analysing primary and secondary outcomes. Reference to where other details of the statistical analysis plan can be found, if not in the protocol **(Page 10)**
	20b	Methods for any additional analyses (eg, subgroup and adjusted analyses) **(Page 10)**

For peer review only - http://bmjopen.bmj.com/site/about/guidelines.xhtml

	20c	Definition of analysis population relating to protocol non-adherence (eg, as randomised analysis), and any statistical methods to handle missing data (eg, multiple imputation) **(Page 10)**
**Methods: Monitoring**		
Data monitoring	21a	Composition of data monitoring committee (DMC); summary of its role and reporting structure; statement of whether it is independent from the sponsor and competing interests; and reference to where further details about its charter can be found, if not in the protocol. Alternatively, explanation of why a DMC is not needed **(Suppl. File 1)**
	21b	Description of any interim analyses and stopping guidelines, including who will have access to these interim results and make the final decision to terminate the trial **(Suppl. File 1)**
Harms	22	Plans for collecting, assessing, reporting, and managing solicited and spontaneously reported adverse events and other unintended effects of trial interventions or trial conduct **(Suppl. File 1)**
Auditing	23	Frequency and procedures for auditing trial conduct, if any, and whether the process will be independent from investigators and the sponsor **(Suppl. File 1)**
**Ethics and dissemination**		
Research ethics approval	24	Plans for seeking research ethics committee/institutional review board (REC/IRB) approval **(Page 5)**
Protocol amendments	25	Plans for communicating important protocol modifications (eg, changes to eligibility criteria, outcomes, analyses) to relevant parties (eg, investigators, REC/IRBs, trial participants, trial registries, journals, regulators) **(Suppl. File 1)**
Consent or assent	26a	Who will obtain informed consent or assent from potential trial participants or authorised surrogates, and how (see Item 32) **(Page 9)**
	26b	Additional consent provisions for collection and use of participant data and biological specimens in ancillary studies **(Page 11-12)**
Confidentiality	27	How personal information about potential and enrolled participants will be collected, shared, and maintained in order to protect confidentiality before, during, and after the trial **(Page 10)**
Declaration of interests	28	Financial and other competing interests for principal investigators for the overall trial and each study site **(Page 1)**
Access to data	29	Statement of who will have access to the final trial dataset, and disclosure of contractual agreements that limit such access for investigators **(N/A)**
Ancillary and post-trial care	30	Provisions, if any, for ancillary and post-trial care, and for compensation to those who suffer harm from trial participation **(N/A)**

For peer review only - http://bmjopen.bmj.com/site/about/guidelines.xhtml

Dissemination policy	31a	Plans for investigators and sponsor to communicate trial results to participants, healthcare professionals, the public, and other relevant groups (eg, via publication, reporting in results databases, or other data sharing arrangements), including any publication restrictions **(Suppl. File 1)**
	31b	Authorship eligibility guidelines and any intended use of professional writers **(Suppl. File 1)**
	31c	Plans, if any, for granting public access to the full protocol, participant-level dataset, and statistical code **(Suppl. File 1)**
**Appendices**		
Informed consent materials	32	Model consent form and other related documentation given to participants and authorised surrogates **(Suppl. File 1)**
Biological specimens	33	Plans for collection, laboratory evaluation, and storage of biological specimens for genetic or molecular analysis in the current trial and for future use in ancillary studies, if applicable **(Pages 11-12)**

*It is strongly recommended that this checklist be read in conjunction with the SPIRIT 2013 Explanation & Elaboration for important clarification on the items. Amendments to the protocol should be tracked and dated. The SPIRIT checklist is copyrighted by the SPIRIT Group under the Creative Commons "Attribution-NonCommercial-NoDerivs 3.0 Unported" license.

For peer review only

BMJ Open

Non-invasive, vagus nerve stimulation to reduce ileus after colorectal surgery: Protocol for a feasibility trial with nested mechanistic studies

Journal:	*BMJ Open*
Manuscript ID	bmjopen-2020-046313.R3
Article Type:	Protocol
Date Submitted by the Author:	28-Apr-2021
Complete List of Authors:	Chapman, Stephen; University of Leeds, Leeds Institute of Medical Research<br>Naylor, Maureen; West Riding of Yorkshire Ileostomy Association<br>Czoski Murray, Carolyn; University of Leeds, Leeds Institute of Health Sciences<br>Tolan, Damian; Leeds Teaching Hospitals NHS Trust<br>Stocken, Deborah; University of Leeds, Leeds Institute of Clinical Trials Research<br>Jayne, David; University of Leeds, Leeds Institute of Medical Research
**Primary Subject Heading**:	Surgery
Secondary Subject Heading:	Gastroenterology and hepatology, Qualitative research, Research methods
Keywords:	SURGERY, GASTROENTEROLOGY, NEUROPHYSIOLOGY

SCHOLARONE™
Manuscripts

I, the Submitting Author has the right to grant and does grant on behalf of all authors of the Work (as defined in the below author licence), an exclusive licence and/or a non-exclusive licence for contributions from authors who are: i) UK Crown employees; ii) where BMJ has agreed a CC-BY licence shall apply, and/or iii) in accordance with the terms applicable for US Federal Government officers or employees acting as part of their official duties; on a worldwide, perpetual, irrevocable, royalty-free basis to BMJ Publishing Group Ltd ("BMJ") its licensees and where the relevant Journal is co-owned by BMJ to the co-owners of the Journal, to publish the Work in this journal and any other BMJ products and to exploit all rights, as set out in our licence.

The Submitting Author accepts and understands that any supply made under these terms is made by BMJ to the Submitting Author unless you are acting as an employee on behalf of your employer or a postgraduate student of an affiliated institution which is paying any applicable article publishing charge ("APC") for Open Access articles. Where the Submitting Author wishes to make the Work available on an Open Access basis (and intends to pay the relevant APC), the terms of reuse of such Open Access shall be governed by a Creative Commons licence – details of these licences and which Creative Commons licence will apply to this Work are set out in our licence referred to above.

Other than as permitted in any relevant BMJ Author's Self Archiving Policies, I confirm this Work has not been accepted for publication elsewhere, is not being considered for publication elsewhere and does not duplicate material already published. I confirm all authors consent to publication of this Work and authorise the granting of this licence.

For peer review only

Non-invasive, vagus nerve stimulation to reduce ileus after colorectal surgery: Protocol for a feasibility trial with nested mechanistic studies

Stephen J Chapman[1]. Maureen Naylor [2]. Carolyn J Czoski Murray[3]. Damian Tolan[4]. Deborah D Stocken[5]. David Jayne[6].

1. Leeds Institute of Medical Research, University of Leeds, UK; S.Chapman@leeds.ac.uk
2. West Riding of Yorkshire Ileostomy Association; m.naylor70@btinternet.com
3. Leeds Institute of Health Sciences, University of Leeds, UK; C.J.CzoskiMurray@leeds.ac.uk
4. Leeds Teaching Hospitals NHS Trust, Leeds, UK; Damian.Tolan@nhs.net
5. Leeds Institute for Clinical Trials Research, University of Leeds, UK: D.D.Stocken@leeds.ac.uk
6. Leeds Institute of Medical Research, University of Leeds, UK; D.G.Jayne@leeds.ac.uk

Corresponding Author

Stephen J Chapman, Room 7.16 Clinical Sciences Building, St. James's University Hospital, University of Leeds, LS2 9JT, United Kingdom

Email: S.Chapman@leeds.ac.uk; Twitter: @SJ_Chapman; ORCID: 0000-0003-2413-5690

Running title: Non-invasive vagus nerve stimulation to reduce ileus

Category: Protocol

Main body word count: 3,308

Keywords: ileus, colorectal surgery, vagus nerve stimulation, Feasibility RCT

For peer review only

Abstract

Introduction: Ileus is a common and distressing condition characterised by gut dysfunction after surgery. Whilst a number of interventions have aimed to curtail its impact on patients and healthcare systems, ileus is still an unmet challenge. Electrical stimulation of the vagus nerve is a promising new treatment due to its role in modulating the neuro-immune axis through a novel anti-inflammatory reflex. The protocol for a feasibility study of non-invasive vagus nerve stimulation (nVNS), and a programme of mechanistic and qualitative studies, is described.

Methods and Analysis: This is a participant-blinded, parallel-group, randomised, sham-controlled feasibility trial (IDEAL Stage 2b) of self-administered nVNS. One hundred and forty patients planned for elective, minimally-invasive, colorectal surgery, will be randomised to four schedules of nVNS before and after surgery. Feasibility outcomes include assessments of recruitment and attrition, adequacy of blinding, and compliance to the intervention. Clinical outcomes include bowel function and length of hospital stay. A series of mechanistic sub-studies exploring the impact of nVNS on inflammation and bowel motility will inform the design of the final stimulation schedule. Semi-structured interviews with participants will explore experiences and perceptions of the intervention, whilst interviews with patients who decline participation will explore barriers to recruitment.

Ethics and Dissemination: The protocol has been approved by the Tyne & Wear South NHS Research Ethics Committee (19/NE/0217) on 2nd July 2019. Feasibility, mechanistic, and qualitative findings will be disseminated to national and international partners through peer-reviewed publications, academic conferences, social media channels, and stakeholder engagement activities. The findings will build a case for or against progression to a definitive randomised assessment as well as informing key elements of study design.

Trial Registration: ISRCTN; ISRCTN62033341; Prospectively registered on 11th October 2019; http://www.isrctn.com/ISRCTN62033341.

For peer review only

Strengths and limitations of this study

- Feasibility outcomes will support the development of a definitive, randomised controlled trial, including considerations of recruitment, device compliance, and sham-blinding.
- Qualitative outcomes will explore possible barriers to participation and compliance with the self-administered device before and after colorectal surgery
- The study will not explore the most appropriate outcome to be used in a definitive randomised controlled trial, but other related work done in parallel will fill this need.

For peer review only

Introduction

Ileus is a distressing condition which occurs in 10-20% of patients undergoing major abdominal surgery (1). It is characterised by painful abdominal distension, persistent vomiting, and delayed bowel function. This usually resolves 2-4 days after surgery, but in some cases, it may persist in excess of 10 days (2). The impact of ileus is extensive. For patients, it increases the length of hospital stay and increases the risk of serious postoperative complications such as pneumonia, surgical site infection, and venous thrombo-embolic events (3). For hospitals, it is economically burdensome and is associated with an increase of 71% in healthcare costs (4). Once considered to be a normal consequence of surgical recovery, the Association of Coloproctology in Great Britain & Ireland now considers ileus to be a research priority (5).

In the last 20 years, a number of interventions to prevent ileus and its clinical sequelae have been explored (6). Strategies to encourage early or sham-feeding (chewing gum) have been studied extensively but evidence for their effectiveness remains contentious (7, 8). New approaches to perioperative management (such as intravenous lidocaine) have been explored but uncertainty relating to efficacy, dose, and timing persist (9). Novel drugs such as mu-opioid receptor antagonists have shown promising results but economic and regulatory barriers have limited their uptake beyond North America. The consensus of existing literature suggests that minimally-invasive surgery and protocol-driven recovery offer the best chances of preventing ileus (10). Uncertainty exists over the pathophysiology of ileus, however, which is problematic for designing clinical interventions that are justified by strong scientific evidence.

Stimulation of the vagus nerve may represent a novel and evidence-based approach to reduce ileus after surgery. In pre-clinical models, electrical stimulation of the vagus nerve accelerates the recovery of gastrointestinal transit. This is facilitated through vagal afferents targeting the hypothalamic-pituitary-adrenal axis and vagal efferents targeting the cholinergic anti-inflammatory pathway, which attenuate inflammatory-mediated gut dysfunction (11-13). Early clinical studies have suggested that this is directly translatable to humans undergoing surgery (14, 15). Challenges exist, however, as to how vagus nerve stimulation can be done safely,

For peer review only

how its efficacy can be optimised, and how patient-acceptability can be ensured. A protocol for a randomised feasibility trial of non-invasive vagus nerve stimulation (nVNS) to reduce ileus, as well as a series of mechanistic and qualitative studies, is described.

For peer review only

Methods

Ethics & Governance

Ethical approval for the study was obtained from North East - Tyne & Wear South Research Ethics Committee (19/NE/0217) on 2nd July 2019. The study was prospectively registered on the ISRCTN registry (ISRCTN62033341; http://www.isrctn.com/ISRCTN62033341) on 11th October 2019 prior to the start of recruitment (Suppl. File 1). The study devices will be provided through the manufacturer's (electroCore™, Inc) Investigator Initiated Trial Programme. The present manuscript is reported according to the Standard Protocol Items Recommendation For Interventional Trials (SPIRIT) 2013 Checklist (Suppl. File 2) (16).

Aims & Objectives

The aim of this study is to determine the feasibility of performing a multicentre, phase-III, randomised-controlled trial (RCT) of nVNS to reduce ileus after colorectal surgery. The following objectives will explore the case for progression to a phase-III trial:

- To estimate the proportion of patients screened who are eligible for approach
- To estimate the number of approached patients who consent to randomisation
- To assess the adequacy of participant blinding to the allocated intervention
- To assess participant compliance to the intervention schedule
- To explore the safety of the intervention schedule
- To estimate the rate of missing outcome data, rate of withdrawal, and loss to follow up

Study Design

This study is a participant-blinded, parallel-group, randomised, sham-controlled feasibility trial (IDEAL Stage 2b) of nVNS to reduce ileus after colorectal surgery (Figure 1). One hundred and forty participants will be recruited across two study sites. Participants will be randomised to four arms (1:1:1:1) according to the following stimulation schedules:

- Pre-operative stimulation and post-operative stimulation
- Pre-operative sham and post-operative stimulation
- Pre-operative stimulation and post-operative sham
- Pre-operative sham and post-operative sham

The estimated trial duration is 24 months and all participants will be followed up for 30 days after the date of surgery.

Study Setting

The intervention will take place within the community (pre-operative stimulation) and in hospital (post-operative stimulation). All participants will undergo surgery at one of two large teaching hospitals (St. James's University Hospital, Leeds; and Bradford Royal Infirmary, Bradford). Both provide elective colorectal cancer services including laparoscopic surgery within a programme of enhanced recovery.

Eligibility Criteria

To be considered eligible for the trial, patients must satisfy all of the following inclusion criteria:

- Aged ≥ 18 years
- Able to provide written informed consent
- Planned to undergo elective, minimally-invasive (laparoscopic or robotic) surgery with a planned anastomosis/no routine plans for a diverting stoma, including one of the procedures listed in Table 1.

Presence of any of the following exclusion criteria will preclude participation in the trial:

- Severe cardiac disease (myocardial infarction in the last 12 months; heart failure with New York Heart Association Scale >2; 2nd or 3rd degree atrioventricular block, permanent atrial fibrillation/flutter, or previous ventricular tachycardia/fibrillation).
- Seizures or recurrent episodes of syncope (>1) in the last five years

For peer review only

- Previous transient ischaemic attack or cerebral vascular accident
- Previous vagotomy at any anatomical location
- Confirmed diagnosis of chronic gastrointestinal inflammatory condition
- Confirmed diagnosis of neuro-endocrine tumour
- Existing gastrointestinal stoma
- Implanted electrical stimulator device (such as cardiac pacemaker or defibrillator
- Structural abnormality of the neck anatomy which may impact on use of the device
- Belonging to a vulnerable population (such as those lacking capacity, prisoners)
- Pregnant, nursing, or thinking of becoming pregnant during the study period

Participants will not be eligible for entry into other interventional studies which aim to optimise surgical recovery or for which the intervention may impact on bowel function.

Intervention

Non-invasive vagus nerve stimulation will be self-administered by participants using the GammaCore® device (electroCore™, LLC, New Jersey, USA). This provides a transcutaneous electrical stimulation comprising of 5 x 5KHz sine-wave complexes delivered over 1ms and repeated at 25Hz (maximum voltage: 24V; maximum output current: 60mA). The surface landmark of the carotid pulse is used to guide positioning of the device over the cervical vagus nerve. Participants will self-administer the device twice daily for five days before and after surgery, with each administration lasting for 2 minutes on either side of the neck. Participants will be instructed to adjust the stimulation setting to the maximum tolerated level. A sham device identical in appearance will be used as a control. This produces a sub-therapeutic stimulation (0.1Hz biphasic direct current) whilst providing a perceptible stimulation sensation.

For peer review only

Study Outcome Measures

The following outcomes will assess the feasibility of a phase-III randomised controlled trial of nVNS to reduce ileus:

- Proportion of eligible patients identified from screening logs
- Number of eligible patients randomised (per month) and reasons for approach failure
- Adequacy of participant blinding according to a blinding poll (Bang Blinding Index) (17)
- Compliance to the pre- and post-operative stimulation schedules according to participant-reported diaries (maximum of 10 stimulations before and after surgery)
- Incidence of complications and serious complications within 30 days
- Proportion of missing clinical outcome data, withdrawals, and loss to follow-up

Progression will be determined using the parameters outlined in Table 2. “Progress” outcomes will build a case for progression to a phase-III trial. “Adjust” outcomes will build a case for progression after appropriate amendments have been made. “Stop” outcomes will contraindicate a case for progression, unless modifiable barriers are identified and further assessments of feasibility are undertaken. The predominance of such outcomes will be considered by the research team in the decision-making process.

Clinical outcomes will be collected to explore variability in candidate measures including: number of postoperative days to first flatus, first stool, first oral intake, hospital discharge, and GI-2 (composite outcome of passage of stool and oral tolerance) (18).

Participant timeline

The timing of study procedures, clinical assessments, and collection points are summarised in Table 3. Participants will attend a pre-intervention study visit, where a trained investigator will provide an interactive tutorial on how to self-administer the device. This will provide an opportunity to practice self-administration under direct supervision. Allocation of study devices will take place at two timepoints. The first will take place at the pre-intervention study visit,

For peer review only

where devices will be provided for use in the community. Upon admission to hospital, these will be collected and switched with the post-operative device. Throughout the stimulation schedule, participants will record their compliance to the device schedule using stimulation diaries. Gastrointestinal function will be recorded daily, as reported by patients. On the last stimulation day, participants will complete a poll to explore the performance of blinding before and after surgery. Follow up data (including the occurrence of complications and serious complications) will be collected 30 days after surgery to capture events which may occur during the index hospital admission or after discharge.

Sample Size

As a feasibility study, no formal power calculation has been performed. In this four-arm parallel-group design, 35 participants per arm will allow robust estimates of variability in continuous clinical outcomes and 70 patients randomised to pre- and post- surgery groups will allow robust estimates of recruitment parameters (19). The total sample size of 140 allows robust exploration of both clinical and recruitment-related objectives.

Recruitment

Participants will be approached with written information in the outpatient departments of St. James's University Hospital and Bradford Royal Infirmary. Research staff will be available to discuss the study over the phone, as well as opportunities to meet in person during patients' routine schedule of pre-assessment and anaesthetic review appointments. Consent for participation in the study will be gathered using the approved consent form (Suppl. File 3).

Randomisation & Allocation

Following confirmation of written informed consent, participants will be randomised to one of four intervention arms. The randomisation list will be prepared by an independent statistician using block randomisation with a variable block size. This will be stratified for type of colorectal

For peer review only

resection (right- and left-sided) and recruitment centre. Randomisation will be facilitated centrally using an online 24-hour service and will be performed by study investigators.

Blinding

Participants will be blinded to the study intervention before and after surgery. This is made possible through the use of a sham stimulator device which is identical in appearance, weight, and user-interface, as well as being designed to provide a perceptible sub-therapeutic stimulation. All devices will be switched irrespective of whether the type of device (active or sham) remains constant throughout the pre- and post-operative schedules. Investigators and clinical outcome assessors will be unblinded to the intervention. Since outcomes relating to gastrointestinal function are patient-reported, the risk of assessor bias is considered to be low.

Data Collection & Management

All trial data will be prospectively entered on to case report forms by dedicated research staff (a clinical research fellow and research nurses). Physical data will be stored in a restricted-access research unit, accessible only to the research team. All electronic data will be stored in pseudo-anonymised format at University of Leeds according to sponsor requirements. As a feasibility study, a data monitoring committee is not required.

Statistical Analysis Plan

All analyses will follow a predetermined statistical analysis plan (20) and will be overseen by a qualified medical statistician. Recruitment, baseline characteristics, compliance and outcome data will be presented descriptively as rates (categorical) and means (continuous) with 95 per cent confidence intervals. No formal statistical hypothesis testing across randomised groups will be conducted. Groups may be collapsed to pre- and post- surgery stimulus vs sham and reported descriptively. Blinding will be analysed according to the Bang Blinding Index, with an index of >0.2 representing unblinding, -0.2< x < 0.2 representing random guesses, and < -0.2 representing opposite guessing (also considered a source of

For peer review only

unblinding) (17). All analyses will be performed for intention-to-treat (treatment as randomised) and per-protocol populations. Possible reasons for exclusion from the per-protocol population include non-compliance to the device schedule (defined as any deviation from the five-day pre- and post-operative stimulation schedule), conversion from laparoscopic to open surgery, formation of unplanned stoma, return to theatre prior to the return of bowel function, and randomisation errors. The trial will be reported according to CONSORT (21).

Patient and public involvement

As a self-administered device, patient and public involvement is essential at each stage of the study. The design of this feasibility trial was informed through a patient focus group in collaboration with a study-specific patient representative. A patient advisory group comprising of 4-6 patients with experience of the intervention will be convened on six occasions across three years (including one year prior to study recruitment and two years during recruitment). The programme of discussion will be determined by the group, but will include issues of patient approach, intervention burden, device training, and design of study materials.

Dissemination

Feasibility, mechanistic, and qualitative findings will be disseminated to professional and patient groups via peer-reviewed publications, academic conferences, social-media channels, and stakeholder engagement activities.

Trial Sub-studies

Participants will be invited to take part in the following optional sub-studies:

– *Inflammatory response sub-study*

The inflammatory response sub-study will explore the effect of nVNS on measures of systemic and peritoneal inflammation. This will explore differences in efficacy between randomised arms, which will assist with the design of the final intervention.

For peer review only

Recruitment to this sub-study will take place at the lead site only (St. James's University Hospital). Additional eligibility criteria will apply, as outlined in Table 4. Blood draws will be performed at baseline, 2 hours, 24 hours, and 48 hours after the start of surgery. Peritoneal lavage fluid will also be collected at the start and end of surgery. Lavages will be performed by instilling 20ml of sterile 0.9% NaCl onto the small bowel using a foley catheter and bladder tip syringe, followed by collection of 5-10ml of fluid after 30 seconds, as described previously (22). Serum (systemic) and fluid (peritoneal) levels of tumour necrosis factor (TNF) alpha, interleukin (IL) 1-beta, and IL-6 will be analysed using enzyme-linked immunosorbent assays (ELISA) (23, 24).

– *Bowel motility sub-study*

The motility sub-study will explore the effect of nVNS on global small bowel motility. Differences in efficacy between randomised groups will inform the design of the final intervention. Recruitment will take place at the lead site only. Additional eligibility criteria (Table 4) will apply with a recruitment target of 10 participants per group (total sample: 40 participants). Magnetic resonance (MR) enterography will be performed at baseline and on the third post-operative day in the supine position under breath hold conditions whilst a series of motility acquisitions are performed. Oral preparation with a single glass of water will take place 30 minutes prior to the scan. These will be registered with dedicated software and a quantitative motility score generated to depict bowel wall motion (25).

– *Qualitative sub-study*

A qualitative sub-study will explore participants' acceptability of the trial recruitment process, intervention, their experiences of self-administration, and any challenges with device blinding. Recruitment will take place at the lead site with a target sample of 5 participants per group (total sample: 20 participants). In addition, 5 patients who declined participation in the study will be recruited to explore barriers to study

For peer review only

enrolment. A maximum variation approach to sampling will be used to ensure that a wide variety of perspectives are captured, account for age, sex, and indication for surgery). Semi-structured interviews will be conducted after hospital discharge in hospital or in the community. Questions will be informed by a topic guide and will be recorded in preparation for transcription. A thematic framework approach will be used for analysis, comprising five key stages: I) familiarisation with data; II) identifying the thematic framework; III) indexing; IV) charting; and V) mapping and interpreting (26). The thematic analysis will be modified in light of new data and a process of constant comparison will be used to examine across themes and cases. Qualitative data will be managed in NVivo® 11 (Melbourne, Australia).

For peer review only

Discussion

Although Ileus is a common problem after surgery, limited progress has been made to curtail its impact on patients and healthcare systems in the last 20 years (6). The most successful measures have been the widespread introduction of minimally invasive surgery and the development of evidence-based enhanced recovery protocols. These gains are likely facilitated through inflammatory- and opioid-induced intestinal smooth muscle dysfunction, leading to faster recovery of bowel function (27, 28). The use of vagus nerve stimulation to reduce ileus is a new concept which draws on similar principles. Its efficacy has been demonstrated consistently in pre-clinical studies through activation of a cholinergic anti-inflammatory pathway, leading to a faster restoration of intestinal transit (11). In keeping with this, intra-operative stimulation of the vagus nerve in humans has been shown to attenuate systemic markers of inflammation after major abdominal surgery (14). Taken together, this suggests that the anti-inflammatory mechanism of vagus nerve stimulation is directly translatable to patients and may represent a novel approach to reduce ileus after surgery.

Most previous studies of vagus nerve stimulation have involved surgical access to the neck or abdomen. This is challenging for clinical translation because the procedure is invasive and technically demanding. Non-invasive stimulation over the cervical surface landmark aims to mitigate these challenges and presents a number of possible advantages. One of these is the opportunity for stimulation before and after surgery. This is attractive since some evidence suggests that activation of the cholinergic anti-inflammatory pathway (using prucalopride) is most effective when done pre-operatively (29). Another is the opportunity for stimulation on multiple occasions rather than a single intra-operative episode, which expands the scope for intervention. In this study, a schedule of five days will be used as this represents the time during which most patients are expected to regain bowel function (2). Finally, stimulation can be self-administered by patients which encourages active participation. Previous evidence has shown that active participation is a key motivator during recovery after surgery (30).

For peer review only

In contrast, there are several possible challenges associated with studying and implementing nVNS in this setting. Firstly, patient self-administration requires adequate skill, understanding, and compliance, which may be difficult in the post-operative setting. A dedicated programme of training, including device positioning, aims to mitigate this. Secondly, the non-invasive approach to stimulation is less precise than conventional stimulation since contact with the vagus nerve is not confirmed objectively. This is mitigated through the use of a standard technique (stimulation over the cervical surface landmark), which has been shown to increase heart rate variability, reduce markers of systemic inflammation, and generate vagal somatosensory evoked potentials (indicators of vagal stimulation) previously in healthy volunteers (31-34). Finally, identifying an adequate sham intervention by which to compare nVNS is challenging. In previous healthy volunteer studies, nVNS has been shown to significantly reduce the expression of inflammatory cytokines from baseline but not when using the sham device (32). It is possible that manual instrumentation of the device may be sufficient to elicit some vagal activity, but this is considered minimal and is comparable across both device types. Providing that these challenges can be addressed, nVNS may provide a practical approach to stimulating the vagus nerve in the pre- and post-operative setting.

The present study will inform the feasibility of a phase-III randomised controlled trial of nVNS by addressing key challenges in trial design. This includes compliance to the stimulation schedule in a complex clinical setting where unique barriers may exist. It also includes the adequacy of participant blinding. This is important in studies of electrical nerve stimulation where sham-controls may be associated with statistical improvements in clinical outcomes (35). Some issues of feasibility are beyond the scope of this study. The selection of outcomes for ileus are poorly standardised, with over 50 different outcomes identified from previous literature in the last 20 years (36). To address this, a core outcome set for gastrointestinal recovery is under development by an international collaboration of clinicians and patients (37). This will help to standardise reporting in the future. In addition, the feasibility of implementing nVNS into enhanced recovery after surgery protocols across diverse settings will remain

For peer review only

unclear. The present study explores the feasibility of nVNS in two large teaching hospitals, but further consideration of barriers to implementation will be required.

For peer review only

Ethics approval: Research ethics approval was confirmed by the North East - Tyne & Wear South NHS Research Ethics Committee (19/NE/0217) on 2nd July 2019. Written, informed consent to participate will be obtained from all participants.

Availability of data and materials: A model consent form is available (Suppl. File 3). Fully anonymised data will be available upon reasonable request 12 months after the final trial activity.

Competing interests: No competing interests are declared. The views expressed in this publication are those of the authors and not necessarily those of the NHS, the National Institute for Health Research, Health Education England, or the Department of Health.

Funding: This study is funded by a National Institute for Health Research Doctoral Research Fellowship (S Chapman; DRF-2018-11-049). Study devices are provided through the manufacturer's (electroCore™, Inc) Investigator Initiated Trial Programme.

Contributions: SJC and DJ conceptualised the study and all authors (SJC, MN, CJCM, DT, DDS, and DJ) contributed to its design. SJC prepared the protocol draft, which was reviewed and approved by all authors (SJC, MN, CJCM, DT, DDS, and DJ). The Clinical Trials Research Unit at University of Leeds oversees randomisation and University of Leeds is the study sponsor. DDS is the statistical guarantor and DJ is the overall study guarantor.

Copyright: The Corresponding Author has the right to grant on behalf of all authors and does grant on behalf of all authors, a worldwide licence to the Publishers and its licensees in perpetuity, in all forms, formats and media (whether known now or created in the future), to i) publish, reproduce, distribute, display and store the Contribution, ii) translate the Contribution into other languages, create adaptations, reprints, include within collections and create summaries, extracts and/or, abstracts of the Contribution, iii) create any other derivative work(s) based on the Contribution, iv) to exploit all subsidiary rights in the Contribution, v) the inclusion of electronic links from the Contribution to third party material where-ever it may be located; and, vi) licence any third party to do any or all of the above.

Acknowledgements: None

For peer review only

References

1. Scarborough JE. Schumacher J. Kent KC. Heise CP. Greenberg CC. Associations of Specific Postoperative Complications With Outcomes After Elective Colon Resection: A Procedure-Targeted Approach Toward Surgical Quality Improvement. JAMA Surg 2017;152:e164681.
2. Yorkshire Surgical Research Collaborative. Multicentre observational study of gastrointestinal recovery after elective colorectal surgery. Colorectal Dis 2018;20:536-544.
3. Tevis SE. Carchman EH. Foley EF. Harms BA. Heise CP. Kennedy GD. Postoperative Ileus-More than Just Prolonged Length of Stay? J Gastrointest Surg. 2015;19:1684-90.
4. Mao H. Milne TGE. O'Grady G. Vather R. Edlin R. Bissett I. Prolonged Postoperative Ileus Significantly Increases the Cost of Inpatient Stay for Patients Undergoing Elective Colorectal Surgery: Results of a Multivariate Analysis of Prospective Data at a Single Institution. Dis Colon Rectum. 2019;62:631-637.
5. Tiernan J, Cook A, Geh I. et al. Use of a modified Delphi approach to develop research priorities for the association of coloproctology of Great Britain and Ireland. Colorectal Dis 2014;16:965-975.
6. Chapman SJ. Pericleous A. Downey C. Jayne DG. Postoperative ileus following major colorectal surgery. Br J Surg 2018;105:797-810.
7. Herbert G. Perry R. Andersen HK. Atkinson C. Penfold C. Lewis SJ, et al. Early enteral nutrition within 24 hours of lower gastrointestinal surgery versus later commencement for length of hospital stay and postoperative complications. Cochrane Database Syst Rev 2019: CD004080.
8. Short V. Herbert G. Perry R. Atkinson C. Ness AR. Penfold C et al. Chewing gum for postoperative recovery of gastrointestinal function. Cochrane Database Syst Rev 2015: CD006506.
9. Kranke P. Jokinen J. Pace NL. Schnabel A. Hollmann MW. Hahnenkamp K. et al. Continuous intravenous perioperative lidocaine infusion for postoperative pain and recovery. Cochrane Database Syst Rev 2015 :CD009642
10. Chapman SJ. Wells CI. Challenges in ileus research. Colorectal Dis 2018;20:639.
11. Matteoli G. Gomez-Pinilla PJ. Nemethova. A. Di Giovangiulio M. Cailotto C. van Bree SH. et al. A distinct vagal anti-inflammatory pathway modulates intestinal muscularis resident macrophages independent of the spleen. Gut 2014;63:938–948.
12. de Jonge WJ. van der Zanden EP. The FO. Bijlsma MF. van Westerloo DJ. et al. Stimulation of the vagus nerve attenuates macrophage activation by activating the Jak2-STAT3 signalling pathway. Nat Immunol 2005;6:844-851.
13. Bonaz B. Sinniger V. Pellissier S. The Vagus Nerve in the Neuro-Immune Axis: Implications in the Pathology of the Gastrointestinal Tract. Front Immunol 2017;8:1452.

For peer review only

14. Stakenborg N. Wolthuis AM. Gomez-Pinilla PJ. Farro G. Di Giovangiulio M. Bosmans G. et al. Abdominal vagus nerve stimulation as a new therapeutic approach to prevent postoperative ileus. Neurogastro & Motility. 2017;29:e13075.
15. Hong G. Pintea B. Lingohr P. Coch C. Randau T. Schaefer N. et al. Effect of transcutaneous vagus nerve stimulation on muscle activity in the gastrointestinal tract (transVaGa): a prospective clinical trial. International J of Colorectal Dis 2019;34:417–422.
16. Chan AW. Tetzlaff JM. Altman DG, et al. SPIRIT 2013 statement: defining standard protocol items for clinical trials. Ann Intern Med. 2013;158:200-7.
17. Bang H. Ni L. Davis CE. Assessment of blinding in clinical trials. Control Clin Trials 2004;25:143-156.
18. van Bree SH. Bemelman WA. Hollmann MW. Zwinderman AH. Matteoli G. El Temna S. et al. Identification of clinical outcome measures for recovery of gastrointestinal motility in postoperative ileus. Ann Surg 2014;259;708-714.
19. Teare MD. Dimairo M. Shephard N. Hayman A. Whitehead A. Walters SJ. Sample size requirements to estimate key design parameters from external pilot randomised controlled trials: a simulation study. Trials 2014;15:264.
20. Gamble C. Krishan A. Stocken D. Guidelines for the Content of Statistical Analysis Plans in Clinical Trials. JAMA 2017;318:2337-2343.
21. Moher D. Hopewell S. Schulz KF. CONSORT 2010 explanation and elaboration: updated guidelines for reporting parallel group randomised trials. BMJ 2010;340:c869
22. The FO. Bennink RJ. Ankum WM. et al. Intestinal handling-induced mast cell activation and inflammation in human postoperative ileus. Gut 2008;57:33-40.
23. Lange T. Dimitrov S. Born J. Effect of sleep and circadian rhythm on the human immune system. Ann N Y Acad Sci 210;1193:48-59.
24. Baigrie J. Lamont PM. Kwiatkowski D. Dallman MJ. Morris PJ. Systemic cytokine response after major surgery. Br J Surgery 1992;79:757-60.
25. Menys A. Taylor SA. Emmanuel A. Ahmed A. Plumb AA. Odille F. Alam A. Halligan S. Atkinson D. Global small bowel motility: assessment with dynamic MR imaging. Radiology. 2013;269:443-50.
26. Ritchie J. Lewis J. McNaughton Nicholls C. Ormston R. Qualitative Research Practice: A Guide for Social Science Students and Researchers 2nd Ed. Sage. London. 2014
27. Schwenk W. Jacobi C. Mansmann U. Böhm B. Müller JM. Inflammatory response after laparoscopic and conventional colorectal resections - results of a prospective randomized trial. Langenbecks Arch Surg 2000;385:2-9.
28. Venara A. Duchalais E. Dariel A. Aubert P. Durand T. Meurette G. Rolli-Derkinderen M. Hamy A. Neunlist M. Anti-inflammatory Effects of Enhanced Recovery Programs on Early-Stage Colorectal Cancer Surgery. World J Surg 2018;42:953-964.

For peer review only

29. Stakenborg N. Labeeuw E. Gomez-Pinilla PJ. De Schepper S. Aerts R. et al. Preoperative administration of the 5-HT4 receptor agonist prucalopride reduces intestinal inflammation and shortens postoperative ileus via cholinergic enteric neurons. Gut 2019;68:1406-1416.
30. Chapman SJ. Czoski Murray CJ. Lonsdale MDS. Boyes S. Tiernan JP. et al. Information needs for recovery after colorectal surgery: Patient focus group study. *Colorectal Dis* 2020 (In Press).
31. Brock C. Brock B. Aziz Q. Møller HJ. Pfeiffer Jensen M. Drewes AM. Farmer AD. Transcutaneous cervical vagal nerve stimulation modulates cardiac vagal tone and tumor necrosis factor-alpha. Neurogastroenterol Motil 2017;29. doi: 10.1111/nmo.12999.
32. Lerman I. Hauger R. Sorkin L. Proudfoot J. Davis B. et al. Noninvasive Transcutaneous Vagus Nerve Stimulation Decreases Whole Blood Culture-Derived Cytokines and Chemokines: A Randomized, Blinded, Healthy Control Pilot Trial. Neuromodulation 2016;19:283-90.
33. Vecchio E. Bassez I. Ricci K. Tassorelli C. Liebler E. et al. Effect of Non-invasive Vagus Nerve Stimulation on Resting-State Electroencephalography and Laser-Evoked Potentials in Migraine Patients: Mechanistic Insights. Front Hum Neurosci 2018;13;12:366.
34. Nonis R. D'Ostilio K. Schoenen J. Magis D. Evidence of activation of vagal afferents by non-invasive vagus nerve stimulation: An electrophysiological study in healthy volunteers. *Cephalgia* 2017;37:1285-1293.
35. Tan K. Wells CI. Dinning P. Bissett IP. O'Grady G. Placebo Response Rates in Electrical Nerve Stimulation Trials for Fecal Incontinence and Constipation: A Systematic Review and Meta-Analysis. Neuromodulation 2019. doi: 10.1111/ner.13092. [Epub ahead of print]
36. Chapman SJ. Thorpe G. Vallance AE. Harji DP. Lee MJ. et al. Systematic review of definitions and outcome measures for return of bowel function after gastrointestinal surgery. BJS Open 2018;3:1-10.
37. Chapman SJ. Lee MJ. Blackwell S. Arnott R. Ten Broek RPG. et al. Establishing core outcome sets for gastrointestinal recovery in studies of postoperative ileus and small bowel obstruction: protocol for a nested methodological study. Colorectal Dis. 2019. doi: 10.1111/codi.14899. [Epub ahead of print]

For peer review only

Table 1 – Eligible Procedures

**Right sided resection**	**Left sided resection**
Ileocolic/caecal resection	Extended left hemicolectomy
Right hemicolectomy	Left hemicolectomy
Extended right hemicolectomy	Sigmoid colectomy
Transverse colectomy	Rectosigmoid colectomy
**Miscellaneous (side grouped accordingly)**	Anterior resection*
Other segmental colonic resection	

For peer review only

Table 2 – Progression criteria

Criteria	Stop	Adjust	Progress
Proportion of eligible patients identified from screening logs	<10%	10-20%	>20%
Number of eligible patients randomised over 24 months (site: SJUH)	≤2 per month	3-4 per month	≥5 per month
Number of eligible patients randomised over 18 months (site: BRI)	<1 per month	1-2 per month	≥3 per month
Adequacy of participant blinding (according to Bang Blinding Index)	Index < -0.5 Or Index > 0.5	Index -0.2 to -0.5 Or Index 0.2 to 0.5	Index 0 to -0.19 Or Index 0 to 0.19
Average compliance to the study treatment schedule *	<10/20 stimulations across 10 days	10-15/20 stimulations across 10 days	≥16/20 stimulations across 10 days
Proportion of missing clinical outcome data	>40%	15-30%	<15%
Proportion of randomised patients lost to follow up	>40%	15-30%	<15%
Incidence of complications or serious complications	>20% increase in complications	5-20% increase in complications	<5% increase in complications

BRI: Bradford Royal Infirmary, Bradford; SJUH: St. James's University Hospital, Leeds

For peer review only

Table 3 – Schedule of Events

	Events	Baseline	Preop study visit	5 days before surgery	Day of surgery (pre-op)	Day of surgery (Intraop)	POD 1	POD 2	POD 3	POD 4	POD 5	POD 6-10	After discharge	30-day phone F/U
**Study Procedures**	Trial consent	✓												
	Device training		✓											
	Device distribution		✓		✓									
	Self-administered stimulation			✓			✓	✓	✓	✓	✓			
	Stimulation diary			✓			✓	✓	✓	✓	✓			
	Blinding poll										✓			
**Data collection**	Eligibility CRF	✓												
	Baseline CRF	✓												
	Operative CRF					✓								
	Gastrointestinal Function CRF						✓	✓	✓	✓	✓	✓		
	Follow-up CRF													✓
**Sub-studies***	*Optional sub-study consents*	✓												
	Inflammatory response study<br>Blood only: ✓; Blood and fluid ✓✓				✓	✓✓	✓		✓					
	Small bowel motility study (MRI)		✓						✓					
	Qualitative sub-study (participant or non-participant interview)												✓	

**Sub-studies are optional and undertaken at the lead site only*
CRF: Case Report Form; F/U: follow up; MRI: magnetic resonance imaging; POD: postoperative day

For peer review only

Table 4 – Sub-study additional exclusion criteria

**Inflammatory response sub-study**
• Regular use of non-steroidal anti-inflammatory drugs in the preceding 7 days<br>• Medical condition requiring oral or injectable steroids<br>• Neoadjuvant chemoradiotherapy within 12 months prior to surgery
**Bowel motility sub-study**
• Indwelling pacemaker or cardiac defibrillator device<br>• Non-MR compatible metallic implants, prostheses, neurosurgical clips, indwelling stimulator or pump devices, which would preclude safe MRI<br>• Claustrophobia<br>• Metallic foreign bodies in the eyes

MRI: Magnetic Resonance Imaging

For peer review only

Figure 1 – Study flow chart

For peer review only

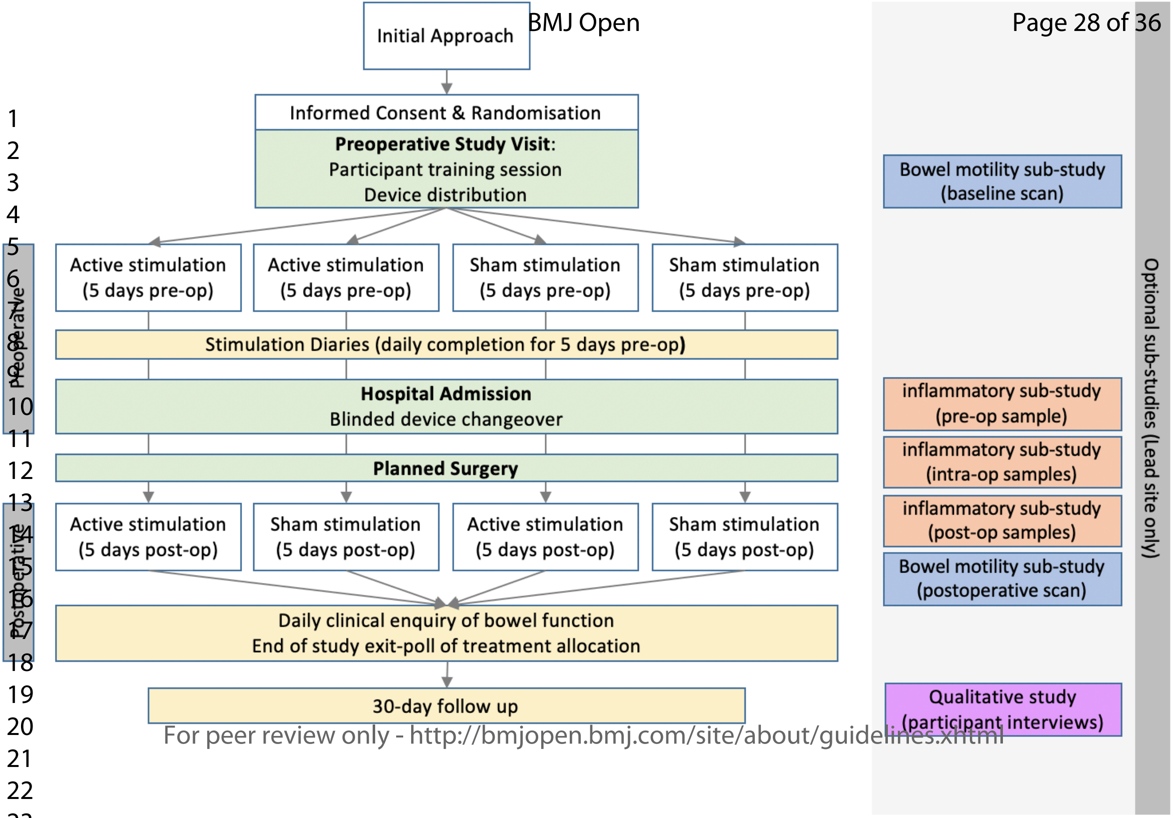
Initial Approach
Informed Consent & Randomisation
Preoperative Study Visit:
Participant training session
Device distribution
Preoperative
Active stimulation (5 days pre-op)
Active stimulation (5 days pre-op)
Sham stimulation (5 days pre-op)
Sham stimulation (5 days pre-op)
Stimulation Diaries (daily completion for 5 days pre-op)
Hospital Admission
Blinded device changeover
Planned Surgery
Postoperative
Active stimulation (5 days post-op)
Sham stimulation (5 days post-op)
Active stimulation (5 days post-op)
Sham stimulation (5 days post-op)
Daily clinical enquiry of bowel function
End of study exit-poll of treatment allocation
30-day follow up
Bowel motility sub-study (baseline scan)
inflammatory sub-study (pre-op sample)
inflammatory sub-study (intra-op samples)
inflammatory sub-study (post-op samples)
Bowel motility sub-study (postoperative scan)
Qualitative study (participant interviews)
Optional sub-studies (Lead site only)

Supplementary File

Study Status

The study first began recruitment on 2nd January 2020 and is expected to complete by 31st August 2021. The current protocol version is v1.2 (13th February 2020).

Auditing

The conduct of the trial will be audited monthly by the principal investigator. In line with Good Clinical Practice, a study site folder will be held at each participating recruitment site. This will contain the current study protocol, delegation log outlining responsibilities of all research team members, and all study correspondence. Data relating to recruitment will be uploaded to the NIHR Central Portfolio Management System on a monthly basis.

Adverse Events

The reporting of adverse events will be done in line with Good Clinical Practice. All serious adverse events will be reported to the sponsor within 24 hours of research staff becoming aware of the event. Serious adverse events that are considered to be unexpected and related to the study will be escalated to the research ethics committee.

Study Amendments

If amendments to the study protocol or documents are required, this will be done and communicated in line with Health Research Authority guidelines. All amendments will be categorised and submitted for review by the Research Ethics Committee, as appropriate.

Dissemination Plan

The findings of the study and sub-studies will be disseminated at academic conferences and in peer-reviewed manuscripts. The results will also be summarised in plain English visual abstracts and disseminated to research participants and the public.

For peer review only

Study Sponsor

The sponsor for this study is University of Leeds

Recruitment & Protocol Materials

Study recruitment and protocol materials are available upon request from the corresponding author.

For peer review only

SPIRIT
STANDARD PROTOCOL ITEMS: RECOMMENDATIONS FOR INTERVENTIONAL TRIALS

SPIRIT 2013 Checklist: Recommended items to address in a clinical trial protocol and related documents*

Section/item	Item No	Description
**Administrative information**		
Title	1	Descriptive title identifying the study design, population, interventions, and, if applicable, trial acronym **(Page 1)**
Trial registration	2a	Trial identifier and registry name. If not yet registered, name of intended registry **(Page 6)**
	2b	All items from the World Health Organization Trial Registration Data Set **(Page 6)**
Protocol version	3	Date and version identifier **(Suppl. File 1)**
Funding	4	Sources and types of financial, material, and other support **(Page 18)**
Roles and responsibilities	5a	Names, affiliations, and roles of protocol contributors **(Page 18)**
	5b	Name and contact information for the trial sponsor **(Suppl. File 1)**
	5c	Role of study sponsor and funders, if any, in study design; collection, management, analysis, and interpretation of data; writing of the report; and the decision to submit the report for publication, including whether they have ultimate authority over any of these activities **(Suppl. File 1)**
	5d	Composition, roles, and responsibilities of the coordinating centre, steering committee, endpoint adjudication committee, data management team, and other individuals overseeing trial, if applicable (see Item 21a for data monitoring committee) **(Suppl. File 1)**
**Introduction**		
Background and rationale	6a	Description of research question and justification for undertaking the trial, including summary of relevant studies (published and unpublished) examining benefits and harms for each intervention **(Page 4-5)**
	6b	Explanation for choice of comparators **(Page 4-5)**
Objectives	7	Specific objectives or hypotheses **(Page 6)**

For peer review only

Trial design	8	Description of trial design including type of trial (eg, parallel group, crossover, factorial, single group), allocation ratio, and framework (eg, superiority, equivalence, noninferiority, exploratory) **(Page 6)**

Methods: Participants, interventions, and outcomes

Study setting	9	Description of study settings (eg, community clinic, academic hospital) and list of countries where data will be collected. Reference to where list of study sites can be obtained **(Page 7)**
Eligibility criteria	10	Inclusion and exclusion criteria for participants. If applicable, eligibility criteria for study centres and individuals who will perform the interventions (eg, surgeons, psychotherapists) **(Page 7)**
Interventions	11a	Interventions for each group with sufficient detail to allow replication, including how and when they will be administered **(Page 8)**
	11b	Criteria for discontinuing or modifying allocated interventions for a given trial participant (eg, drug dose change in response to harms, participant request, or improving/worsening disease) **(Page 8)**
	11c	Strategies to improve adherence to intervention protocols, and any procedures for monitoring adherence (eg, drug tablet return, laboratory tests) **(Page 10)**
	11d	Relevant concomitant care and interventions that are permitted or prohibited during the trial **(Page 8)**
Outcomes	12	Primary, secondary, and other outcomes, including the specific measurement variable (eg, systolic blood pressure), analysis metric (eg, change from baseline, final value, time to event), method of aggregation (eg, median, proportion), and time point for each outcome. Explanation of the clinical relevance of chosen efficacy and harm outcomes is strongly recommended **(Page 9)**
Participant timeline	13	Time schedule of enrolment, interventions (including any run-ins and washouts), assessments, and visits for participants. A schematic diagram is highly recommended (see Figure) **(Page 9-10)**
Sample size	14	Estimated number of participants needed to achieve study objectives and how it was determined, including clinical and statistical assumptions supporting any sample size calculations **(Page 10)**
Recruitment	15	Strategies for achieving adequate participant enrolment to reach target sample size **(Page 10)**

Methods: Assignment of interventions (for controlled trials)

Allocation:

Sequence generation	16a	Method of generating the allocation sequence (eg, computer-generated random numbers), and list of any factors for stratification. To reduce predictability of a random sequence, details of any planned restriction (eg, blocking) should be provided in a separate document that is unavailable to those who enrol participants or assign interventions **(Page 10)**
Allocation concealment mechanism	16b	Mechanism of implementing the allocation sequence (eg, central telephone; sequentially numbered, opaque, sealed envelopes), describing any steps to conceal the sequence until interventions are assigned **(Page 10)**
Implementation	16c	Who will generate the allocation sequence, who will enrol participants, and who will assign participants to interventions **(Page 10)**
Blinding (masking)	17a	Who will be blinded after assignment to interventions (eg, trial participants, care providers, outcome assessors, data analysts), and how **(Page 11)**
	17b	If blinded, circumstances under which unblinding is permissible, and procedure for revealing a participant's allocated intervention during the trial **(Page 11)**

Methods: Data collection, management, and analysis

Data collection methods	18a	Plans for assessment and collection of outcome, baseline, and other trial data, including any related processes to promote data quality (eg, duplicate measurements, training of assessors) and a description of study instruments (eg, questionnaires, laboratory tests) along with their reliability and validity, if known. Reference to where data collection forms can be found, if not in the protocol **(Page 11)**
	18b	Plans to promote participant retention and complete follow-up, including list of any outcome data to be collected for participants who discontinue or deviate from intervention protocols **(Page 11)**
Data management	19	Plans for data entry, coding, security, and storage, including any related processes to promote data quality (eg, double data entry; range checks for data values). Reference to where details of data management procedures can be found, if not in protocol **(Page 11)**
Statistical methods	20a	Statistical methods for analysing primary and secondary outcomes. Reference to where other details of the statistical analysis plan can be found, if not in the protocol **(Page 11)**
	20b	Methods for any additional analyses (eg, subgroup and adjusted analyses) **(Page 11)**

	20c	Definition of analysis population relating to protocol non-adherence (eg, as randomised analysis), and any statistical methods to handle missing data (eg, multiple imputation) **(Page 11-12)**
**Methods: Monitoring**		
Data monitoring	21a	Composition of data monitoring committee (DMC); summary of its role and reporting structure; statement of whether it is independent from the sponsor and competing interests; and reference to where further details about its charter can be found, if not in the protocol. Alternatively, explanation of why a DMC is not needed **(Suppl. File 1)**
	21b	Description of any interim analyses and stopping guidelines, including who will have access to these interim results and make the final decision to terminate the trial **(Suppl. File 1)**
Harms	22	Plans for collecting, assessing, reporting, and managing solicited and spontaneously reported adverse events and other unintended effects of trial interventions or trial conduct **(Suppl. File 1)**
Auditing	23	Frequency and procedures for auditing trial conduct, if any, and whether the process will be independent from investigators and the sponsor **(Suppl. File 1)**
**Ethics and dissemination**		
Research ethics approval	24	Plans for seeking research ethics committee/institutional review board (REC/IRB) approval **(Page 18)**
Protocol amendments	25	Plans for communicating important protocol modifications (eg, changes to eligibility criteria, outcomes, analyses) to relevant parties (eg, investigators, REC/IRBs, trial participants, trial registries, journals, regulators) **(Suppl. File 1)**
Consent or assent	26a	Who will obtain informed consent or assent from potential trial participants or authorised surrogates, and how (Item 32) **(Page 10)**
	26b	Additional consent provisions for collection and use of participant data and biological specimens in ancillary studies **(Page 18)**
Confidentiality	27	How personal information about potential and enrolled participants will be collected, shared, and maintained in order to protect confidentiality before, during, and after the trial **(Page 11)**
Declaration of interests	28	Financial and other competing interests for principal investigators for the overall trial and each study site **(Page 18)**
Access to data	29	Statement of who will have access to the final trial dataset, and disclosure of contractual agreements that limit such access for investigators**(Page 18)**
Ancillary and post-trial care	30	Provisions, if any, for ancillary and post-trial care, and for compensation to those who suffer harm from trial participation **(N/A)**

For peer review only

Dissemination policy	31a	Plans for investigators and sponsor to communicate trial results to participants, healthcare professionals, the public, and other relevant groups (eg, via publication, reporting in results databases, or other data sharing arrangements), including any publication restrictions **(Page 12)**
	31b	Authorship eligibility guidelines and any intended use of professional writers **(Suppl. File 1)**
	31c	Plans, if any, for granting public access to the full protocol, participant-level dataset, and statistical code **(Suppl. File 1)**
**Appendices**		
Informed consent materials	32	Model consent form and other related documentation given to participants and authorised surrogates **(Suppl. File 1)**
Biological specimens	33	Plans for collection, laboratory evaluation, and storage of biological specimens for genetic or molecular analysis in the current trial and for future use in ancillary studies, if applicable **(Pages 12-14)**

*It is strongly recommended that this checklist be read in conjunction with the SPIRIT 2013 Explanation & Elaboration for important clarification on the items. Amendments to the protocol should be tracked and dated. The SPIRIT checklist is copyrighted by the SPIRIT Group under the Creative Commons "Attribution-NonCommercial-NoDerivs 3.0 Unported" license.

For peer review only

UNIVERSITY OF LEEDS

Vagus nerve stimulation to improve bowel function after surgery

Participant Study ID	Patient Initials	Patient Date of Birth

Agreement to the following are required to participate in the study

Please <u>initial</u> the boxes to indicate your agreement:

A. I confirm that I have read the patient information sheet dated [date, version] and have had the opportunity to ask questions. ☐

B. I understand that my participation in all aspects of this study is voluntary and that I may freely withdraw at any time, without giving a reason and without my medical care or rights being affected. I understand that data already collected before withdrawal will be used, unless I request otherwise. ☐

C. I understand that sections of my medical records may be looked at by responsible individuals from the lead study team, the University of Leeds, and from regulatory authorities where it is relevant to my taking part in this research study. ☐

D. I agree to allow the information collected about me and a copy of this consent form to be passed to the University of Leeds upon it being handled securely ☐

E. I agree to allow information or results arising from this study to be used for future medical research on the understanding that my identity will remain anonymous. ☐

F. I agree for my GP to be informed about my participation in this study ☐

G. I agree to be contacted by telephone and/or in writing with information that may be relevant to my taking part in the research using information held at the hospital ☐

H. I agree to take part in the above study. ☐

For peer review only - http://bmjopen.bmj.com/site/about/guidelines.xhtml

UNIVERSITY OF LEEDS

Participant Study ID	Patient Initials	Patient Date of Birth

The following criteria are optional and relevant to patients having their operation at St. James's University Hospital (SJUH) only

Do you wish to participate in the optional part of this study (involving blood and fluid tests)? (please tick as appropriate):

☐ Yes (see box) ☐ No ☐ Surgery not at SJUH

If you ticked ***Yes*** *above, please initial the boxes to indicate your agreement:*

I. I understand that all samples will be stored, analysed, and destroyed at the University of Leeds according to regulations set by the Health Tissue Authority. ☐

J. I know that I may freely withdraw from this optional study without affecting my participation in the main study, my medical care, or my legal rights. ☐

K. I understand that samples will be stored confidentially and that my name and personal details will not be stored with my samples. I understand that samples collected prior to withdrawal will be retained unless I request otherwise. ☐

L. I agree to allow data and samples from this study to be used for future medical research on the understanding that my identity will remain anonymous. ☐

Name of treating hospital/research site
(BLOCK CAPITALS)

Name of participant (BLOCK CAPITALS)	Date (dd/mm/yyyy)	Signature

Name of researcher taking consent (BLOCK CAPITALS)	Date (dd/mm/yyyy)	Signature

One copy of this consent form should be retained by the recruiting team and another copy given to the participant

For peer review only - http://bmjopen.bmj.com/site/about/guidelines.xhtml
For peer review only
